# A Bayesian filtering method for estimating the fitness effects of nascent adaptive mutations

**DOI:** 10.1101/2025.03.29.646120

**Published:** 2025-04-03

**Authors:** Huan-Yu Kuo, Sergey Kryazhimskiy

## Abstract

The distribution of fitness effects (DFE) of new beneficial mutations is a key quantity that dictates the dynamics of adaptation. The recently developed barcode lineage tracking (BLT) approach is an important advance toward measuring DFEs. BLT experiments enable researchers to track the frequencies of ~10 ^5^ of barcoded lineages in large microbial populations and detect up to thousands of nascent beneficial mutations in a single experiment. However, reliably identifying adapted lineages and estimating the fitness effects of the driver mutations remains a challenge because lineage dynamics are subject to demographic and measurement noise and competition with other lineages. We show that the currently standard Levy-Blundell method for analyzing BLT data can be biased in certain regimes. To address this problem, we develop a new method called BASIL (BAyesian Selection Inference for Lineage tracking data), which dynamically updates the belief distribution of each lineage's fitness and size based on the number of barcode reads. We calibrate BASIL's model of noise with new experimental data and find that noise variance scales non-linearly with lineage abundance. We test BASIL's performance on simulated data and data from published BLT studies and find that it is both robust and accurate. Finally, we characterize how BASIL's power to identify adapted lineages depends on the frequency of sampling and read coverage. Our work paves the way for a systematic inference of the distribution of fitness effects of new beneficial mutations from BLT experiments in a variety of scenarios.

## INTRODUCTION

Every new adaptation originates in a single individual. Most vanish soon thereafter. The few that survive may encounter fierce competition with other contending adapted lineages [1–5]. This competition—termed "clonal interference"—is particularly severe in organisms that have limited recombination and also have a large supply of beneficial mutations, such as many bacteria [6–8], cancers [9], and viruses [10–12]. In the clonal interference regime, the chance of fixation of a new allele depends not only on its own fitness benefit but also on the benefits provided by competing alleles [13–15]. Thus, to understand the dynamics of rapid adaptation, we must know the statistics of fitness effects of adaptive mutations [15,16]. However, this empirical knowledge is not yet readily available.

Measuring the effects of new adaptive mutations is challenging. The most statistically sound strategy would be to isolate them from random samples of spontaneous mutations. However, in most organisms, mutations are rare [17], and beneficial mutations typically constitute a relatively small fraction of all mutations [3,18,19] (but see [20,21]). As a result, obtaining accurate statistics of beneficial mutations from samples of random mutations is generally extremely inefficient. Instead, to sample adaptive mutations, researchers leverage the power of natural selection because it elevates the frequencies of beneficial mutations in a population. It is relatively easy to find and isolate beneficial mutations from recently adapted populations, particularly those evolved in the lab [22–25]. However, this strategy preferentially captures mutations with the strongest fitness benefits because they are least likely to be lost by genetic drift or clonal interference [3]. Therefore, this approach may provide an incomplete and biased view of the diversity of adaptive mutations.

The recently developed barcode lineage tracking (BLT) method is a powerful strategy, applicable in asexual genetically tractable microbes, that allows one to estimate the fitness effects of many nascent beneficial mutations including weak ones [3–5,26–31]. The key idea is to introduce many (typically $\sim 10^5$) random neutral DNA barcodes into an otherwise isogenic population and track barcode frequencies over time using deep sequencing. Each new adaptive mutation that arises in the population is permanently linked to a unique DNA barcode. As an adaptive mutation spreads in the population, the corresponding increase in the frequency of the linked barcode can be detected in the sequencing data. Moreover, since the sizes of most barcoded subpopulations are initially small (typically ~100 individuals), the expansion of each adapted subpopulation is initially driven by a single adaptive mutation. In other words, for a period of time, each barcode with increasing frequency reports on a single adaptive driver mutation. As a result, this approach allows one to measure the fitness benefits of many simultaneously segregating driver mutations, including weak ones, in a single relatively short evolution experiment, while avoiding laborious genetic reconstructions and screens [3,32]. Although the BLT approach does not entirely eliminate the biases introduced by natural selection, as mutations with weaker benefits take longer to establish and are more difficult to detect, it significantly mitigates them, and overall provides a much more accurate picture of the diversity of adaptive mutations than other existing methods [3].

While the BLT approach significantly advances our ability to capture and study adaptive mutations, analyzing BLT experiments remains challenging. The main challenge is to distinguish lineages that have acquired a single beneficial mutation ("adapted lineages") from those that have not ("neutral lineages") based on noisy dynamics of low-abundance barcodes. The core idea is the same as in classical competition assays [33–36], i.e., to determine whether a lineage systematically increases in frequency relative to neutral reference lineages (i.e., those with wildtype fitness) and then to infer the fitness effect of the underlying driver mutation from the rate of this increase.

Despite many similarities, the BLT setup differs in at least three important ways from the canonical competition assay. First, in competition assays, lineages are usually represented by thousands of cells at the beginning of the experiment, whereas in the BLT experiments all lineages are by design present at low abundances (~100 cells), which makes lineage extinctions much more likely. Second, competition assays are typically short (≤ 50 generations) to prevent new mutations from significantly affecting lineage dynamics, whereas BLT experiments are longer (> 100 generations) precisely to allow for new adaptive mutations to arise and reach sufficiently high frequencies. Third, in competition assays, it is known a priori which lineages are neutral and which ones carry mutations; so, the choice of reference lineages is not in question. In contrast, in the BLT setup, adaptive mutations can arise during the experiment itself (or shortly before), and which lineages will become adapted and which ones remain neutral is a priori unknown. Thus, how to choose reference lineages is not obvious. Levy et al, who were the first to develop a statistical approach for analyzing BLT data (which we refer to as the "Levy-Blundell method"), chose lineages represented by about 20 to 50 cells at the bottleneck as the neutral reference because such low-abundance lineages were unlikely to acquire adaptive mutations in their experiment [3]. However, whether this choice is universally good is unclear.

The general challenge with BLT experiments is that lineages chosen as the neutral references may go extinct over the course of the experiment (especially if they are at low abundance) or acquire adaptive mutations (especially if they are highly abundant). Both outcomes are undesirable since they can bias the estimates of fitness effects of adaptive mutations. However, it is unclear how serious this problem is in various experimental conditions (e.g., strong versus weak selection) and with various choices of reference lineages (e.g., in terms of their abundances). A priori, we might expect the performance of the Levy-Blundell method to be diminished when adaptation is fast, because low-abundance reference lineages might rapidly go extinct and high-abundance reference lineages might rapidly acquire beneficial mutations.

To assess the performance of the Levy-Blundell method, we applied its simplified version which retains its key properties (referred to as the "neutral decline" method to distinguish it from the original approach) to data from simulated BLT experiments under weak and strong selection. We find that this method indeed can produce biased fitness estimates depending on the selection regime and the choice of reference lineages. We then develop a new method termed BASIL (BAyesian Selection Inference for Lineage tracking data) that avoids the explicit designation of neutral reference lineages by the user. Instead, BASIL keeps track of belief distributions for the fitness of all lineages under the assumption that lineages are expected to be neutral at the initial time point. It updates these distributions based on barcode read counts, and uses those lineages as reference whose fitness is already estimated with high confidence. Our approach combines several techniques developed previously in different methods for analyzing competition assays [36–39] and BLT experiments [3,5]. In particular, similar to previous methods, BASIL models the evolutionary dynamics of lineages between sampling time points as well as the measurement process, which we parameterize using data from a new calibration experiment. In contrast to most other approaches and similar to Ref. [38], BASIL treats unobserved lineage sizes as hidden variables, which turns out to be particularly important for obtaining unbiased estimates of lineage fitness under strong selection. Finally, similar to Ref. [36], we cast our model in the Bayesian framework, which allows us to keep track of uncertainties in our fitness and lineage size estimates and dynamically update our belief distributions as the data comes in. We characterize the performance of BASIL on simulated and published BLT datasets.

## RESULTS

### Neutral decline method can produce biased estimates of fitness effects

To understand how well we can infer the fitness effects (also referred to as selection coefficients) of nascent adaptive mutations using the basic approach described above, we simulate a BLT experiment with 100,000 barcoded lineages, 3,000 of which are adapted (see Supplementary Information, Section 3 for details). In other words, all beneficial mutations are pre-existing in our simulations, which represents the simplest scenario where the inference methods are expected to perform best. However, we note that, in contrast to competition assays, our algorithms are not aware of which lineages are adapted or not. We simulate two conditions that we refer to as "strong selection" and "weak selection" (Figure S1 and Table S3). In the weak selection regime, which resembles the original BLT experiment carried out by Levy et al [3], the average fitness of an adapted lineage is 3%. As a result, the mean fitness of the population increases by about 4% in 150 generations. In the strong selection regime, the average fitness of an adapted lineage is 8%, and the population's mean fitness increases by about 12% in 150 generations.

We first analyze these simulated datasets using the "neutral decline" method, which retains key features of the Levy-Blundell method [3] but simplifies it (see Supplementary Information, Section 4.2). Briefly, we select either low or high abundance lineages as our neutral reference. Low-abundance lineages are those represented by 20 to 40 reads, which corresponds to 20 to 40 cells at the bottleneck and high-abundance lineages are those represented by 80–100 reads, corresponding to 80 to 100 cells at the bottleneck. We then infer the population mean fitness $\bar{s}_k$ at each sampling interval $(t_{k-1}, t_k)$ from equation

$$\frac{\bar{r}_k}{R_k} = \frac{r_{k-1}}{R_{k-1}} e^{-\bar{s}_k \Delta t_k}, \quad (1)$$

which follows from the standard population genetics theory [40] and which we refer to as the "neutral decline equation". Here, $\bar{r}_k$ is the average read count at time $t_k$ for those reference lineages that had exactly $r_{k-1}$ reads observed at time $t_{k-1}$. $R_k$ is the total read depth at time $t_k$ and $\Delta t_k = t_k - t_{k-1}$. Given the mean fitness trajectory $\bar{s}_k$, we then infer the selection coefficients of all non-reference lineages by maximizing the likelihood of the lineage's frequency trajectory.

The neutral decline method is simplified in two ways compared to the Levy-Blundell method. First, in the Levy-Blundell model, adapted lineages are characterized by two parameters, the selection coefficient $s$ and the establishment time $\tau$, whereas our model has only one parameter $s$ per lineage. However, in our simulations, all adapted mutations are pre-existing, implying that our simpler one-parameter model is sufficient. The second difference is that, in addition to the mean fitness $\bar{s}_k$, Levy et al also estimate the noise parameter $\kappa$. To infer both $\bar{s}_k$ and $\kappa$, they use a model that describes how the entire distribution of read counts changes over time (see equation (45) in the Supplementary material to Ref. [3]). The mean $\bar{r}_k$ of this distribution is described by equation (1), which does not depend on $\kappa$. Thus, equation (1) is sufficient if we are interested only in $\bar{s}_k$ (Figure S2). Importantly, since equation (1) directly follows from the model used by Levy et al, a failure of equation (1) to capture the relationship between $r_{k-1}$ and $\bar{r}_k$ in some regimes would imply a failure of the full model. Therefore, we focus our analysis on this most basic version of the method, which allows us to more easily determine when and why its core assumptions fail.

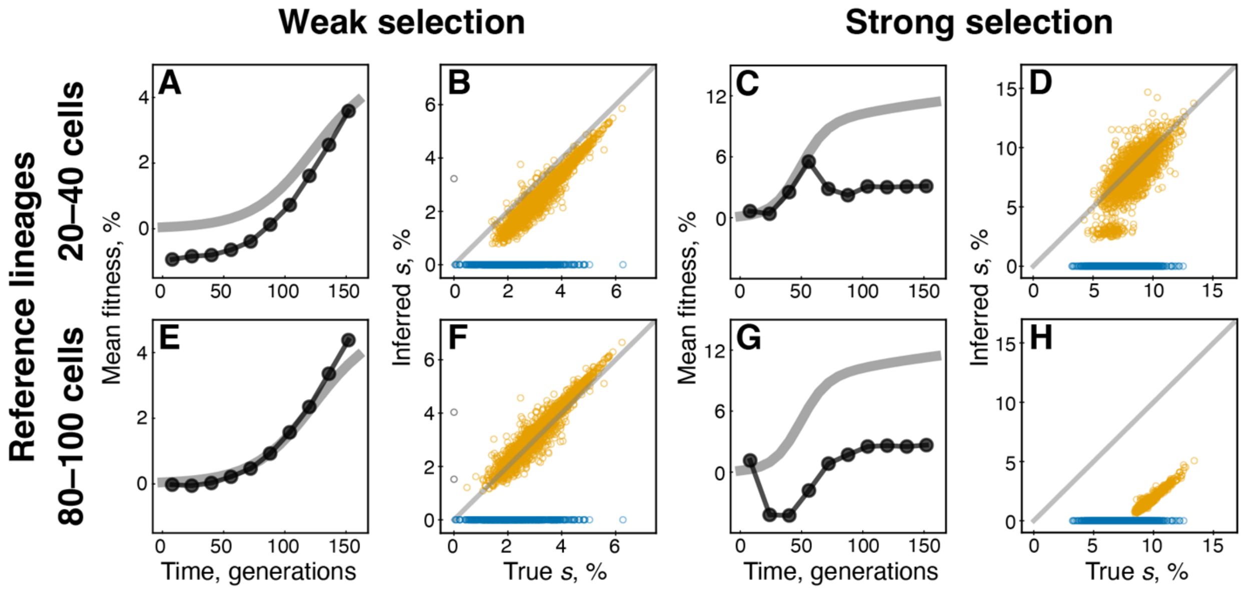

**Figure 1. Performance of the neutral decline method is sensitive to the strength of selection and the choice of reference lineages.** Left panels A, B, E, F show simulations in the weak selection regime; right panels C, D, G, H show simulations in the strong selection regime. Top panels A, B, C, D show inference results when low-abundance lineages (those with 20–40 cells) are used as reference. Bottom panels E, F, G, H show inference results when high-abundance lineages (those with 80–100 cells) are used as reference. **A, C, E, G.** True (thick gray lines) and inferred (black circles) mean fitness trajectories. **B, D, F, H.** True versus inferred selection coefficients of adapted lineages. Orange, blue and black points represent true positives, false negatives and false positives, respectively.

The performance of the neutral decline method on our BLT simulations is illustrated in Figure 1. In the weak selection regime, we find that the mean fitness trajectory and the fitness effects of individual adaptive mutations are inferred accurately as long as we use high-abundance lineages as the neutral reference (Figure 1E,F). In contrast, when low-abundance lineages are used, the neutral decline approach slightly underestimates both the mean fitness of the population and the selection coefficients of individual adaptive mutations (Figure 1A,B). The neutral decline approach performs significantly worse in the strong selection regime (Figure 1C,D,G,H). If low-abundance lineages are used as reference, estimates of mean fitness are accurate only for the first ~50 generations (Figure 1C), which results in noisy estimates of selection coefficients of individual adaptive mutations (Figure 1D). When high-abundance lineages are used as reference, the neutral decline method severely underestimates both the mean fitness and the effects of individual mutations (Figure 1G,H). These results indicate that the choice of reference lineages is critical for the accuracy of the neutral decline method and that the optimal choice depends on the condition under which the BLT experiment is carried out.

To understand the reasons for this undesirable behavior of the neutral decline approach, we plotted the rescaled average read count $\tilde{r}_k = \bar{r}_k R_{k-1}/R_k$ at time point $t_k$ against the observed read count $r_{k-1}$ at the previous time point. According to equation (1) or its generalization for adapted lineages (equation (S8)), $\tilde{r}_k$ must be proportional to $r_{k-1}$ with a zero $y$-intercept. Instead, we find that this relationship is more complex, with its shape being dependent on the regime (strong versus weak selection) and on time (Figure 2A,C). Specifically, under strong selection, the relationship becomes non-linear at later time points, and approaches linearity only for lineages with very low frequencies (Figure 2C). When selection is sufficiently weak, $\tilde{r}_k$ relates linearly to $r_{k-1}$ during the entire BLT experiment, but with a non-zero $y$-intercept (Figure 2A). Despite its relatively small

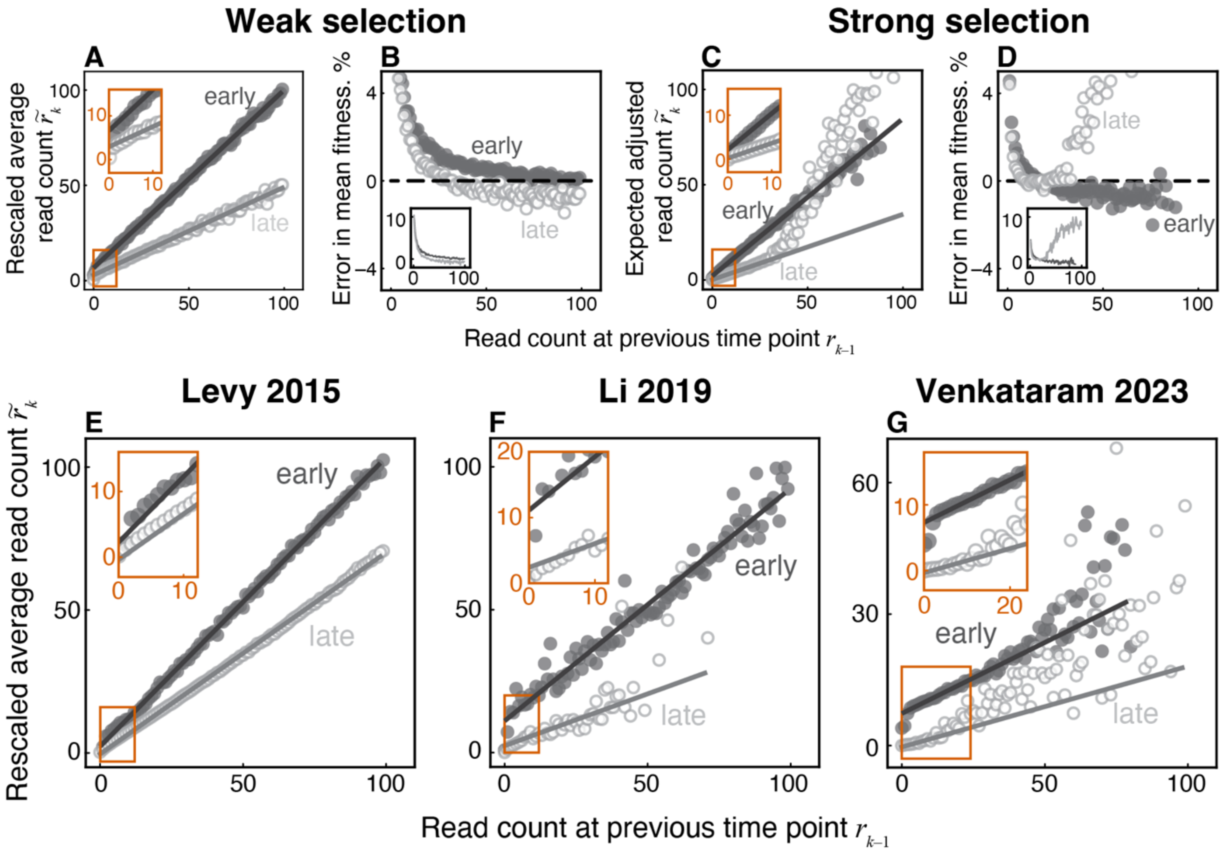

**Figure 2. The observed lineage read counts deviate from those predicted by equation (1) both in simulated and real data. A.** Rescaled average read count $\tilde{r}_k = \bar{r}_k R_{k-1}/R_k$ at the next time point (ordinate) plotted against the read count observed at the previous time point $r_{k-1}$ (abscissa) for the weak-selection simulation. Light and dark gray points represent early ($t_k = 16$ generations) and late ($t_k = 160$ generations) time intervals. Lines represent least-squares best fits within the linear regime (see Supplementary Information Section 4.4). **B.** Error in the inferred mean fitness (true minus inferred) as a function of the read count of lineages that are used as the neutral reference. Shades are the same as in panel A. **C.** Same as panel A but for the strong selection regime. Early time interval is at $t_k = 16$ generations and late time interval is at $t_k = 64$ generations. **D.** Same as panel B but for the strong selection regime. **E–G.** Same as panel A but for three real BLT datasets, as indicated. Early time interval is at $t_k = 16, 7, 20$ generations in panels E, F, G, respectively and late time interval is at $t_k = 112, 112, 86$ generations.

value (e.g., 6.60 at generation 16 and 2.89 at generation 160), a non-zero $y$-intercept can significantly bias the inference of mean fitness, especially when low-abundance lineages are chosen as reference. For example, in the weak selection regime, if we use lineages present at $r_0 = 20$ reads (represented by 20 cells), the inferred mean fitness is –1.653% relative to the wildtype whereas its true value is +0.060% (Figures 1A and 2B). Since the estimates of selection coefficients of all adaptive mutations depend on the mean fitness estimate, this error results in biased estimates of selection coefficients of all lineages, as seen in Figure 1B. Choosing initially more abundant lineages as reference (e.g., those with $r_0 = 80$ reads, represented by 80 cells) yields a more accurate estimate of initial mean fitness (Figures 1E and 2B) because such estimates are less sensitive to the value of the $y$-intercept (Figure 2A). However, which lineages provide the most accurate estimate changes over time. For example, by generation 160, the most accurate inference of mean fitness is achieved by lineages with about 30 reads (corresponding to 30 cells at bottleneck; Figure 2B).

We next asked whether the relationship between read counts at successive time points in real BLT data exhibits similar deviations from equations (1), (S8) as we observed in our simulations. We reanalyzed data from three published BLT studies, which we refer to as the Levy 2015 [3], Li 2019 [26] and Venkataram 2023 [30]. We find strong deviations from linearity in the Li 2019 and Venkataram 2023 data at later time points (Figures 2 F,G). Even at the initial time point where we expect the linear relationship given by equation (1) to hold, we find that this linear relationship has a statistically significant positive $y$-intercept (2.26 for Levy 2015, 11.10 for Li 2019, and 7.32 for Venkataram 2023, all $P$-values $< 10^{-12}$, F-test; Figure 2E–G). As a result of these deviations from the expected behavior, the estimates of mean fitness and selection coefficients of individual adaptive mutations in real data are likely subject to the same biases as we observed in simulated data sets.

We sought to better understand why the observed relationship between $r_{k-1}$ and $\bar{r}_k$ deviates from the prediction given by equation (1). This relationship is expected to be non-linear if some lineages carry adaptive mutations. Indeed, after selection had time to act, lineages that carry stronger beneficial mutations are typically present at higher frequencies in the population and hence have higher values of $r_{k-1}$ than lineages than neutral lineages or those that carry weaker beneficial mutations. As a result, lineages with higher $r_{k-1}$ are also expected to increase disproportionately more or decrease disproportionately less than lineages with lower $r_{k-1}$, consistent with our observations (see Figure 2C,F,G).

The nature of a non-zero $y$-intercept in the relationship between $r_{k-1}$ and $\bar{r}_k$ is more subtle. To understand it, consider first those lineages that are represented by zero reads at time point $t_{k-1}$, i.e., those with $r_{k-1} = 0$. This set of lineages contains two subsets, those that are truly extinct as well as those that are present in the population but are not sampled by chance. Obviously, the expected read count at the next time point for the extinct lineages is zero. However, some lineages from the latter subset may survive to the next time point and be sampled then. Therefore, the expected frequency $\bar{r}_k$ for lineages with $r_{k-1} = 0$ is strictly positive. This is an instance of the more general regression to the mean phenomenon, whereby lineages whose read counts are by chance abnormally low at one time point will be represented by a more typical larger number of reads at the next time point, causing the $y$-intercept to be non-zero (see Supplementary Information, Section 4.3.2 for an extended discussion). The specific value of the intercept depends, among other things, on measurement noise and on the distribution of true lineage frequencies, quantities that are a priori unknown. Therefore, correcting for this offset appears difficult.

Taken together, our results demonstrate that the neutral decline approach is statistically problematic. Most importantly, it relies on the assumption that the measured barcode frequency is an unbiased estimator of the true lineage frequency in the population, which is in general incorrect. As a result, the accuracy of equation (1) or that of a more complex equation for the entire probability distribution of lineage reads depends on quantities that are a priori unknown, such as the true distribution of lineage sizes in the population. Therefore, the accuracy of mean fitness estimation can vary depending on which lineages are chosen as reference. How to choose reference lineages optimally is unclear because lineage abundance that maximizes accuracy depends on the strength of selection (which is a priori unknown) and changes over time.

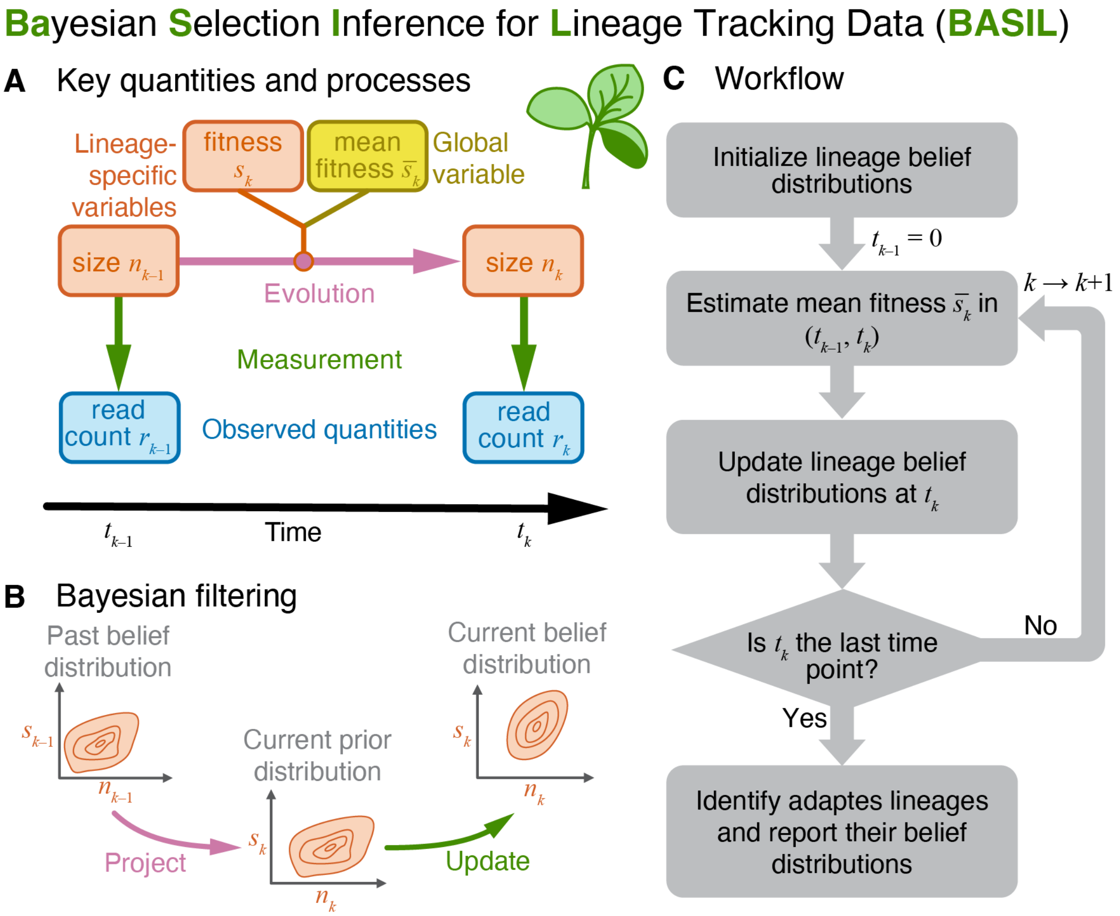

**Figure 3. BASIL schematic. A.** Key quantities and processes. Each barcoded lineage is characterized at the current time $t_k$ by its size $n_k$ and fitness (selection coefficient) $s_k$, which are unknown. Lineage's size changes over time depending on the lineage's fitness and the mean fitness of the population. At each sampling time point, we measure relative lineage sizes by counting sequencing reads with the corresponding barcode. **B.** Bayesian filtering consists of projecting the past belief distribution over the hidden variables $n_k$ and $s_k$ to obtain the prior distribution for the current time point and then updating this distribution using read count data to obtain the new belief distribution. **C.** BASIL workflow.

### BASIL: Bayesian selection inference for lineage tracking data

To overcome these challenges, we developed BASIL (Bayesian Selection Inference for Lineage tracking data), a robust statistical method for identifying adapted lineages and inferring their fitness. In developing BASIL, we first formulate a model for the unobserved evolutionary dynamics that unfold between two consecutive sampling time points. The key hidden variables in this model are the sizes and selection coefficients (fitness) of individual lineages as well as the mean fitness of the population and the measurement noise parameter, which are global variables. Our model of evolution relates the change in the size of each lineage to its selection coefficient and the population's mean fitness (Figure 3A). We also develop a model of measurement, which relates the true lineage size at a given time point to the corresponding barcode read count observed in the sequencing data. We then cast these models within the Bayesian filtering framework (Figure 3B, [41]). At any given time, each lineage is characterized by the joint belief distribution for its size and fitness. These belief distributions are based on the past data, i.e., data accumulated up to the current time point. We use these distributions to estimate the mean fitness of the population at the subsequent time interval, without relying on the existence of truly neutral reference lineages (Figure 3C). We then use our model of evolution to project lineage belief distributions forward in time and obtain prior distributions at the next time point. Finally, we update the belief distribution using the current read-count data and our measurement model.

We implemented BASIL in a software package [42]. In this section, we outline the main ideas and expressions behind our method, with more mathematical details available in the Supplementary Information Section 5.

### Model of evolution

Consider a barcoded lineage with fitness $s$ relative to the ancestor and suppose that this lineage is represented by $n_{k-1} = n(t_{k-1})$ individuals in the population at the previous sampling time $t_{k-1}$. On average, such lineage will expand if $s$ is larger than the population's current mean $\bar{s}$ and it will contract if $s < \bar{s}$. Demographic noise will also cause its size to randomly fluctuate around this expectation, such that the lineage size $n_k$ at the current sampling time $t_k$ is a random variable. To model it, we assume that our BLT population grows in the batch-culture regime [24] and sampling happens immediately prior to a dilution step. Then, during dilution, a fraction $1/D$ of the population is transferred into the fresh medium and the rest is discarded, where $D > 1$ is the dilution factor. Immediately after dilution, the size of the focal lineage becomes $\tilde{n}$, which we draw from the Poisson distribution with mean $n_{k-1}/D$. Then, we assume that the lineage grows or shrinks exponentially and deterministically for the batch-culture cycle duration $\Delta t_c$, such that by the end of the cycle its size becomes $n(t_{k-1} + \Delta t_c) = \tilde{n} D e^{(s-\bar{s})\Delta t_c}$. If the next sampling point is the end of this cycle, then $n_k = n(t_{k-1} + \Delta t_c)$. Otherwise, we repeat the dilution and growth phases until the sampling time $t_k$ is reached. Note that this model allows for the possibility of lineage extinction during dilution.

### Model of measurement

The actual lineage sizes are not observable. Instead, the population is sampled at time points $t_0$, $t_1$, … and sequenced at the barcode locus, which involves a series of processing steps [43]. Then the sequenced reads containing each barcode are counted. This measurement process can be described by a probability distribution $P^m(r|n)$ of observing $r$ reads for a barcode lineage with $n$ cells. The simplest model for this process is the Poisson distribution with mean $nR/N$, where $R$ is the read depth (i.e., the total number of reads obtained for the sample) and $N$ is the total population size at the sampling point. However, sequencing read counts are often overdispersed with respect to the Poisson distribution [39,44]. Previously, Levy et al modeled measurement noise with a distribution that allows for overdispersion and in which variance scales linearly with the mean, similar to the Poisson distribution [3]. Other studies modeled read counts with a negative binomial distribution which permits an arbitrary scaling between mean and variance [45–47].

To test which variance to mean scaling captures the measurement noise process more accurately, we performed a calibration experiment. We assembled a barcoded population of *Saccharomyces cerevisiae* with a total size of $1.6\times10^7$ individuals that consisted of 26 subpopulations present at five different frequencies (from $10^{-1}$ to $10^{-5}$) and sequenced it with 9-fold replication to an average depth of $2\times10^5$ reads per replicate (see Supplementary Information Section 2 for details).

As expected, we found that the mean of the barcode frequency estimated from sequencing data is very close to the input frequency of each lineage (Figure 4A). To test the extent of overdispersion, we plotted the variance of the measured read counts $\sigma_r^2$ against the mean $\langle r \rangle$. If the measurement process was adequately described by the Poisson distribution, we would expect to observe a linear relationship with slope 1. On the other hand, Levy et al model predicts linear scaling with a slope greater than 1 [3]. We find that the Poisson model fits our data quite well for read counts $\lesssim$100 but fails for more abundant barcodes (Figure 4B). Instead, the variance is better fit by a nonlinear convex function of the mean. We capture this nonlinearity with a model

$$\sigma_r^2 = \langle r \rangle + \epsilon \langle r \rangle^2, \qquad (2)$$

where $\epsilon$ is a measurement noise parameter that controls the degree of overdispersion, and we estimate $\epsilon = 2.4\times10^{-3}$ (Figure 4B, blue line, Table S2). Thus, we model the measurement process with a negative binomial (NB) distribution with mean $\langle r \rangle = nR/N$ and variance given by equation (2).

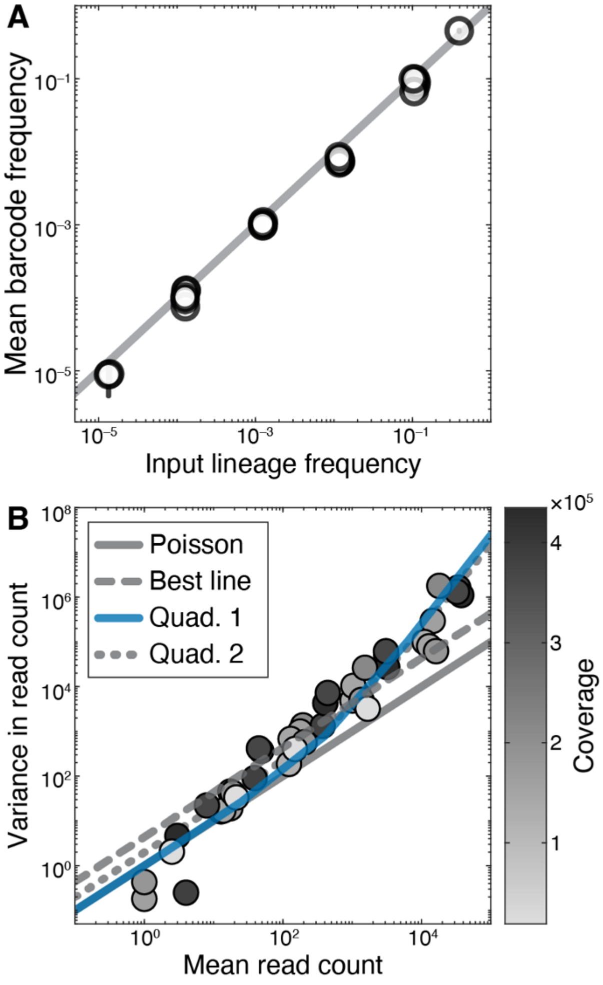

**Figure 4. Properties of the measurement process. A.** Barcode frequency estimated from barcode sequencing data (abscissa) versus the lineage frequency set experimentally (ordinate). Each point is a unique barcode. Error bars show one standard deviation of the mean. The 1-to-1 line is shown in gray. **B.** Variance of the read count versus the mean read count. Each point represents the data from multiple barcodes of the same lineage frequency. Shade represents coverage. Solid blue line is the parabola given by equation (2). Other curves are described in the Supplementary Information Section 2 and their fitter parameter values are provided in Table S2.

### Bayesian filtering

For each lineage $i$, we seek to estimate the joint belief distributions $P_{ik}^{b}(n,s)$ of lineage size $n$ and fitness effect $s$ at each sampling time $t_k$. First, assuming that the past belief distribution $P_{ik-1}^{b}(n,s)$ at $t_{k-1}$ and the mean fitness $\bar{s}_k$ in the time interval $(t_{k-1}, t_k)$ are known, we obtain the prior distribution $P_{ik}^{pr}(n,s;\bar{s}_k)$ at the current time $t_k$ by projecting the past belief distribution $P_{ik-1}^{b}$ using our model of evolution (see Supplementary Information Section 5.2.2 for mathematical details). The prior distribution $P_{ik}^{pr}$ represents our best guess for the abundance of lineage $i$ and its fitness at the current time point $t_k$, based on its past trajectory and the population's mean fitness. The observed read count $r_{ik}$ for this lineage may or may not be consistent with this guess, and we use this new data point to update our belief distribution (Figure 3B) using the Bayes' theorem.

$$P_{ik}^{b}(n,s) = \frac{1}{Z_{ik}^{S}} P^{m}(r_{ik}|n;\epsilon) P_{ik}^{pr}(n,s;\bar{s}_k) \text{ for } k \geq 1, \qquad (3)$$

where $P^m$ is the NB distribution defined in the "Model of measurement" section and $Z_{ik}^S$ is the marginal probability that $r_{ik}$ reads are observed at $t_k$, given all of our prior knowledge (subscript $S$ stands for "selection" and refers to the fact that this model allows for the fitness of the lineage to be non-zero). Note that $Z_{ik}^S$ depends on the noise parameter $\epsilon_k$ and the mean fitness $\bar{s}_k$, which are assumed to be known.

### Initialization

We initialize the belief distribution for any lineage $i$ as $P_{i0}^b(n, s) = \gamma_i(n)N(s)$, where $\gamma_i(n)$ is a Gamma distribution with parameters based on the lineage's initial read count and $N(s)$ is the normal distribution with zero mean and standard deviation 0.1 (see Supplementary Information Section 5.2.1 for details). In other words, we assume that all lineages are initially most likely neutral, but with large uncertainty.

### Estimating population's mean fitness

As mentioned above, our procedure for updating the belief distribution at time $t_k$ depends on the mean population fitness $\bar{s}_k$ and the noise parameter $\epsilon_k$ at the time interval $(t_{k-1}, t_k)$. We estimate $\bar{s}_k$ and $\epsilon_k$ based on the belief distributions $P_{ik-1}^b(n, s)$ up to and including time point $t_{k-1}$ (see Supplementary Information Section 5.3). Briefly, at each time interval $(t_k, t_{k+1})$, we randomly choose 3,000 lineages and classify them as either putatively adapted (set $A_k$) or putatively neutral (set $N_k$). A lineage is classified as putatively adapted if the mean of its belief distribution $P_{ik-1}^b(s)$ is positive and at least three times larger than its standard deviation; and it is classified as putatively neutral otherwise. We then estimate the mean fitness $\bar{s}_k$ and the noise parameter $\epsilon_k$ by maximizing the log-likelihood function

$$L_k(\bar{s}, \epsilon) = \sum_{i \in A_k} \log Z_{ik}^S(\bar{s}, \epsilon) + \sum_{i \in N_k} \log Z_{ik}^N(\bar{s}, \epsilon),$$

where $Z_{ik}^N$ is a quantity analogous to $Z_{ik}^S$ but for the neutral model (see Supplementary Information Section 5.3).

### Identification of adapted lineages

As a result of the procedure described above, for each lineage $i$, we obtain a time-varying belief distribution $P_{ik}^b(n, s)$, $k = 1, 2, \ldots$ We calculate the final belief distribution for the selection coefficient of lineage $i$ as $P_i^b(s) = \int_0^\infty P_{ik'(i)}^b(n, s)dn$ choosing the time point $t_{k'(i)}$ where the $s$-variance of the belief distribution $P_{ik}^b(n, s)$ is minimal. We call lineage $i$ adapted if the mean $\hat{s}_i$ of its final belief distribution $P_i^b(s)$ is sufficiently separated from 0, that is, if $\hat{s}_i \geq \beta\sigma_i$, where $\sigma_i$ is the standard deviation of $P_i^b(s)$. We refer to β as the "confidence factor", a hyper-parameter that must be determined empirically. Intuitively, larger β would require a lineage to have a higher fitness to be recognized as adapted, which would increase precision but reduce recall, or, equivalently, reduce the rate of false positives at the expense of increasing the rate of false negatives. In other words, a higher confidence factor would allow us to be more confident that the lineages that are identified as adapted are in fact adapted, albeit at the expense of missing more adapted lineages for which evidence of adaptation is weaker. On the other hand, decreasing β would increase recall but reduce precision, or, equivalently, it would allow us to identify more adapted lineages, albeit at a cost of also misidentifying more neutral lineages as adapted. Recall and precision usually exhibit a trade-off, and there is no general principle for choosing β. In the next section, we test our

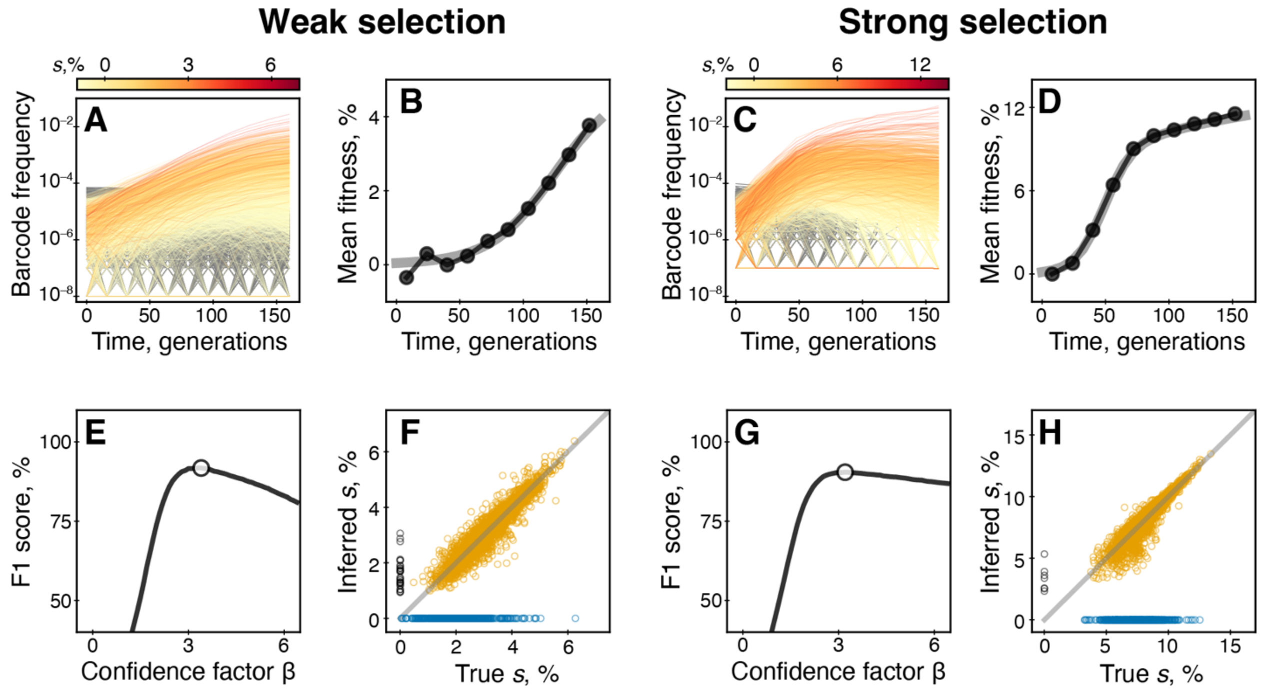

**Figure 5. Performance of BASIL on simulated data.** Left panels A, B, E, F show simulations in the weak selection regime; right panels C, D, G, H show simulations in the strong selection regime. **A, C.** Barcode frequency trajectories colored by fitness. **B, D.** Mean fitness trajectories. Grey lines show the true trajectory, black points show values inferred by BASIL. **E, G.** F1 score as a function of the confidence factor β. White circle denotes the maximal value. **F, H.** Inferred versus true selection coefficients of individual lineages. Lineages are called adapted with $\beta = 3.3$ (see main text). Each point corresponds to a lineage; orange points denote true positives, blue points denote false negatives and black points denote false positives, true negatives are not shown.

method on simulated data, which provides us with a guideline for the value of the confidence factor.

### BASIL performs well on simulated data

We assessed BASIL performance using simulated data in the weak and strong selection regimes described above (Figure 5). We found that BASIL accurately captures the dynamics of mean fitness in both regimes (Table 1; compare Figure 5B with Figure 1A,E and compare Figure 5D with Figure 1C,G), even at relatively late time points where the neutral decline approach often fails. To determine the hyper-parameter β, we varied it in the interval [0.0, 6.4] with step 0.1 and calculated F1 score for adapted lineage calls, which is the harmonic mean of recall and precision (Figure 5E,G). We found that the F1 score is maximized at $\beta = 3.4$ and $\beta = 3.2$ for the weak and strong selection regimes, respectively. Thus, we chose the value of $\beta = 3.3$ for all our subsequent analyses (see Supplementary Information, Section 6.1 and Figure S5). Using this confidence factor, we identified 2570 out of 3000 adapted lineages (430 false negatives) in the weak selection regime, with only 31 false positives, resulting in 98.8% precision and 85.7% recall, compared to 95.2% precision, 77.4% recall for the neutral decline method with low-abundance reference lineages (Table 1). In the strong selection regime, we identified 2481 out of 3000 adapted lineages (519 false negatives) with only 7 false positives, resulting in 99.7% precision and 82.7% recall, compared to 93.3% precision, 73.5% recall for the neutral decline method with low-abundance reference lineages. For lineages identified as adapted, our fitness estimates $\hat{s}_i$ are both accurate and precise in

| Measure of performance | Weak selection | | | Strong selection | | |
|---|---|---|---|---|---|---|
| | Neutral Decline | | BASIL | Neutral Decline | | BASIL |
| | LA Ref[1] | HA Ref[2] | $\beta = 3.3$ | LA Ref[1] | HA Ref[2] | $\beta = 3.3$ |
| TP[3] | 2204 | 2426 | 2570* | 1189 | 643 | 2481* |
| FP[4] | 37 | 49 | 31* | 1* | 1* | 7 |
| FN[5] | 796 | 574 | 430* | 1111 | 2357 | 519* |
| Precision[6], % | 98.3 | 98.0 | 98.8* | 99.9* | 99.8 | 99.7 |
| Recall[7], % | 73.5 | 80.9 | 85.7* | 63.0 | 21.4 | 82.7* |
| F1 score[8], % | 84.1 | 88.6 | 91.8* | 77.3 | 35.2 | 90.4* |
| Error in $\bar{s}$[9], % | 0.75 | 0.19 | 0.12* | 4.69 | 7.07 | 0.16* |
| Error in $\hat{s}$[10], % | 0.64 | 0.27 | 0.24* | 1.29 | 7.99 | 0.39* |

**Table 1. Comparison of BASIL with the neutral decline method.** [1]Low-abundance (LA) lineages used as reference; [2]High-abundance (HA) lineages used as reference; [3]Number of true positives; [4]Number of false positives; [5]Number of true positives; [6]Precision = TP/(TP+FP); [7]Recall = TP/(TP+FN); [8]F1 score = 2TP/(2TP+FP+FN); [9]Absolute difference between true and inferred, averaged across time; [10]Absolute difference between true and inferred fitness averaged over true positive lineages. *indicates the best performance.

both regimes, resulting in substantially smaller errors compared to the neutral decline method (see Table 1; compare Figure 5F with Figure 1B,F and compare Figure 5H with Figure 1D,H).

While the performance characteristics of our method are rather good and superior to those of the neutral decline approach, BASIL nevertheless fails to identify a significant proportion (between 15 and 20% in our simulations) of truly adapted lineages (Table 1). However, this is probably a biological inevitability rather than a flaw of the method itself. Some lineages, particularly those with weak adaptive mutations, may simply not reach large enough population sizes before they begin to decline due to clonal interference, which would prevent them from being identified as adapted by any method. Indeed, we find that false negative lineages in our simulations are typically only slightly more fit than the ancestor and their trajectories are essentially indistinguishable from those of truly neutral lineages, which supports our conjecture (see Supplementary Information Section 6.2 and Figures S6–S8 for more details). Thus, taken together, our results suggest that BASIL is a statistically robust, accurate and precise approach for the analysis of BLT data.

### Application of BASIL to data from BLT experiments

We next sought to assess how well BASIL performs on real BLT data. To this end, we selected five published BLT studies carried out with various strains of yeast *S. cerevisiae* propagated in different environmental conditions (see Table 2). Specifically, in BLT experiments described in Refs. [3,4,26], a haploid yeast strain that for simplicity we refer to as *HY0* adapted to four different environmental conditions that we refer to as C-1d, C-2d, C-5d, and N-2d. In conditions labelled as "C-" and "N-", yeast is limited by carbon and nitrogen, respectively, and the "-*x*d" notation refers to the number days *x* in the growth and dilution cycle. The BLT experiment described in Ref. [29] was carried out in the C-2d condition with three different haploid strains of *S. cerevisiae* that we refer to as *HY1, HY2* and *HY3* and that differ from *HY0* by one mutation each. Finally, the study in Ref. [30] evolved a diploid strain referred to as *DY*, which is a close relative of *HY0*, to two conditions that we refer to as N-5d and N-5d+*Cr*, where "+*Cr*" indicates the presence of the alga *Chlamydomonas reinhardtii*.

| Strain | Condition | Ref. | Replicates | Cycles[1] | Samples[1] | Dilution factor | Cells per lin.[2] | # lin., ×10[3] | Coverage[3] | Adapt. rate[4], % per gen | # adapt. lin[5]., ×10[3] | Mean $s_i$[5], % | Min $s_i$[5], % | Max $s_i$[5], % | $s_i$ IQR[5], % | $s_i$ IDR[5], % |
|---|---|---|---|---|---|---|---|---|---|---|---|---|---|---|---|---|
| *HY0* | C-2d | [3] | 1 | 14 | 12 | 250 | 142 | 490 | 158 | 0.04 | 61 | 1.9 | 0.4 | 11.5 | 1.2 | 2.1 |
| | | | 2 | 12 | 10 | 250 | 142 | 490 | 110 | 0.04 | 38 | 2.0 | 0.5 | 11.4 | 1.1 | 2.2 |
| | N-2d | [4] | 1 | 24 | 12 | 250 | 142 | 490 | 67 | 0.02 | 12 | 2.7 | 0.2 | 7.1 | 0.5 | 1.0 |
| | | | 2 | 25 | 12 | 250 | 142 | 490 | 71 | 0.04 | 27 | 3.2 | 0.6 | 6.9 | 0.7 | 1.2 |
| | C-1d | [26] | 1 | 25 | 10 | 128 | 4,090 | 17 | 87 | 0.09 | 3.3 | 8.5 | 2.7 | 41.5 | 3.4 | 5.9 |
| | | | 2 | 25 | 10 | 128 | 4,189 | 17 | 92 | 0.06 | 2.5 | 6.7 | 2.4 | 35.6 | 2.0 | 5.2 |
| | C-5d | [26] | 1 | 16 | 7 | 180 | 12,509 | 5.6 | 130 | 0.06 | 0.59 | 7.7 | 2.9 | 16.6 | 2.1 | 4.9 |
| | | | 2 | 16 | 7 | 180 | 8,871 | 7.9 | 71 | 0.02 | 1.5 | 5.7 | 2.0 | 12.9 | 2.4 | 4.7 |
| *HY1*[6] | C-2d | [29] | 1 | 20 | 12 | 256 | 130 | 620 | 12 | 0.02 | 40 | 1.4 | 0.4 | 7.2 | 0.6 | 1.8 |
| | | | 2 | 20 | 12 | 256 | 134 | 600 | 16 | 0.02 | 110 | 1.5 | 0.5 | 8.0 | 0.5 | 1.1 |
| *HY2*[6] | C-2d | [29] | 1 | 15 | 8 | 256 | 116 | 690 | 15 | 0.02 | 21 | 1.9 | 0.6 | 9.8 | 1.3 | 2.4 |
| | | | 2 | 20 | 11 | 256 | 122 | 650 | 13 | 0.03 | 39 | 1.5 | 0.5 | 8.5 | 0.7 | 1.7 |
| *HY3*[6] | C-2d | [29] | 1 | 20 | 11 | 256 | 146 | 550 | 10 | 0.04 | 55 | 2.3 | 0.7 | 10.7 | 1.3 | 2.8 |
| | | | 2 | 20 | 12 | 256 | 156 | 510 | 9 | 0.03 | 82 | 2.2 | 0.8 | 10.2 | 1.1 | 2.7 |
| *DY* | N-5d | [30] | 1 | 13 | 7 | 100 | 2.4 | 84 | 23 | 0.23 | 1.5 | 10.4 | 6.1 | 19.1 | 2.5 | 4.5 |
| | | | 2 | 13 | 7 | 100 | 2.4 | 84 | 26 | 0.21 | 1.4 | 11.1 | 6.2 | 19.4 | 2.9 | 5.3 |
| | | | 3 | 13 | 7 | 100 | 2.4 | 85 | 11 | 0.32 | 2.5 | 11.4 | 5.2 | 22.5 | 4.0 | 6.9 |
| | | | 4 | 13 | 7 | 100 | 1.9 | 100 | 18 | 0.25 | 2.4 | 10.9 | 5.1 | 22.2 | 3.7 | 6.5 |
| | | | 5 | 13 | 7 | 100 | 2.1 | 94 | 23 | 0.26 | 3.2 | 12.0 | 5.1 | 22.9 | 4.4 | 7.4 |
| | N-5d +Cr | [30] | 1 | 11 | 6 | 100 | 1.6 | 120 | 33 | 0.30 | 2.2 | 9.8 | 4.3 | 18.3 | 2.92 | 5.6 |
| | | | 2 | 11 | 6 | 100 | 1.5 | 130 | 36 | 0.35 | 2.0 | 10.0 | 5.4 | 18.1 | 3.0 | 5.7 |
| | | | 3 | 11 | 6 | 100 | 2 | 98 | 20 | 0.30 | 2.2 | 9.3 | 4.5 | 18.0 | 3.0 | 5.8 |
| | | | 4 | 11 | 6 | 100 | 2.1 | 97 | 19 | 0.22 | 2.2 | 9.3 | 4.4 | 17.3 | 3.1 | 5.9 |
| | | | 5 | 11 | 6 | 100 | 1.6 | 120 | 23 | 0.35 | 2.2 | 10.0 | 4.5 | 20.0 | 3.2 | 6.3 |

**Table 2. Characteristics of BLT datasets and BASIL performance on them.** [1]Number of cycles and sampling times used for inference (subsequent cycles/samples are ignored). [2]Expected number of cells per lineage at bottleneck at the beginning of the experiment. [3]Expected number of sequencing reads per barcode at the beginning of the experiment. [4]Average gain in main fitness, as inferred by BASIL. [5]Mean, minimum, maximum, interquartile range (IQR) and interdecile range (IDR) for lineage selection coefficients inferred by BASIL. [6]HY1, HY2 and HY3 refer to *cyr1*$^{S917Y}$, *gpb2*$^{Y282*}$, *tor1*$^{F1712L}$ mutants, respectively (see Ref. [29]).

We first examine the trajectories of mean fitness inferred by BASIL (Figure 6). We find that these trajectories are generally consistent with those calculated on the basis of identified adapted lineages (Figure S9). Mean fitness inferred by BASIL increases in all experiments at rates that are consistent across replicates (Table 2). These rates vary by about tenfold across conditions and strains, from about 0.02% per generation (*HY0* in N-2D condition) to about 0.3% per generation (*DY* in both N-5d and N-5d+*Cr* conditions). While BASIL is overall successful at inferring mean fitness dynamics in real populations under a wide range of selection regimes, we also observe some sub-optimal performance. In particular, we find that in the BLT experiments by Venkataram et al, the inferred mean fitness declines at late time points, something that we have not seen on our simulated data. Since there is a strong theoretical expectation that the mean fitness in large populations should not decline, this observation indicates that BASIL fails to accurately infer mean fitness at later time points in this data, likely because of the prevalence of secondary adaptive mutations [4,5]. This conjecture is supported by the fact that in the BLT experiments by Venkataram et al, adaptation occurs at the fastest rate, suggesting that secondary adaptive mutations arise in this experiment sooner than in other experiments. The fact that secondary mutations lead to declines in the inferred mean fitness can be mechanistically explained as follows. As lineages

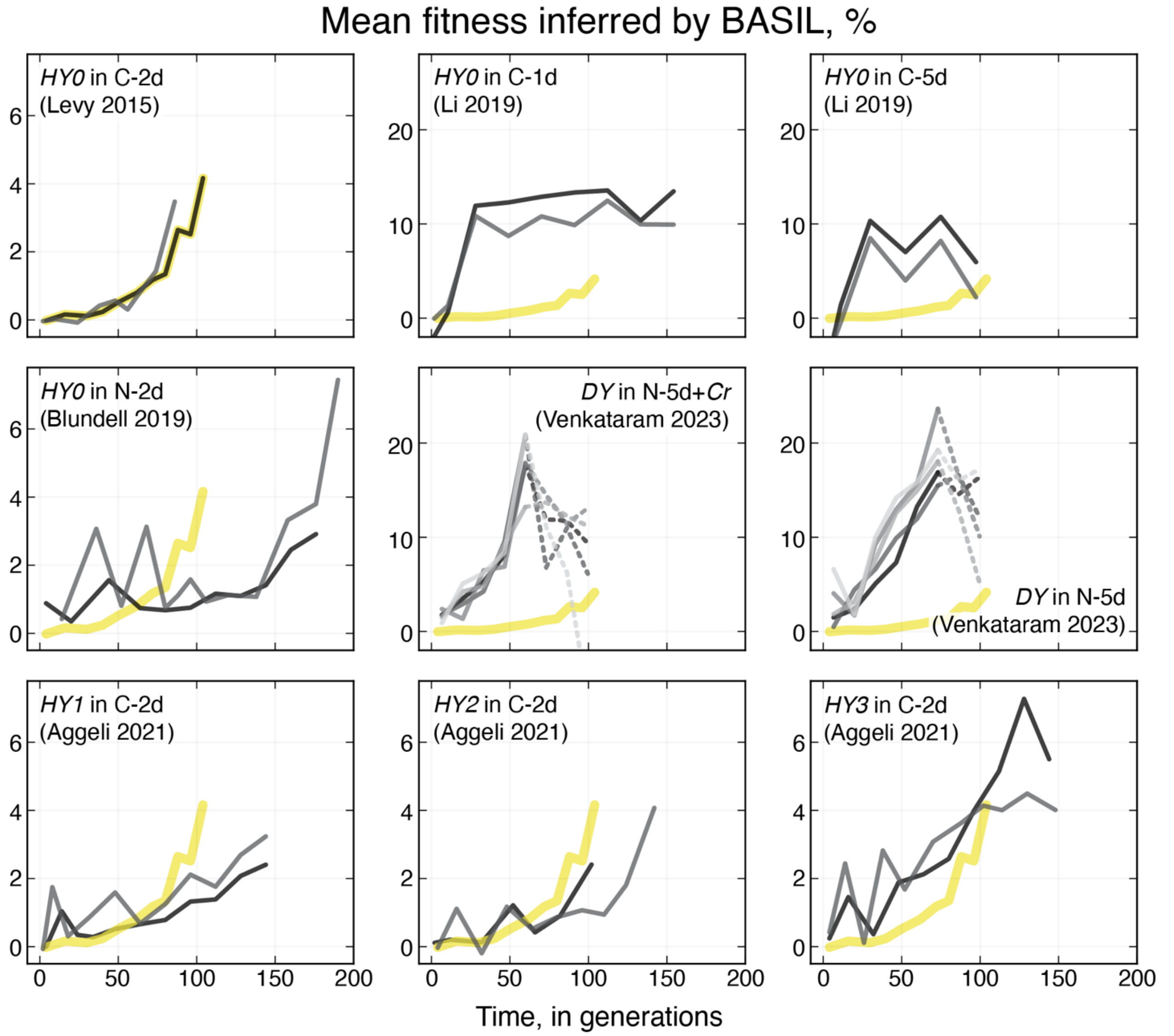

**Figure 6. Mean fitness inferred with BASIL in nine BLT experiments.** Each panel shows a BLT experiment, as indicated. Gray lines are different replicates; dashed lines indicate samples that are not used for the inference of adapted lineages. Thick yellow line shows the mean-fitness trajectory of *HY0* in C-2d (Levy 2015, Replicate 1) as reference.

acquire secondary adaptive mutations, their frequencies begin to rise faster than expected from previous observations. However, at later time points, BASIL has high confidence in the fitness estimates of most lineages. Thus, as it estimates the mean fitness at the next time interval, it attributes the unexpected frequency increases of multiple lineages to a decline in the mean fitness.

Next, we turn to the analysis of fitness effects of individual adaptive mutations. We find that the number of identified adapted lineages can vary substantially, by up to threefold, across replicate BLT experiments (Table 2). We also find that, while the measured distributions of fitness effects (mDFEs) look similar across replicate experiments (Figures 7, S10), they are statistically distinguishable by several measures (Tables 2, S5). This variation could arise from real biological variation, measurement noise, or both. Biologically, strong rare beneficial mutations could arise in different replicates at different times, causing real biological variation in the mean fitness trajectories (Figure 6), which would in turn lead to substantial variation in the probability of establish-

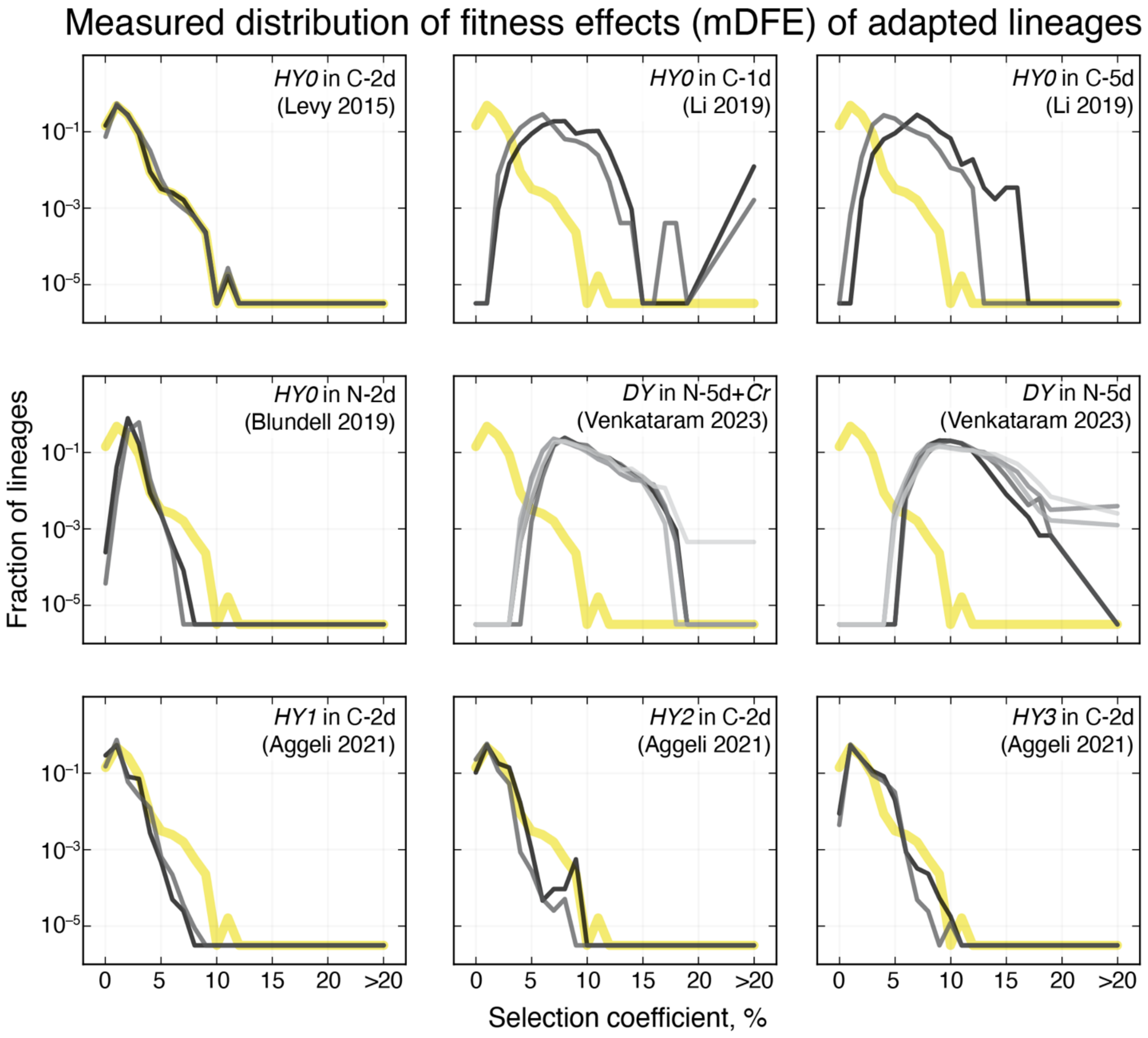

**Figure 7. Histograms representing the measured distributions of fitness effects (mDFEs) of adapted lineages inferred by BASIL in nine BLT experiments.** Notations are as in Figure 6.

ment of weaker beneficial mutations. In addition, the power to detect beneficial mutations, especially the weak ones, could depend on the experimental sampling protocol, such as coverage and the sampling effort (i.e., how frequently the population was sampled), which can also vary across replicates (Table 2). In particular, we expect that lower coverage and lower time resolution would reduce the number of called adapted lineage, which could affect the shape of the mDFE.

To test how sampling affects our inference, we down-sampled the data from the original BLT study in Ref. [3] (strain *HY0* in C-2d, Replicate 1) and reran the BASIL analysis (see Supplementary Information, Section 6.3). We find that when coverage is reduced from about 160x to about 10x per lineage on average, the inferred mean fitness trajectories remain virtually unchanged (Figure 8A) but the number of lineages identified as adapted decreases by 19% (from 60,920 to 49,330). 98.8% of these calls (48,738 out of 49,330) are the same as in the full dataset, and their inferred fitness effects are also consistent (95.8% Pearson correlation and 0.20% error in $s$, Figure 8B). The vast majority, 11,278/12,182 (92.6%) of calls missed in the down-sampled data are those with weak selection coefficients ($s < 2\%$; Figure 8C). As a result, the mean of the mDFE, its variance and other statistics change significantly between the full and reduced dataset (Table S6).

Similarly, when the number of sampling time points is reduced from 12 to 8, the number of called adapted lineages decreases by 21% (from 60,920 to 48,206). Over 99.9% of these calls (48,177 out of 48,206) are the same as in the full dataset and their inferred fitness effects are also consistent (97.9% Pearson correlation and 0.18% error in $s$, Figure 8E). While the majority 12,607/12,743 (98.9%) of missed adapted lineages are those with selection coefficients less than 2% (Figure 8D), we find that reduced sampling effort leads to a substantial depletion of identified adapted lineages even with strong fitness benefits (Figure 8F).

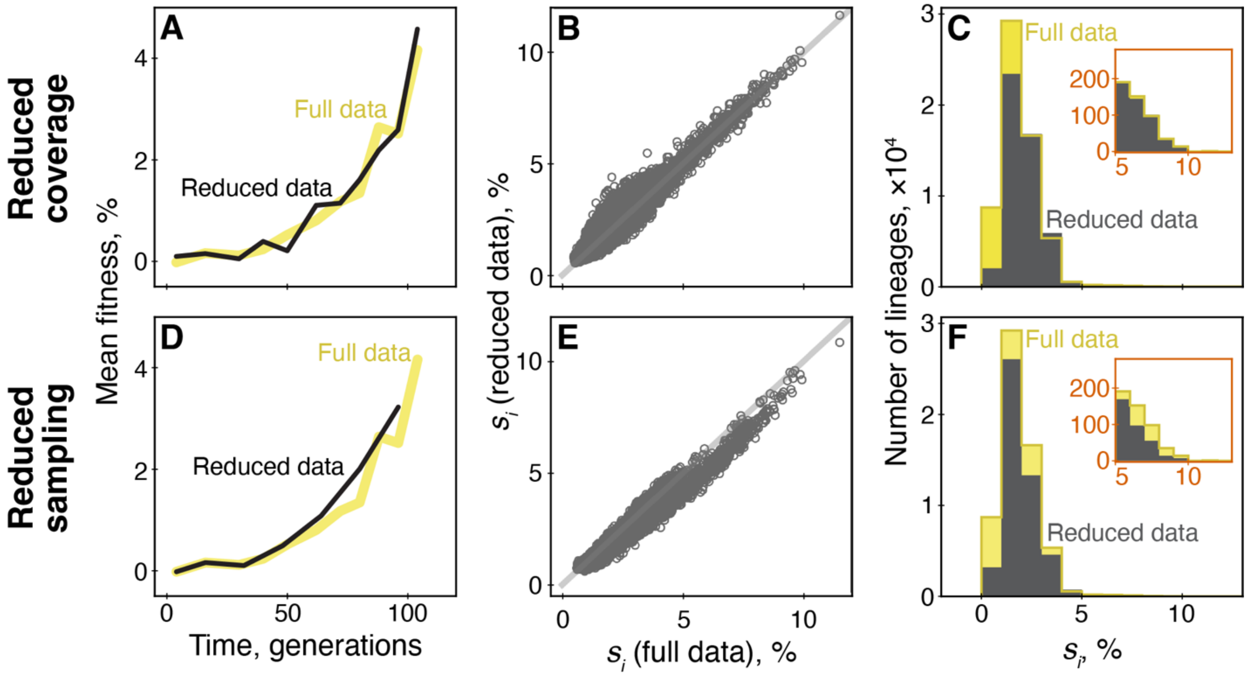

**Figure 8. The effects of down-sampling on BASIL performance.** We reanalyzed the Levy 2015 dataset (*HY0* in C-2d, Replicate 1) after reducing sequencing coverage (panels A, B, C) or sampling effort (panels D, E, F). See text and Supplementary Information for details. **A, D.** Inferred mean fitness trajectories. **B, E.** Inferred lineage selection coefficients for lineages identified as adapted in both full and reduced datasets. The 1-to-1 line is shown in light gray. **C, F.** Distribution of fitness effects of identified adapted lineages. Insets show tails of the distributions.

Taken together, these results show that when BASIL is applied to real BLT data it produces reliable estimates of the mean fitness trajectories during the initial stages of the BLT experiment (prior to the point where double mutants begin to dominate), robustly identifies adapted lineages and infers their selection coefficients. However, the statistics of the mDFE are highly sensitive to sources of biological and measurement noise, which could pose challenges for inferring the properties of the true underlying DFE from these measurements. Specifically, our results suggest that reduction in sampling frequency is more detrimental for inference than a uniform reduction in coverage across all time points.

### DISCUSSION

In this work, we developed BASIL, a Bayesian filtering method for identifying barcoded lineages that carry nascent adaptive mutations and for the inferring their selection coefficients. BASIL estimates belief distributions for these selection coefficients and the corresponding lineage sizes, updating them over time based on the observed barcode read counts. Measurement noise is a key feature of our model, and our new experimental data suggests that the measurement variance in the read count scales quadratically with the expected read count, as opposed to a linear scaling that has been postulated in previous studies. One notable advantage of our framework is that it overcomes the challenges associated with estimating mean fitness based on reference lineages that need to be designated a priori. This makes our approach applicable to a wide range of evolutionary scenarios, including those with strong selection.

When we applied BASIL to real BLT data, we found that while the inferred mean-fitness trajectories are generally similar across replicate experiments, the number of identified adapted lineages and the statistics of their inferred fitness distributions are not. Our analysis of down-sampled data showed that the power to call adapted lineages, particularly those carrying weakly beneficial mutations, is quite sensitive to the sampling protocol, with sampling frequency being more important than read depth.

BASIL computes a time-dependent joint belief distribution for the selection coefficient and size of every lineage. So far, we did not utilize all the information contained in this distribution, but instead chose to classify lineages into adapted and neutral with a simple linear classifier that is based on a snap-shot of this distribution. In our simulations, this simple classifier with the confidence factor of 3.3 (which controls the slope of the classification line) was very effective. How effective it is on real data, which is arguably more complex than our simulations, is difficult to ascertain. We visualize our classification on two-dimensional diagnostic plots where the mean of the fitness belief distribution is plotted against its standard deviation (Figures S5,S11). In some datasets, these plots reveal clustering of lineages, with some clusters clearly containing highly adapted lineages (Figure S11), similar to our simulations (Figure S5). It is possible to isolate these highly adapted clusters by adjusting the confidence factor, which can be easily done by the user in the current implementation of BASIL. However, while such an adjustment would likely improve precision, it may significantly worsen recall, since not all adapted lineages belong to the highly adapted clusters, as we saw in our simulations (Figure S5). Instead, developing more sophisticated classifiers and testing them on more realistic simulations could be a valuable direction for future research.

Our approach has certain limitations. On the technical side, BASIL is quite computationally expensive since it requires extensive Markov Chain Monte Carlo (MCMC) sampling to estimate posterior distributions of lineage fitness and sizes. For instance, the analysis of a BLT experiment with $5\times10^5$ lineages sampled at 10 time points took us ~50 hours of running time on AMD Ryzen 5 7600X 6-Core Processor, and the running time increases linearly in the number of lineages and sampling time points. However, since MCMC computations for different lineages are independent, the algorithm is easily parallelizable. In our analyses, we found it convenient to use 12–32 processor cores.

One important conceptual limitation of our model is that it assigns a single constant selection coefficient to each barcoded lineage, but many barcoded sub-populations are in fact polymorphic. Indeed, each new adaptive mutation arises in a single cell, and the mutant lineage initially constitutes only a small fraction of the parental barcoded sub-population [3]. The mutant lineage outcompetes the wildtype within this sub-population gradually, such that the barcode frequency increases slower than would be expected from the mutant's selection coefficient alone. Since BASIL ignores these intra-lineages dynamics, it probably underestimates the selection coefficient of lineages that arise later in the BLT experiment and also undercounts them. However, the magnitude of this bias is hard to predict a priori because it depends on the rate of adaptive mutations and their fitness effects, the very quantities that we are trying to estimate.

A barcoded sub-population also becomes polymorphic when secondary adaptive mutations arise and begin to establish [5]. In principle, BASIL is capable of updating the belief distribution for any given lineage if its fitness changes, but in practice, convergence to a stable new distribution is probably slow at later time points when the confidence in the current belief is already high. As a result, when secondary mutations become dominant, BASIL breaks down. Thus, BASIL can currently be reliably applied only to short-term BLT experiments and does not accommodate fluctuating or frequency-dependent selection or adaptation in spatially structured populations where expansion of adapted lineages is sub-exponential [48]. However, it should be possible to extend our approach to these more general cases.

It is important to keep in mind that BASIL only identifies adapted lineages and infers their selection coefficients but does not infer the rate at which beneficial mutations arise. To infer the latter, we would need to know not only how many beneficial mutations were detected but also how many arose but were lost by drift while rare and how many were established but not detected. In other words, we would need a model of mutant's evolutionary dynamics as well as a model of sampling and detection. Previously, Levy et al developed the former model and inferred the rates of adaptive mutations with different fitness effects [3], but their model did not account for biases introduced by sampling and detection. We have shown that these biases are not negligible. Therefore, incorporating them into a model of beneficial mutation rate inference is an important problem for future research.

### ACKNOWLEDGEMENTS

We thank members of the Kryazhimskiy lab for discussions and feedback. The UC San Diego Triton Computing Cluster assisted with computational work. This work was supported by the Career Award at Scientific Interface (1010719.01) from the Burroughs Wellcome Fund to SK, the Alfred P. Sloan Research Fellowship (FG-2017-9227) to SK, the Hellman Fellowship to SK, and the NIH grants 1R01GM137112 and R35GM153242 to SK.

### AUTHOR CONTRIBUTIONS

Conceptualization: HK, SK; Experimental design: HK, SK; Experiments: HK; Analysis: HK, SK; Writing: HK, SK.

## COMPETING INTERESTS

None

## SUPPLEMENTARY MATERIALS

### Supplementary Text

### Supplementary Figures

Figure S1. Simulated BLT data.

Figure S2. Consistency of mean-fitness inference for BLT data by Levy et al.

Figure S3. Parametric fit to the MCMC-sampled belief distributions.

Figure S4. Dynamics of the belief distribution.

Figure S5. Discrimination of adapted and neutral lineages in simulated data.

Figure S6. The frequency dynamics of lineages in the weak-selection regime, stratified by class label.

Figure S7. The frequency dynamics of lineages in the strong-selection regime, stratified by class label.

Figure S8. Selection coefficients and sizes of lineages identified as adapted in simulated data.

Figure S9. Mean fitness trajectories inferred by BASIL in real BLT datasets compared to mean fitness trajectories calculated from adapted lineages.

Figure S10. Quantile-quantile plots for the distributions of selection coefficients of lineages identified as adapted in replicate BLT experiments.

Figure S11. Discrimination of adapted and neutral lineages in real BLT data.

### Supplementary Tables

Table S1. Fitted parameters for models of the relationship between read-count mean and variance.

Table S2. Statistical comparison of models of the relationship between read-count mean and variance.

Table S3. Simulation parameters.

Table S4. Mathematical notations used in BASIL.

Table S5. Differences of mDFE statistics across replicates in real BLT datasets.

Table S6. Effects of down-sampling on the characteristics of mDFEs for the Levy 2015 dataset.

### Supplementary Data

Data S1 [Excel]. Aggregated Data Tables.

Tab 1. Measurement noise experimental data.

Tabs 2–10. BASIL results for published BLT experiments.

Tabs 11,12. BASIL results for the down-sampled Levy et al data.

### Raw Data Repository https://dataview.ncbi.nlm.nih.gov/object/PRJNA1227420

### Code https://github.com/HuanyuKuo/BASIL-public

## Supplementary Materials for

## A Bayesian Filtering Method for Estimating the Fitness Effects of Nascent Adaptive Mutations

Huan-Yu Kuo, Sergey Kryazhimskiy*

*E-mail: skryazhi@ucsd.edu

### Contents

**1 Code and data availability** **3**
1.1 Code . . . 3
1.2 Data . . . 3

**2 Estimation of noise in the barcode frequency measurement process** **3**
2.1 Strains . . . 3
2.2 Culture preparation . . . 3
2.3 Barcode sequencing . . . 4
2.4 Data analysis . . . 4

**3 BLT simulations** **7**

**4 The neutral decline method** **8**
4.1 Review of the Levy-Blundell method . . . 8
4.2 The neutral decline method . . . 10
4.3 Key assumptions of the Levy-Blundell method . . . 11
4.3.1 Assumption 1. Low-frequency lineages are neutral . . . 11
4.3.2 Assumption 2. Read frequency dynamics faithfully represent lineage frequency dynamics . . . 12
4.4 Empirical test of the neutral decline equation . . . 15

**5 BASIL** **16**
5.1 Model of measurement . . . 16
5.2 The belief distribution for lineage size and fitness . . . 17

5.2.1 Initialization . . . . . . . . . . . . . . . . . . . . . . . . . . . 18
5.2.2 Projecting the belief distribution . . . . . . . . . . . . . . . . . 18
5.2.3 Updating the belief distribution . . . . . . . . . . . . . . . . . . 21
5.3 Estimating population's mean fitness and the noise parameter . . . . 24
5.4 Identification of adapted lineages and estimation of their selection coefficients . . . . . . . . . . . . . . . . . . . . . . . . . . . . . . . 27
5.5 Implementation . . . . . . . . . . . . . . . . . . . . . . . . . . . . 28

**6 Application of BASIL to simulated and real BLT data 29**
6.1 Belief distribution and lineage identification . . . . . . . . . . . . . 29
6.2 Performance on simulated data . . . . . . . . . . . . . . . . . . . . 29
6.3 Performance on experimental data . . . . . . . . . . . . . . . . . . . 31

**A Expectation of the noise variance estimator and bias correction 33**

**B Expected frequency given the number of reads 34**

**C Expectation of the logarithm of a gamma-distributed random variable 36**

**7 Supplementary Figures 37**

**8 Supplementary Tables 49**

## 1 Code and data availability

### 1.1 Code

All analysis code is written in Python and is available at https://github.com/HuanyuKuo/BASIL-public

Computationally intensive analyses were run on the Triton Supercomputing Cluster (TSCC).

### 1.2 Data

Barcode sequencing data generated in this work (see Section 2) are available at https://dataview.ncbi.nlm.nih.gov/object/PRJNA1227420

## 2 Estimation of noise in the barcode frequency measurement process

### 2.1 Strains

26 clones were isolated from the barcoded library of the diploid strain GSY6699 of yeast *Saccharomyces cerevisiae* used in our previous work [1]. Details of the strain and library construction are provided in Refs. [2, 3]. Briefly, each clone carries a unique 30-bp DNA barcode that replaces one copy of the YBR209W locus; all clones are otherwise identical in the rest of the genome. Barcode sequences of all clones have been determined previously [1] and are reported in Supplementary Data S1 (Tab 1).

### 2.2 Culture preparation

We started a liquid culture of each clone from frozen stocks in 3 ml of YPD (10 g yeast extract, 20 g peptone, 20 g dextrose, 1 L Milli-Q water) in 16-mm test tubes, and incubated these cultures overnight, in a shaking incubator set at 200 rpm at 30°C. After overnight growth, culture densities were measured with a Coulter Counter and were found to be around $1.5 \times 10^8$ cell/ml. The measured values for all cultures are provided in Supplementary Data S1 (Tab 1).

To probe the measurement noise process, we aimed to construct a barcoded clone mixture consisting of 5 clones at each of the frequencies 0.1, 0.01, $10^{-3}$, $10^{-4}$, $10^{-5}$, and one clone at the remaining frequency. For clones with the four lowest target frequencies (those below and including 0.01), we diluted the overnight cultures $1:10$, $1:100$, $1:10^3$ and $1:10^4$ in PBS, respectively. To make the final clone mixture, we pooled 200 µl of each diluted culture, then added 200 µl of the five cultures with target frequency 0.1, and then added 600 µl of the culture with the largest frequency. This resulted in final mixture volume of 5.6 ml at density approximately $4.6 \times 10^7$ cell/ml with barcodes clustered around six distinct frequencies: 0.395, 0.108, 0.019, $1.24 \times 10^{-3}$, $1.28 \times 10^{-4}$ and $1.41 \times 10^{-5}$. Our estimates of clone frequencies in the mixture are provided in Supplementary Data S1 (Tab 1). We spun down the clone mixture, re-suspended it in 20 ml of PBS (final density $1.57 \times 10^7$ cell/ml), split it into 20 aliquots (1 ml each in a 2 ml cryogenic tube), supplemented with 500 µl of 80% glycerol, and stored at $-70$°C.

### 2.3 Barcode sequencing

We followed the same protocols for DNA extraction and sequencing library preparation as in our previous work [1]. We repeated this procedure 10 times, each time using one aliquot of the barcoded clone mixture stocks described above. All ten replicate libraries were sequenced on the Illumina HiSeq platform, resulting in approximately $2.3 \times 10^5$ total reads.

### 2.4 Data analysis

We used BarcodeCounter2 to identify barcodes in the sequencing data and count them [4]. The data are shown Supplementary Data S1 (Tab 1). One replicate was excluded from analysis due to low coverage. In addition, we could not identify barcodes for two out of 26 clones, and two other clones (in the lowest frequency class) were not detected in the sequencing data. These four barcodes were removed from further analysis. Thus, we retained 22 barcodes present in all $n_{\text{rep}} = 9$ replicates.

**Estimation of measurement noise variance.** Denote by $X_p$ the set of barcodes with the same target frequency $p$. Variation in the number of reads $r_{bi}$ across barcodes $b \in X_p$ and replicates $i$ arises from three sources. First, the same barcode is represented by different numbers of reads in different replicates since replicates receive different total coverage. Specifically, if the barcode's true frequency in the pool is $p_b$, we expect to obtain $p_b R_i$ reads for this barcode in replicate $i$ with total coverage $R_i$. Second, the actual number of reads obtained for barcode $b$ will deviate from $p_b R_i$ by some random amount $\delta r_{bi}$ due to noise during library preparation and sequencing [5]. We refer to $\delta r_{bi}$ as "measurement noise". Finally, while we aimed to seed the barcoded clones within the same frequency class $X_p$ with exactly the same target frequency $p$, in reality, the frequency $p_b$ of barcode in the pool actually deviates from $p$ by some amount $\delta p_b$, i.e., $p_b = p + \delta p_b$. We refer to $\delta p_b$ as the "experimental noise". Putting all of this together, we can model the read count $r_{bi}$ for barcode $b \in X_p$ as

$$r_{bi} = p_b R_i + \delta r_{bi}, \tag{S1}$$

where $p_b$ is unknown and we assume that all $\delta r_{bi}$ are independent random variables with zero mean, and all $\delta r_{bi}$ with the same $b$ are identically distributed with variance $\sigma^2_{pi}$ (the noise variance) where index $p$ indicates that noise variance can vary between frequency classes $X_p$, and index $i$ indicates that it can also vary between replicates $i$. Our goal is to estimate $\sigma^2_{pi}$.

The challenge with estimating $\sigma^2_{pi}$ is that each barcode has its an unknown expected number of reads $p_b R_i$ in each replicate $i$. If all barcodes had exactly the same frequency $p$, then we could use $r_{bi}$ from different barcodes in the same replicate to estimate $p_b R_i$ as

$$\bar{r}_{pi} = \frac{1}{|X_p|} \sum_{b \in X_p} r_{bi}. \tag{S2}$$

However, different barcodes $b \in X_p$ in fact have different frequencies $p_b$, and the estimate (S2), which ignores this variation, would provide a poor estimate for the expected barcode count $p_b R_i$ and risks inflating our estimate of the measurement noise. To overcome this problem, we first estimate $p_b$ as

$$\hat{p}_b = \frac{1}{n_{\text{rep}}} \sum_{j=1}^{n_{\text{rep}}} \frac{r_{bj}}{R_j}.$$

Then, we estimate the expected number of reads $\bar{r}_{bi}$ for barcode $b$ in replicate $i$ as

$$\bar{r}_{bi} = \hat{p}_b \, R_i = \frac{R_i}{n_{\text{rep}}} \sum_{j=1}^{n_{\text{rep}}} \frac{r_{bj}}{R_j}. \tag{S3}$$

We can now use expectation (S3) to estimate the variance in the read count due to measurement noise in frequency class $X_p$ and replicate $i$ as

$$\hat{\sigma}_{pi} = \frac{1}{|X_p|} \sum_{b \in X_p} \left(r_{bi} - \bar{r}_{bi}\right)^2. \tag{S4}$$

In Appendix A, we derive the expectation for this estimator. We find that this estimator is biased, approximately by a factor $1 - 1/n_{\text{rep}}$ and derive a bias-corrected estimate (see equation (S68)). However, this corrected estimate is not guaranteed to be non-negative, and indeed we found that the variance estimate for one of the replicates in the lowest frequency class becomes negative upon applying this correction. We also found that for 36 out of 41 (88%) values of $\hat{\sigma}_{pi}$ that we could estimate, the correction changes the estimated value by less than 25%. For these reasons, we decided to use the uncorrected estimate given by equation (S4) for the subsequent analysis.

**Relationship between read count mean and variance.** Figure 4B in the main text shows the variance $\hat{\sigma}^2_{pi}$ calculated using equation (S4) plotted against the mean barcode read count $\bar{r}_{pi}$ within the frequency class $X_p$ in replicate $i$, calculated using equation (S2). We model this relationship as

$$\hat{\sigma}^2_{pi} = a\,\bar{r}_{pi} + \epsilon\,\bar{r}^2_{pi}, \tag{S5}$$

and test four special cases of this equation (Table S1). The simplest model of noise postulates that, if we obtain $R$ total reads for a given sample, the number of reads $r$ supporting a barcode whose frequency in the sample is $p$ will be distributed binomially with parameters $p$ and $R$. Given that $R$ is typically very large, the binomial distribution is well approximated by the Poisson distribution, for which the mean and variance are equal. To recapitulate the Poisson distribution, we set $a = 1$ and $\epsilon = 0$ in equation (S5). The noise model proposed by Levy et al (see equation (S7) below) allows for overdispersion relative to the Poisson distribution. In their model, which we refer to as "Linear general", the variance scales linearly with the mean but with a slope $a > 1$. Thus, we test whether the relationship between read count mean and variance is captured by equation (S5) with $\epsilon = 0$ and coefficient $a$ being a fitting parameter. To capture the possibility that the degree of overdispersion varies across read depths, we test a quadratic relationship with $a = 1$ and a fitting parameter $\epsilon > 0$, which converges to the Poisson model for small $\bar{r}_{pi}$. We refer to this model as "Quadratic with 1 parameter". Finally, we also fit the general quadratic model with two fitting parameters $a$ and $\epsilon$.

We find the best-fit parameters using the non-linear least squares method. Since the estimated measurement noise variances span six orders of magnitude (see Figure 4 in the main text), we carry out the fitting in the log space, i.e., we minimize the squared error between $\log\hat{\sigma}_{pi}$ and $\log\left(a\,\bar{r}_{pi} + \epsilon\,\bar{r}^2_{pi}\right)$. In Table S1, we report the best-fit parameters, residual sums of squares (RSS), as well as the coefficient of determination $R^2$, computed as $1 - \text{RSS}/\text{TSS}$, where TSS denotes the total sum of squares.

We compare the goodness-of-fit for all pairs of nested models using the $F$-test. Specifically, assuming that the null model has $p_{\text{null}}$ free parameters and the alternative model has $p_{\text{alt}}$ parameters ($p_{\text{null}} < p_{\text{alt}}$), we calculate the $F$-statistic as

$$F = \frac{\text{RSS}_{\text{null}} - \text{RSS}_{\text{alt}}}{\text{RSS}_{\text{alt}}}\,\frac{n - p_{\text{alt}}}{p_{\text{alt}} - p_{\text{null}}},$$

where $n$ denotes the number of data points, and $\mathrm{RSS}_{\mathrm{null}}$ and $\mathrm{RSS}_{\mathrm{alt}}$ are the residual sums of squares for the null and alternative model, respectively. If the data is generated by the null model, then the $F$-statistic is distributed according to the $F$-distribution with $(p_{\mathrm{alt}} - p_{\mathrm{null}}, n - p_{\mathrm{alt}})$ degrees of freedom.

The results of this analysis are shown in Figure 4B in the main text, and Tables S1 and S2. We find that the quadratic models explain at least 93% of variance in our data compared to linear models, which explain 89% or less. We also find that the general quadratic model provides only a marginally better fit than the 1-parameter quadratic model. Therefore, we use the 1-parameter quadratic model as our model for the relationship between read count mean and variance.

## 3 BLT simulations

As mentioned in the main text, we carry our simulations in the weak and strong selection regimes. We chose the parameters for the weak selection regime to make it similar to the experimental conditions described by Levy et al [2]. In particular, selection and genetic drift are relatively weak, and the variance in the read count is a linear function of the mean, as in Ref. [2]. For the strong selection regime, we chose parameters that should make inference harder. In particular, selection and drift are stronger, and the variance in the read count is a quadratic function of the mean (see equation (2) in the main text and Section 2.4). Simulation parameters for both regimes are provided in Table S3.

All simulations start with the same number $N_L = 10^5$ of barcoded lineages. The initial size of each lineage is drawn from an exponential distribution with mean $\langle N \rangle / N_L$, where $\langle N \rangle$ is the expected total population size (see Table S3). In each simulation, 3,000 randomly chosen lineages are adapted, such that all members of such lineages have the same beneficial mutation. We draw these lineages uniformly with the constraint that their initial size must exceed $1.5D = 384$ individuals, where $D = 256$ is the dilution factor. The selection coefficient (fitness) $s_i$ of each adapted lineage is drawn from the normal distribution with mean $\langle s \rangle$ and standard deviation $\sigma_s$, and draws with negative $s$ are discarded (see Table S3). The fitness of all other lineages are set to zero. No new mutations arise during the simulation.

After initializing our populations, we simulate their growth and dilution, with each growth-dilution cycle consisting of the following steps:

1. Each cycle begins with the calculation of the population's mean fitness $\bar{s} = \sum_i s_i n_i / N$, where $n_i$ is the current size of lineage $i$ and $N = \sum_i n_i$ is the current total population size.

2. We then simulate the dilution process. For each lineage $i$, we draw its new size $n'_i$ as a random number from the Poisson distribution with mean $n_i / D$. Thus, after dilution, the population's bottleneck size equals $N_b = \sum_i n'_i \approx \langle N \rangle / D$.

3. We then simulate population expansion. For each lineage $i$, we first calculate its growth factor
$$W_i = D \exp\{(s_i - \bar{s})\, \Delta t\}$$
where $\Delta t = 8$ generations, the duration of the growth phase. Thus, at the end of the growth phase, the new lineage size is $n_i = n'_i W_i$ (rounded to the nearest integer) and the total population size is again
$$\sum_i n_i \approx D \sum_i n'_i \,(1 + (s_i - \bar{s})\Delta t) = \langle N \rangle + \Delta t D \sum_i n'_i (s_i - \bar{s}) \approx \langle N \rangle \,.$$

Each simulation lasts for a total 20 growth-dilution cycles (160 generations). We simulate sampling and barcode sequencing by selecting $R$ random individuals from the population at the end of every other even cycle (i.e., before dilution). For each sampled barcode $i$, the number of sequencing reads $r_i$ is drawn from the negative binomial distribution with mean $\langle r_i \rangle = n_i / N\, R$ and variance $\sigma_r^2$, where $N = \sum_i n_i$ is the current number of cells in the population and $R$ is the sequencing depth (Table S3). The output of each simulation is a table of "sequenced" barcode counts at all sampling time points.

## 4 The neutral decline method

Levy et al developed a computational method for the analysis of barcode lineage tracking (BLT) data [2]. In this section, we briefly review their original method, which we refer to as the Levy-Blundell method. We then describe its simplified version, which we refer to as the "neutral decline" method, and which use for the analyses in this paper.

### 4.1 Review of the Levy-Blundell method

The Levy-Blundell method is based on a stochastic birth-death process that describes a mutant lineage starting from $n_0$ founding cells that randomly divide and die with certain per capita birth rate $b$ and death rate $d$. Levy et al derived the full stochastic expression for the probability $P_t(n)$ that the number of cells in the mutant lineage after time $t$ is $n$ (see equation (126) in the Supplementary Information to Ref. [2]). They then approximate this discrete distribution for large $n$ by a continuous probability distribution density

$$p_t(n) = \sqrt{\frac{\mu_t^{1/2}}{4\pi\, \kappa_t\, n^{3/2}}} \exp\left[-\frac{(\sqrt{n} - \sqrt{\mu_t})^2}{\kappa_t}\right], \tag{S6}$$

which they then use for the analysis of their data (see equation (127) in the Supplementary Information to Ref. [2]). Equation (S6) depends on two parameters: the mean of lineage size $\mu_t = n_0 e^{\lambda t}$ and the variance to mean ratio $\kappa_t = \frac{1}{2\lambda}(e^{\lambda t} - 1)$, where $\lambda = b - d$ is the mutant growth rate. One important difference between the full solution of the birth-death process and the approximation (S6) is that the full probability distribution has a non-zero weight at $n = 0$ (i.e., extinction is possible) whereas in the approximation (S6) the probability of extinction is zero.

The Levy-Blundell method then uses the same general form of equation (S6) to describe the dynamics of barcode read counts, that is, if a lineage with selection coefficient $s$ is observed to have $r_{k-1}$ read counts at the previous cycle $k-1$, then the probability density $p(r)$ that $r$ counts will be observed at the next cycle is assumed to be

$$p(r; \mu_k, \kappa_k) = \sqrt{\frac{\mu_k^{1/2}}{4\pi\, \kappa_k\, r^{3/2}}} \exp\left[-\frac{(\sqrt{r} - \sqrt{\mu_k})^2}{\kappa_k}\right], \tag{S7}$$

where

$$\mu_k(s, \bar{s}_k) = \frac{R_k}{R_{k-1}}\, r_{k-1}\, e^{(s-\bar{s}_k)(t_k - t_{k-1})}, \tag{S8}$$

is the expected read count. Here $R_{k-1}$ and $R_k$ are the total read depths at sampling times $t_{k-1}$ and $t_k$ respectively and $\bar{s}_k$ is the mean fitness of the population in the interval $(t_{k-1}, t_k)$. Unlike in the birth-death process, the variance parameter $\kappa_k$, which now characterizes all the noise in the BLT experiments including growth, dilution, DNA extraction, DNA amplification, etc., is unknown a priori. To determine $\kappa_k$, Levy et al performed additional experiments and estimated $\kappa_k \approx 3$ (see equations (40, 45) in the Supplementary Information to Ref. [2]). However, $\kappa_k$ is treated as a fitting parameter when BLT data are analyzed, as described below.

Levy et al apply the following procedure to infer the lineage selection coefficients $s$ from the BLT data. At each cycle $k$, they first infer the mean fitness $\bar{s}_k$ using low-frequency lineages, i.e. those with read counts $20 \le r_{k-1} \le 40$. The assumption is that these lineages are so small in size that they have not acquired any adaptive mutations, and so the selection coefficient $s$ for such lineages is zero. For such lineages, equation (S8) simplifies to equation (1) in the main text, which can be used to infer $\mu_k$ and therefore $\bar{s}_k$. However, instead, Levy et al construct the entire empirical distribution for $r_k$ and fit equations (S7), (S8) to it, using $\bar{s}_k$ and $\kappa_k$ as free parameters (see equations (45)–(48) in the Supplementary Information to Ref. [2]).

Having inferred the $\bar{\boldsymbol{s}} = (\bar{s}_1, \bar{s}_2, \dots)$ and $\boldsymbol{\kappa} = (\kappa_1, \kappa_2, \dots)$ from low-frequency lineages, the Levy-Blundell approach takes the entire barcode read-count trajectory $\boldsymbol{r}_i = (r_{i1}, r_{i2}, \dots)$ for each lineage $i$ to infer its selection coefficient $s_i$ and the establishment time $\tau_i$, using a Bayesian approach. Specifically, they define the likelihood function for the read-count trajectory $\boldsymbol{r}_i$ as

$$L_i(s,\tau) = \prod_k p\big(r_{ik}; \mu_k(s, \bar{s}_k, \tau), \kappa_k\big), \tag{S9}$$

where $p$ is given by equation (S7) and an expression for $\mu_k(s_i, \bar{s}_k, \tau_i)$ that is adjusted to account for both the beneficial cells with $s_i > 0$ growing from established time $\tau_i$ and the remaining neutral cells (see equations (58)–(62) in the Supplementary Information to Ref. [2]). To determine if the lineage is adapted or neutral, they construct the ratio of posteriors probabilities that a given lineage $i$ is adapted or neutral as

$$B_i(s,\tau) = \frac{P^{\mathrm{pr}}(s_i,\tau_i) \times L_i(s_i,\tau_i)}{P^{\mathrm{pr}}(\text{no mutation}) \times L_i(0,0)},$$

where $P^{\mathrm{pr}}$ denote prior probability for the each hypothesis (see equations (64)–(67) in the Supplementary Information to Ref. [2]). Finally, they find values $\hat{s}_i$ and $\hat{\tau}_i$ that maximize $B_i$. Lineage $i$ is identified as adapted if $B_i(\hat{s}_i, \hat{\tau}_i) > 1$.

### 4.2 The neutral decline method

Here, we describe what we call the "neutral decline" method, which retains the essential features of the Levy-Blundell method but simplifies it. Most importantly, the neutral decline method does not infer the noise parameter $\kappa$ or the establishment time $\tau$. It proceeds as follows.

- For each consecutive time interval $(t_{k-1}, t_k)$, we choose the neutral reference lineages as those having certain read counts $r_{k-1}$ at the beginning of the interval. As discussed in the main text, we choose the neutral reference lineages as either those with $20 \le r_{k-1} \le 40$ (low-frequency reference) or those with $80 \le r_{k-1} \le 100$ (high-frequency reference).
- For each initial read count $r_{k-1}$ within the specified range, we calculate the mean fitness $\bar{s}_k$ using equation (1) in the main text. To obtain the final estimate of $\bar{s}_k$, we average the individual estimates over all initial read counts.
- We repeat the mean fitness calculation for all sampling intervals and thereby obtain the full mean-fitness trajectory $\bar{\boldsymbol{s}} = (\bar{s}_1, \bar{s}_2, \dots)$.
- To infer the fitness effect $s_i$ of lineage $i$, we maximize the log-likelihood function of the lineage trajectory $\boldsymbol{r}_i = (r_{i1}, r_{i2}, \dots)$,

$$\hat{s}_i = \arg\max \ \ell_i(s),$$

where

$$\ell_i(s) = \sum_k \log p\big(r_{ik}; \mu_k(s, \bar{s}_k), \kappa\big)$$

with $p(r; \mu, \kappa)$ given by equation (S7), $\mu_k(s, \bar{s}_k)$ given by equation (S8) and $\kappa = 3$.

- To determine whether lineage $i$ is adapted, we apply the likelihood ratio test to compare the neutral model ($s_i = 0$, null hypothesis) and the adaptive model ($s_i = \hat{s}_i$, alternative hypothesis). The likelihood-ratio test statistic is given by $R_i = -2\,(\ell_i(0) - \ell_i(\hat{s}_i))$. We call the lineage adapted if $\hat{s}_i > 0$ and $R_i \geq 3.84$, which is the 95th percentile of the $\chi^2$-distribution with 1 degree of freedom.

### 4.3 Key assumptions of the Levy-Blundell method

The Levy-Blundell method—and the neutral decline method as its simplified version—is based on two key assumptions. In this section, we discuss their validity and possible consequences for inference if they are violated.

#### 4.3.1 Assumption 1. Low-frequency lineages are neutral

Low-frequency lineages can be used to infer the mean-fitness trajectory as long as these lineages are neutral, i.e., as long as they have not yet acquired adaptive mutations. If the lineages chosen as reference are in fact not neutral, the inferred mean fitness will be biased, as we demonstrate in the main text. Thus, it is critical for the accuracy of the Levy-Blundell method that this assumption holds.

Of course, as adaptation proceeds, all neutral lineages eventually go extinct and this assumption will eventually be violated. The time scale of persistence of neutral lineages depends on the speed of adaptation, which in turn is determined by the availability of adaptive mutations and their fitness effects, and is therefore a priori unknown.

This assumption can be relaxed if we allow for adapted lineages with known fitness to be used as reference. This can be implemented in practice as follows. By definition, in an initially clonal population, most cells are "wildtype" and any lineage can be used as reference at the initial phase of adaptation. Some lineages will acquire adaptive mutations earlier than others and their fitness can be estimated reliably while neutral lineages are still present in the population. These early adapted lineages can be then used as reference themselves until they acquire secondary adaptive mutations.

#### 4.3.2 Assumption 2. Read frequency dynamics faithfully represent lineage frequency dynamics

According to the population genetics theory, the expected rate of change in the frequency $dx/dt$ of a mutant is determined by its current frequency $x$, its selection coefficient $s$ and population's mean fitness $\bar{s}$,

$$\frac{dx}{dt} = x(s - \bar{s}).$$

Taking the mean fitness as constant $\bar{s}_k$ over a short period of time $\Delta t_k = t_k - t_{k-1}$ (e.g., between two consecutive sampling time points $t_{k-1}$ and $t_k$ in the BLT experiment), we integrate this equation and obtain the expected frequency $x_k$ at time $t_k$,

$$x_k = x_{k-1} e^{(s-\bar{s}_k)\Delta t_k}. \tag{S10}$$

Thus, if the lineage frequencies are known, we can solve equation (S10) for $\bar{s}_k$ (if $s$ is known) or for $s$ (if $\bar{s}_k$ is known).

However, $x_k$ are never known with certainty but are estimated. In particular, in BLT experiments, $x_k$ must be estimated from the corresponding barcode read-count data. The Levy-Blundell method (and the neutral decline method as a consequence) makes an implicit but crucial assumption that the dynamics of measured barcode *read* frequencies are an unbiased (albeit noisy) representation of the dynamics of *cell* frequencies. While this assumption is reasonable, it is in general not true.

We will first show that this assumption is indeed implied by equation (S8). Since $\mu_k$ is the expected number of reads at time $t_k$, equation (S8) can also be re-write as

$$\frac{\mathbb{E}\left(r_k | r_{k-1}\right)}{R_k} = \frac{r_{k-1}}{R_{k-1}} e^{(s-\bar{s}_k)\Delta t_k}, \tag{S11}$$

which implies that the expected read frequency $\mathbb{E}\left(r_k | r_{k-1}\right)/R_k$ depends on the previous read frequency $r_{k-1}/R_{k-1}$ in exactly the same way as the cell frequency $x_k$ in equation (S10).

We will now show that equation (S11) is in fact not generally true. Suppose that the measurement process is described by the probability distribution $P_{\text{meas}}(r|x;R)$, which determines the number of reads $r$ that are obtained from a lineage whose actual population frequency is $x$ if the total read depth is $R$. Let us assume that measurement is unbiased, i.e.,

$$\mathbb{E}\left(r|x\right) = \sum_{r=1}^{R} r\, P_{\text{meas}}(r|x;R) = xR, \tag{S12}$$

which is supported by our data (see Figure 4A in the main text). We rewrite $\mathbb{E}\left(r_k | r_{k-1}\right)$ in terms of the measurement process $P_{\text{meas}}$ and the evolutionary process

$P_{\mathrm{evol}}$.

$$\begin{aligned}\mathbb{E}\left(r_k|r_{k-1}\right) &= \int_0^1 dx_{k-1}\int_0^1 dx_k\, P_{\mathrm{evol}}(x_k|x_{k-1})\, P(x_{k-1}|r_{k-1}) \sum_{r_k} r_k P_{\mathrm{meas}}(r_k|x_k)\\ &= R_k \int_0^1 dx_{k-1}\, P(x_{k-1}|r_{k-1}) \int_0^1 dx_k\, x_k\, P_{\mathrm{evol}}(x_k|x_{k-1})\\ &= R_k\, e^{(s-\bar{s}_k)\Delta t_k}\int_0^1 dx_{k-1}\, x_{k-1}\, P(x_{k-1}|r_{k-1})\\ &= R_k\, e^{(s-\bar{s}_k)\Delta t_k}\, \mathbb{E}\left(x_{k-1}|r_{k-1}\right), \end{aligned} \tag{S13}$$

where to simplify the notations we dropped the parameters from the expressions for various probability distributions. Equation (S13) shows that equation (S11) would be true if the expected frequency of lineages represented by $r$ out of $R$ reads is in fact $r/R$,

$$\mathbb{E}\left(x|r\right) = \frac{r}{R}. \tag{S14}$$

Counterintuitively, equation (S14) is in general not true. To demonstrate this, we first use the Bayes' theorem to re-write it as

$$\mathbb{E}\left(x|r\right) = \frac{P_{\mathrm{meas}}(r|x)\, P_{\mathrm{fr}}(x)}{P(r)}. \tag{S15}$$

Here, $P(r)$ is the normalization constant and $P_{\mathrm{fr}}(x)$ is the (a priori) probability that the frequency of the focal lineage in the population is $x$. Note that in general $P_{\mathrm{fr}}(x)$ is not the distribution of lineage frequencies in the population. Indeed, at later time points in the BLT experiment, $P_{\mathrm{fr}}(x)$ for different lineages are different, because they are informed by previous observations. However, at the beginning of the BLT experiment, all lineages are a priori identical, and $P_{\mathrm{fr}}(x)$ can be approximated by the distribution of lineage frequencies in the population. Previous BLT studies have shown that, despite efforts to introduce all barcodes at the same frequency, the initial distributions of barcode frequencies are often be quite broad and may be close to exponential [5]. We now use four specific examples to show that equation (S14) does not hold.

**Example 1. Poisson measurement noise and a uniform frequency distribution.** First, we consider the simplest case, when the prior frequency distribution is uniform and the measurement noise is Poisson, i.e.,

$$\begin{aligned} P_{\mathrm{fr}}(x)\, dx &= dx,\\ P_{\mathrm{meas}}(r|x;R) &= \frac{(xR)^r\, e^{-xR}}{r!}. \end{aligned} \tag{S16}$$

We show in Appendix B, that the posterior distribution for frequency $x$ is approximately a gamma distribution with shape parameter $r+1$ and scale parameter $1/R$, such that

$$\mathbb{E}\left(x|r\right) \approx \frac{r+1}{R}. \tag{S17}$$

Reassuringly, when $r \gg 1$, expression (S17) converges to $r/R$, suggesting that Assumption 2 is reasonably accurate as long as all lineages are represented by many reads. However, at small numbers of reads, expression (S17) can deviate substantially from (B).

**Example 2. Poisson measurement noise and an exponential frequency distribution.** We now consider an exponential frequency distribution, which may be adequate for initial time points [5],

$$P_{\text{fr}}(x)\,dx = N_L e^{-N_L x}\,dx, \tag{S18}$$

where $N_L$ is the number of barcoded lineages, such that $\mathbb{E}\left(x\right) = 1/N_L$. We show in Appendix B, that the posterior distribution for frequency $x$ is approximately a gamma distribution with shape parameter $r+1$ and scale parameter $1/(R+N_L)$, such that

$$\mathbb{E}\left(x|r\right) \approx \frac{r+1}{R+N_L}. \tag{S19}$$

Since $R/N_L \gtrsim 100$ in typical BLT experiments, expression (S19) is still close to $r/R$ for large $r$, but it again can deviate substantially from (B) when the number of reads is small.

**Example 3. Measurement noise with an increasing variance to mean ratio and a Gamma frequency distribution.** We now consider a more realistic noise model, where the variance to the mean ratio increases with the mean according to equation (2) in the main text. We model the read count as a negative binomial random variable with mean $xR$ and the variance to the mean ratio $1+\epsilon xR$.

$$P_{\text{meas}}(r|x;R,\epsilon) = \frac{\Gamma(r+1/\epsilon)}{\Gamma(r+1)\,\Gamma\left(1/\epsilon\right)}(1-p)^r\,p^{1/\epsilon}. \tag{S20}$$

with $p = (1+\epsilon xR)^{-1}$. We also consider a Gamma frequency distribution with shape parameter $\alpha$ and scale parameter $1/\tilde{N}$,

$$P_{\text{fr}}(x) = \frac{\tilde{N}^\alpha}{\Gamma(\alpha)} x^{\alpha-1} e^{-\tilde{N}x}, \tag{S21}$$

which may be a more accurate description of our prior knowledge at later time points with $\alpha/\tilde{N}$ representing the a priori expected frequency. If $\epsilon \ll 1$ and $r \ll 1/\epsilon$, the posterior distribution is approximately Gamma with shape parameter $r + \alpha$ and scale parameter $(R + \tilde{N})^{-1}$ (see Appendix B), such that

$$\mathbb{E}\,(x|r) = \frac{r + \alpha}{R + \tilde{N}}. \tag{S22}$$

**Example 4. Measurement noise with a constant variance to mean ratio and an exponential frequency distribution.** Finally, we consider a noise model where the variance to the mean ratio is constant and equal to $2\kappa$ as in Ref. [2]. We again model the read count as a negative binomial random variable,

$$P_{\text{meas}}(r|x) = \frac{\Gamma(r + k)}{\Gamma(r + 1)\Gamma(k)}(1 - p)^r p^k.$$

where $p = 1/2\kappa$ and $k = xR/(2\kappa - 1)$. In this case, as we show in Appendix B, we have

$$\mathbb{E}\,(x|r) \approx \frac{2\kappa + r}{R}, \quad r = 0, 1, \ldots, R. \tag{S23}$$

Substituting any of the equations (S17), (S19), (S22) or (S23) into equation (S13), we see that the relationship between the expected number of reads at time $t_k$ and the observed number of reads at $t_{k-1}$ is not the same as the relationship (S10) between the expected lineage frequency at time $t_k$ and the actual frequency at $t_{k-1}$. Specifically, unlike the relationship between $\mathbb{E}\,(x_k|x_{k-1})$ and $x_{k-1}$, the relationship between $\mathbb{E}\,(r_k|r_{k-1})$ and $r_{k-1}$ appears to be generally have a non-zero $y$-intercept, whose value depends on the parameters of the prior distribution and measurement noise. However, these two relationships become identical when $r_{k-1}$ is sufficiently large, although how large it needs to be depends on the noise model.

### 4.4 Empirical test of the neutral decline equation

To test the neutral decline equation (equation (1) in the main text), we use the barcode read count data from two BLT simulations (see Section 3) and three BLT experiments: replicate one of the experiment described in Ref. [2], replicate two from the 1-day transfer cycle experiment described in Ref. [6], and replicate 5 of the experiment with the alga described in Ref. [1]. These data sets are referred to as Levy 2015, Li 2019 and Venkataram 2023 in Figure 2, respectively. For each real or simulated BLT experiment, we selected one "early" and one "late" sampling time interval $(t_{k-1}, t_k)$, as described in the caption to Figure 2. For each of the intervals, we calculated the average read count $\bar{r}_k$ for lineages that had the same read count $r_k \in [0, 100]$. To obtain the rescaled average read count $\tilde{r}_k$ we multiplied $\bar{r}_k$ by $R_k/R_{k-1}$. If the number of lineages with a given $r_{k-1}$ was less than five, we did not estimate the corresponding $\tilde{r}_k$.

To investigate whether $\tilde{r}_k$ is a linear function of $r_{k-1}$ as predicted by theory for neutral lineages, we plot $\tilde{r}_k$ against $r_{k-1}$ (see Figure 2 in the main text). For some $r_k$ in some datasets, the dependence of $\tilde{r}_k$ on $r_{k-1}$ becomes very noisy or clearly deviates from linearity, which could be the case, for example, because the lineages are not truly neutral. Thus, we first visually identify the intervals of $r_{k-1}$ where the data behaves approximately linearly. We choose the interval $5 \leq r_{k-1} \leq 15$ for the Li 2019, Venkataram 2023 and the strong-selection simulated datasets at the late time point; we choose the interval $5 \leq r_{k-1} \leq 40$ for the Venkataram 2023 dataset at the early time point; and we choose $5 \leq r_{k-1} \leq 99$ for all the other datasets. Then, using the data only within these intervals, we fit a linear model $\tilde{r}_k \sim a\, r_{k-1} + b$ either with $b = 0$ (restricted model) or with $b$ being a free parameter (full model) and ask whether the full model provides a statistically better fit to our data using the F-test, as described in Section 2.4.

## 5 BASIL

### 5.1 Model of measurement

As discussed in the main text, we cannot directly observe the size $n$ of a barcoded lineage. Instead, a sample from the population is sequenced, and we observe the number of reads that contain the focal barcode.

We model the measurement process with the negative binomial distribution with mean $\langle r \rangle = nR/N$ and variance $\sigma_r^2 = \langle r \rangle + \epsilon \langle r \rangle^2$ (see Section 2.4), where $N$ is total number of cells in the population during sampling, $R$ is the total coverage, and $r$ is the number of reads that contain the focal barcode. The negative binomial distribution is typically parameterized by the number of "successes" $k$ and the success probability $p$, with mean and variance expressed in terms of these parameters as

$$\langle r \rangle = \frac{k(1-p)}{p}$$
$$\sigma_r^2 = \frac{k(1-p)}{p^2} = \frac{\langle r \rangle}{p},$$

which yield

$$k = \frac{p}{1-p} \langle r \rangle = \frac{1}{\epsilon}, \qquad \text{(S24)}$$
$$p = \frac{1}{1 + \epsilon \langle r \rangle}.$$

Thus, the probability of observing $r$ reads for lineage with $n$ cells is

$$P^{\mathrm{m}}(r|n;\epsilon) = \frac{\Gamma\left(r+1/\epsilon\right)}{\Gamma\left(r+1\right)\Gamma\left(1/\epsilon\right)}\left(\frac{\epsilon\left\langle r\right\rangle}{1+\epsilon\left\langle r\right\rangle}\right)^{r}\left(\frac{1}{1+\epsilon\left\langle r\right\rangle}\right)^{\frac{1}{\epsilon}} \tag{S25}$$

with $\langle r\rangle = nR/N$. The measurement distribution (S25) depends on the noise parameter $\epsilon$, which we fit, as well as on $N$ and $R$ which are assumed to be known. Furthermore, this distribution becomes a point measure at $r=0$ if $n=0$ and it converges to the Poisson distribution with mean $\langle r\rangle = nR/N$ as $\epsilon \to 0$.

### 5.2 The belief distribution for lineage size and fitness

Our main object is the belief probability distribution $P^{\mathrm{b}}(n,s\,|\,\boldsymbol{r})$ that a lineage with the observation vector $\boldsymbol{r} = (r_0,\ldots,r_k)$ has size $n$ and selection coefficient $s$. For a given lineage $i$ whose observation vector up to and including time point $t_k$ is $\boldsymbol{r}_{ik} = (r_{i0},\ldots,r_{ik})$, we will sometimes use a simplified notation $P^{\mathrm{b}}_{ik}(n,s)$ instead of $P^{\mathrm{b}}(n,s\,|\,\boldsymbol{r}_{ik})$. For mathematical tractability, we express the belief distribution as

$$P^{\mathrm{b}}(n,s|\boldsymbol{r}) = P^{\mathrm{b}}(n|s,\boldsymbol{r})\,P^{\mathrm{b}}(s|\boldsymbol{r}) \tag{S26}$$

and assume the following parametric forms

$$P^{\mathrm{b}}(n|s,\boldsymbol{r}) = \gamma\left(n\,;\,\varkappa(s,\boldsymbol{r}),\theta(s,\boldsymbol{r})\right), \tag{S27}$$

$$P^{\mathrm{b}}(s|\boldsymbol{r}) = N\left(s\,;\,\mu(\boldsymbol{r}),\sigma^2(\boldsymbol{r})\right), \tag{S28}$$

where $N(s;\mu,\sigma^2)$ is the normal distribution with mean $\mu$ and variance $\sigma^2$ and $\gamma\left(n;\varkappa,\theta\right)$ is the gamma distribution with shape parameter $\varkappa$ and scale parameter $\theta$.

Thus, $P^{\mathrm{b}}(n,s|\boldsymbol{r})$ belongs to a family of distributions with four parameters $\mu$, $\sigma^2$, $\varkappa$, $\theta$ where the latter two parameters can be functions of $s$. In this section, we describe how we update these parameters as the observation vector is augmented from one time point to the next. We do so in two steps, which we refer to as "projection" and "update". During the projection step, which is described in Section 5.2.2, we project the past belief distribution $P^{\mathrm{b}}_{ik-1}$ to obtain the prior distribution $P^{\mathrm{pr}}_{ik}$ for the time point $t_k$. During the update step, which is described in Section 5.2.3, we use the Bayes' theorem to obtain the new belief distribution $P^{\mathrm{b}}_{ik}$ based on the prior distribution $P^{\mathrm{pr}}_{ik}$ and the read count $r_{ik}$ observed at time $t_k$ (see equation (3) in the main text). Importantly, the parametric distribution given by equation (S27) has no weight at $n=0$. In other words, it is valid only for lineages that are present in the population and have not yet gone extinct. We describe how we treat lineage extinctions in Section 5.2.3.

#### 5.2.1 Initialization

At $t_0$, all lineages are expected to be neutral, and we set $\mu = 0$ and $\sigma = 0.1$ for all lineages. For the belief distribution for lineage $i$ with the initial read count $r_{i0}$, we arbitrarily set $\varkappa = r_{i0}/(1 + 0.01\, r_{i0})$ and $\theta = (1 + 0.01\, r_{i0})N/R_0$, where $R_0$ is the total coverage at the sampling time point $t_0$, so that $\mathbb{E}\,(n_0) = Nr_{i0}/R_0$ and the variance to the mean ratio is $(1 + 0.01\, r_{i0})N/R_0$.

#### 5.2.2 Projecting the belief distribution

To obtain the prior distribution $P^{\mathrm{pr}}(n, s|\boldsymbol{r}')$ for the time point $t_k$, where $\boldsymbol{r}' = (r_0, \ldots, r_{k-1})$ is the observation vector up to and including the previous time point $t_{k-1}$, we assume that the belief distribution $P^{\mathrm{b}}(n, s|\boldsymbol{r}')$ at the previous time point is known and has the parametric form given by equations (S27), (S28) with parameters $\mu$, $\sigma$, $\theta$, $\varkappa$. To simplify notations, in this section, we will omit the explicit dependence of all probabilities on the observation vector $\boldsymbol{r}'$.

The sampling time points $t_0, t_1, \ldots$ in the BLT experiment may be separated by one or multiple growth and dilution cycles. We will first consider one such cycle and obtain the "1-cycle projected distribution" $P_1\,(n, s)$ whose parameters we denote as $\mu_1$, $\sigma_1$, $\varkappa_1$, and $\theta_1$. We then use $P_1\,(n, s)$ to obtain the prior probability for $n$ and $s$ after multiple cycles.

To derive $P_1\,(n, s)$, we consider a barcoded lineage that has fitness $s$ relative to the ancestor and that is represented by $n$ cells immediately prior to dilution. At dilution, a fraction $1/D$ of the population is transferred into fresh medium, and the rest is discarded. Immediately after dilution, the lineage's size becomes $n'$, which we model as a Poisson random variable with mean $n/D$, such that

$$P^{\mathrm{dil}}\,(n') = \frac{\left(\frac{n}{D}\right)^{n'} e^{-n/D}}{\Gamma\,(n'+1)}, \quad n' = 0, 1, \ldots$$

We assume that after dilution, the lineage grows deterministically based on the difference between its fitness $s$ and the population's mean fitness $\bar{s}$, such that by the end of the cycle, its size is $n_1 = An'$, where

$$A = De^{(s-\bar{s})\Delta t_c}$$

and $\Delta t_c$ is the cycle duration in generations. Then, the one-cycle transition probability is

$$P^{\mathrm{c}}\,(n_1\,|\,n; s, \bar{s}) = P^{\mathrm{dil}}\left(\frac{n_1}{A}\right) = \frac{\left(\frac{n}{D}\right)^{n_1/A} e^{-n/D}}{\Gamma\left(\frac{n_1}{A}+1\right)}, \tag{S29}$$

where formally $n_1/A = 0, 1, 2, \ldots$. Then, for the 1-cycle projected distribution we have

$$P_1(n_1, s) = \int_0^\infty P^{\mathrm{c}}\left(n_1 \,|\, n; s, \bar{s}\right)\, P^{\mathrm{b}}\left(n, s\right)\, dn. \tag{S30}$$

Integrating equation (S30) with respect to $n_1$, we find, as expected, that the projection does not change the marginal distribution for $s$,

$$P_1(s) = P^{\mathrm{b}}\left(s\right) = N\left(s\,;\,\mu, \sigma^2\right),$$

which implies that $\mu_1 = \mu$ and $\sigma_1 = \sigma$. Since we can express the joint distribution $P_1\left(n_1, s\right)$ through this marginal distribution as $P_1\left(n_1, s\right) = P_1\left(n_1 \,|\, s\right)\, P_1\left(s\right)$, we now only need to obtain the conditional probability $P_1\left(n_1 \,|\, s\right)$. To do so, we note that

$$\begin{aligned} P_1\left(n_1 \,|\, s\right) &= \frac{P_1(n_1, s)}{P_1(s)} = \frac{\int_0^\infty dn\, P^{\mathrm{c}}\left(n_1 \,|\, n; s, \bar{s}_k\right)\, P^{\mathrm{b}}(n \,|\, s)\, P^{\mathrm{b}}(s)}{P^{\mathrm{b}}\left(s\right)} \\ &= \int_0^\infty P^{\mathrm{c}}\left(n_1 \,|\, n; s, \bar{s}_k\right)\, P^{\mathrm{b}}(n \,|\, s)\, dn. \end{aligned} \tag{S31}$$

Substituting expressions (S29) and (S27) into equation (S31), we obtain

$$\begin{aligned} P_1\left(n_1 \,|\, s\right) &= \frac{1}{D^{\frac{n_1}{A}}\,\Gamma\left(\frac{n_1}{A}+1\right)\,\Gamma(\varkappa)\theta^{\varkappa}} \int_0^\infty n^{\tilde{\varkappa}-1} e^{-\frac{n}{\tilde{\theta}}}\, dn \\ &= \frac{\Gamma\left(\frac{n_1}{A}+\varkappa\right)}{\Gamma\left(\frac{n_1}{A}+1\right)\,\Gamma(\varkappa)} \left(\frac{\theta}{D+\theta}\right)^{\frac{n_1}{A}} \left(\frac{D}{D+\theta}\right)^{\varkappa}, \end{aligned} \tag{S32}$$

with $n_1/A = 0, 1, 2, \ldots,\ \tilde{\varkappa} = \frac{n_1}{A} + \varkappa,\ \tilde{\theta} = \frac{\theta D}{\theta + D}$. Equation (S32) shows that, conditional on $s$, $n_1/A$ is distributed as negative binomial with success probability $D/(D+\theta)$ and number of successes $\varkappa$. Therefore,

$$\begin{aligned} \mathbb{E}\left(n_1 | s\right) &= \varkappa\, \theta\, e^{(s-\bar{s})\Delta t_c}, \\ \mathrm{Var}\left(n_1 | s\right) &= \varkappa\, \theta\, (D+\theta) e^{2(s-\bar{s})\Delta t_c}. \end{aligned}$$

Furthermore, the lineage goes extinct after one growth and dilution cycle with probability

$$p_{\mathrm{ext},1}(\theta, \varkappa) = \left(\frac{D}{D+\theta}\right)^{\varkappa} \tag{S33}$$

and survives with probability

$$p_{\mathrm{surv},1}(\theta, \varkappa) = 1 - \left(\frac{D}{D+\theta}\right)^{\varkappa}. \tag{S34}$$

It is then easy to show that the mean and variance conditional on survival are

$$\mathbb{E}\left(n_1 \,|\, s, \mathrm{surv}\right) = \frac{\varkappa\,\theta}{p_{\mathrm{surv},1}(\theta,\varkappa)}\, e^{(s-\bar{s})\Delta t_c}, \tag{S35}$$

$$\mathrm{Var}\left(n_1 \,|\, s, \mathrm{surv}\right) = \frac{p_{\mathrm{surv},1}(\theta,\varkappa)\,(D+\theta+\varkappa\,\theta)-\varkappa\,\theta}{p^2_{\mathrm{surv},1}(\theta,\varkappa)}\varkappa\,\theta\, e^{2(s-\bar{s})\Delta t_c}. \tag{S36}$$

To facilitate further calculations, we approximate the distribution of $n_1$, conditional on $s$ and lineage survival, by a gamma distribution with the shape parameter $\varkappa_1$ and scale parameter $\theta_1$. To determine $\varkappa_1$ and $\theta_1$, we equate the mean $\varkappa_1\theta_1$ and variance $\varkappa_1\theta_1^2$ of this gamma distribution to the conditional mean and variances given by equations (S35) and (S36) and find $\theta_1 = f_\theta(s,\theta,\varkappa)$ and $\varkappa_1 = f_\varkappa(\theta,\varkappa)$ where

$$f_\theta(s,\theta,\varkappa) = \frac{p_{\mathrm{surv},1}(\theta,\varkappa)\,(\theta+D+\varkappa\theta)-\varkappa\theta}{p_{\mathrm{surv},1}(\theta,\varkappa)}\, e^{(s-\bar{s}_k)\Delta t_c}, \tag{S37}$$

$$f_\varkappa(\theta,\varkappa) = \frac{\varkappa\theta}{p_{\mathrm{surv},1}(\theta,\varkappa)\,(\theta+D+\varkappa\theta)-\varkappa\theta}. \tag{S38}$$

Therefore, the full projected probability for a lineage after one growth and dilution cycle is

$$P_1(n,s) = N\left(s;\mu,\sigma\right) \times \begin{cases} p_{\mathrm{ext},1}(\theta,\varkappa)\,, & \text{if } n=0, \\ p_{\mathrm{surv},1}(\theta,\varkappa)\,\gamma\left(n;\varkappa_1,\theta_1\right), & \text{if } n>0, \end{cases}$$

where $p_{\mathrm{ext},1}$ and $p_{\mathrm{surv},1}$ are given by equations (S33) and (S34) and parameters $\theta_1$ and $\varkappa_1$ are the functions of $\theta$ and $\varkappa$ given by equations (S37) and (S38).

If the next sampling occurs at the end of a single growth and dilution cycle after the previous sampling, then we set $P^{\mathrm{pr}}(n,s) = P_1(n,s)$. If $L>1$ growth and dilution cycles elapse between successive samples, then we set the prior probability $P^{\mathrm{pr}}(n,s)$ for the next sampling time point to be equal to

$$P^{\mathrm{pr}}(n,s) = N\left(s;\mu,\sigma\right) \times \begin{cases} 1-p_{\mathrm{surv},L}\,, & \text{if } n=0, \\ p_{\mathrm{surv},L}\,\gamma\left(n;\varkappa_L,\theta_L\right), & \text{if } n>0, \end{cases} \tag{S39}$$

where the $L$-cycle survival probability is given by

$$p_{\mathrm{surv},L} = \prod_{\ell=1}^{L} p_{\mathrm{surv},1}(\theta_\ell,\varkappa_\ell), \tag{S40}$$

and parameters $\theta_\ell = f_\theta\left(s,\theta_{\ell-1},\varkappa_{\ell-1}\right)$, $\varkappa_\ell = f_\varkappa\left(\theta_{\ell-1},\varkappa_{\ell-1}\right)$ for $\ell = 1,2,\dots L$ are obtained recursively using equations (S37) and (S38) with $\varkappa_0 \equiv \varkappa$, $\theta_0 \equiv \theta$. Note that since all $\varkappa_\ell$ and $\theta_\ell$ depend on the parameters $\varkappa$, $\theta$ of the prior belief distribution $P^{\mathrm{b}}(n \,|\, s, \boldsymbol{r}')$, which themselves are functions of $s$, the survival probability $p_{\mathrm{surv},L}$ is also a function of $s$.

#### 5.2.3 Updating the belief distribution

Next, we apply the Bayes' theorem to obtain the belief distribution $P^{\mathrm{b}}(n, s \,|\, \boldsymbol{r})$ after observing the current read count $r_k$,

$$P^{\mathrm{b}}(n, s \,|\, \boldsymbol{r}) = \frac{P^{\mathrm{pr}}(n, s \,|\, \boldsymbol{r}')\; P^{\mathrm{m}}(r_k \,|\, n)}{Z^S(\boldsymbol{r})}, \tag{S41}$$

where $\boldsymbol{r}' = (r_0, \ldots, r_{k-1})$ and $\boldsymbol{r} = (r_0, \ldots, r_{k-1}, r_k)$ are the previous and current observation vectors, $P^{\mathrm{pr}}(n, s \,|\, \boldsymbol{r}')$ is the prior probability that the lineage has selection coefficient $s$ and size $n$ at the current observation time point before the current measurement is made (see equation (S39)), $P^{\mathrm{m}}(r \,|\, n)$ is the probability of observing $r$ reads for a lineage of size $n$ (see equation (S25)), and the denominator

$$Z^S(\boldsymbol{r}) = \int_{-\infty}^{\infty} ds \int_{0}^{\infty} dn\, P^{\mathrm{pr}}(n, s \,|\, \boldsymbol{r}')\; P^{\mathrm{m}}(r_k \,|\, n)\,. \tag{S42}$$

is the marginal probability of observing $r_k$ reads in our model. The superscript $S$ stands for "selection" and denotes the fact that our model allows the selection coefficient of the lineage to vary freely. We will later consider a neutral null model where the selection coefficient is fixed at zero (see Section 5.3). Note that $P^{\mathrm{pr}}$ (and hence $Z^S$ and $P^{\mathrm{b}}$) depends on $\bar{s}_k$, the mean fitness at the current interval $(t_{k-1}, t_k)$, and $P^{\mathrm{m}}$ (and hence $Z^S$ and $P^{\mathrm{b}}$) depends on the noise parameters $\epsilon_k$ at the current interval $(t_{k-1}, t_k)$. For now, we can ignore these dependencies treating these parameters as fixed and known. However, these dependencies will becomes important in Section 5.3, where we discuss how $\bar{s}_k$ and $\epsilon_k$ are estimated.

As discussed above, we would like to approximate the belief distribution with an analytical parametric form given by equations (S27), (S28). However, since the numerator of equation (S41) is a complex function of both $n$ and $s$, the normalization constant (S42) and the moments of this distribution cannot be expressed analytically. Therefore, to estimate the parameters of the belief distribution, we draw a random sample $(n_j, s_j)$, $j = 1, 2, \ldots, M$ from the belief distribution $P^{\mathrm{b}}(n, s \,|\, \boldsymbol{r})$ using the Markov Chain Monte Carlo (MCMC) approach (see Section 5.5 for the details of the algorithm). We consider two cases, $r_k > 0$ and $r_k = 0$, which differ qualitatively because in the former case, the lineage is guaranteed to have survived until the current sampling time point $t_k$ whereas in the later case it is possible that the lineage has gone extinct.

**Guaranteed lineage survival, $r_k > 0$.** If $r_k > 0$, that is, if the lineage is observed at current time point $t_k$, then $P^{\mathrm{b}}(0, s \,|\, \boldsymbol{r}) = 0$ since $P^{\mathrm{m}}(r_k \,|\, 0) = 0$ whenever $r_k > 0$. In other words, if we observe the lineage, we know with certainty that it has survived up to the current sampling time point. We then estimate the parameters $\mu$, $\sigma$ of the distribution (S28) from this sample as

$$\hat{\mu} = \frac{1}{M}\sum_{j=1}^{M} s_j, \tag{S43}$$

$$\hat{\sigma} = \sqrt{\frac{1}{M-1}\sum_{j=1}^{M}(s_j - \hat{\mu})^2}. \tag{S44}$$

Estimating parameters $\varkappa(s)$ and $\theta(s)$ of the conditional distribution (S27) is more difficult because they can be arbitrary functions of $s$. In principle, one could estimate these functions by independently fitting the shape and scale parameters of the gamma distribution for each $s$-slice of the joint distribution. However, this approach is computationally intensive. To reduce computational burden, we sought to find simple functional forms for the functions $\varkappa(s)$ and $\theta(s)$ and fitting the parameters of these functional forms directly from the joint distribution. To this end, we noticed that $\ln n$ and $s$ are linearly correlated in our MCMC samples (see Figure S3), implying that

$$\mathbb{E}\left(\ln n | s\right) \approx \tilde{a} + \tilde{b}\, s. \tag{S45}$$

Since

$$\mathbb{E}\left(\ln n | s\right) = \psi(\varkappa) + \ln\theta \approx \ln\varkappa + \ln\theta,$$

where $\psi$ is the di-gamma function (see Appendix C) and the approximation $\psi(\varkappa) \approx \ln\varkappa$ holds when $\varkappa \gg 1$, we obtain

$$\theta \approx \frac{e^{\tilde{a}+\tilde{b}s}}{\varkappa},$$

which suggests that an exponential dependence of $\theta$ on $s$ should capture the shape of our belief distribution. To simplify subsequent calculations, we use the ansatz

$$\theta\left(s\right) = \frac{a}{\varkappa}\exp\left[\frac{b\left(s-\mu\right)}{\sigma}\right], \tag{S46}$$

where $\varkappa > 0$, $a$ and $b$ are the new real-valued parameters of distribution (S27).

With this parametrization, we have

$$\mathbb{E}\left(n\right) = a\, e^{\frac{b^2}{2}}, \tag{S47}$$

$$\mathrm{Var}\left(n\right) = \frac{a^2 e^{b^2}}{\varkappa}\left((1+\varkappa)e^{b^2} - \varkappa\right), \tag{S48}$$

$$\mathrm{Cov}\left(n, s\right) = ab\sigma\, e^{\frac{b^2}{2}}. \tag{S49}$$

Equations (S47)–(S49) can be solved to yield estimators of the parameters of the gamma distribution (S27) with the functional form (S46),

$$\hat{b} = \frac{\widehat{\mathrm{Cov}}\,(s, n)}{\langle n \rangle\ \hat{\sigma}} \tag{S50}$$

$$\hat{a} = \langle n \rangle\, e^{-\hat{b}^2/2} \tag{S51}$$

$$\hat{\varkappa} = \frac{\hat{a}^2 e^{2\hat{b}^2}}{\widehat{\mathrm{Var}}(n) - \hat{a}^2 e^{\hat{b}^2}(e^{\hat{b}^2} - 1)}, \tag{S52}$$

where

$$\langle n \rangle = \frac{1}{M} \sum_{j=1}^{M} n_j, \tag{S53}$$

$$\widehat{\mathrm{Var}}(n) = \frac{1}{M-1} \sum_{j=1}^{M} (n_j - \langle n \rangle)^2, \tag{S54}$$

$$\widehat{\mathrm{Cov}}(s, n) = \frac{1}{M-1} \sum_{j=1}^{M} (s_j - \hat{\mu})(n_j - \langle n \rangle). \tag{S55}$$

To test how well our parametric form (S27), (S28), (S46) with the parameters fitted using equations (S50)–(S55) fits the MCMC-sampled posterior distribution, we selected four lineages from simulated and real data and then plotted their MCMC-sampled marginal distributions for $s$ and $n$ as well as their joint distribution (Figure S3). To calculate the marginal distribution for $n$, we generated $M' = 10^5$ random samples of $s_j$, $j = 1, \ldots, M'$ from a normal distribution with mean $\hat{\mu}$ and variance $\hat{\sigma}^2$. We then approximate the marginal distribution for $n$ as

$$P^{\mathrm{b}}(n|\boldsymbol{r}) \approx \frac{1}{M'} \sum_{j=1}^{M'} \gamma\left(n\,;\,\hat{\varkappa}, \hat{\theta}_j\right), \tag{S56}$$

where $\hat{\theta}_j = \hat{a}/\hat{\varkappa}\,\exp\left(\hat{b}\,(s_j - \hat{\mu})/\hat{\sigma}\right)$ with $s$. Figure S3 shows that our parametric form captures the MCMC-sampled distribution reasonably well.

**Possible lineage extinction, $r_k = 0$.** If $r_k = 0$, there are two possibilities. With probability $p_{\mathrm{surv},L}$ given by equation (S40), the lineage has survived up to the current sampling time point $t_k$ but was not detected due to sampling noise; or, with probability $1 - p_{\mathrm{surv},L}$, the lineage has gone extinct between $t_{k-1}$ and $t_k$. Therefore, the belief distribution $P^{\mathrm{b}}\,(n, s|\boldsymbol{r})$ has a non-zero weight at $n = 0$,

$$P^{\mathrm{b}}(n, s) = \frac{N\,(s; \mu, \sigma)}{Z^S} \times \begin{cases} 1 - p_{\mathrm{surv},L}\,, & \text{if } n = 0, \\ p_{\mathrm{surv},L}\,\frac{\gamma(n; \varkappa_L, \theta_L)}{(1+\epsilon n R/N)^{1/\epsilon}}, & \text{if } n > 0, \end{cases} \tag{S57}$$

with

$$Z^S = \int_{-\infty}^{\infty} N(s;\mu,\sigma)\left(1 - p_{\text{surv},L} + p_{\text{surv},L}\int_0^{\infty} \frac{\gamma\left(n;\varkappa_L,\theta_L\right)\,dn}{\left(1+\epsilon nR/N\right)^{1/\epsilon}},\right) ds$$
$$\approx \int_{-\infty}^{\infty} N(s;\mu,\sigma)\left(1 - p_{\text{surv},L} + p_{\text{surv},L}\left(\frac{R}{N\theta_L + R}\right)^{\varkappa_L}\right) ds,$$

where the approximation holds when $\epsilon \ll 1$, and we have omitted the dependencies on the observation vector $\boldsymbol{r}$ and mean fitness $\bar{s}_k$.

Equation (S57) shows that the belief distribution can no longer be adequately captured by the parametric form given by equations (S27), (S28). Instead, we use this parametric form to capture the conditional belief distribution, conditional on the lineage having survived until the current sampling time point $t_k$. We do so using the same approach as above, i.e., we obtain an MCMC sample $(n_j, s_j)$, $j = 1, 2, \ldots, M$ from the distribution (S57) and then use equations (S53)–(S55) to estimate the mean and the variance of the lineage size $n$ and the covariance between lineage size and selection coefficient, except we use only those MCMC samples with $n_j > 0$. We then apply equations (S43), (S44), (S50)–(S52) to estimate the parameters of the conditional belief distribution (S27), (S28). Furthermore, if the lineage was not observed at two consecutive time points, we assume that it has gone extinct and stop tracking it.

#### 5.3 Estimating population's mean fitness and the noise parameter

As mentioned above, our model of evolution and consequently our procedure for updating the belief distribution at the "current" time point $t_k$ depends on the population's mean fitness $\bar{s}_k$ during the interval $(t_{k-1}, t_k)$, $k \geq 1$ as a parameter (see equation (S29)). Similarly, our measurement distribution (equation (S25)) depends on the noise parameter $\epsilon_k$ during the interval $(t_{k-1}, t_k)$. So far, we have assumed that both $\bar{s}_k$ and $\epsilon_k$ are fixed and known. However, in reality the true values of $\bar{s}_k$ and $\epsilon_k$ are of course unknown and must be estimated before we update the lineage belief distributions at $t_k$.

We estimate $\bar{s}_k$ and $\epsilon_k$ using the maximum likelihood approach. To this end, we choose 3000 random lineages for which $r_{ik-1} > 0$ and classify each of them as either putatively neutral or putatively adapted (at the current time interval $(t_{k-1}, t_k)$) as described below. We denote the subsets of currently putatively neutral and putatively adapted lineages as $\mathcal{N}_k$ and $\mathcal{A}_k$, respectively. For each putatively adapted lineage $i \in \mathcal{A}_k$, we can treat the normalization constant $Z^S(\boldsymbol{r}_{ik}; \bar{s}_k, \epsilon_k)$ given by equation (S42) as the likelihood of observing the number of reads $r_{ik}$ for that lineage at time $t_k$ under the model with selection. Similarly, for each putatively neutral lineage $i \in \mathcal{N}_k$, we can treat the normalization constant $Z^N(\boldsymbol{r}_{ik}; \bar{s}_k, \epsilon_k)$ under the neutral model described below (see equation (S61)) as the likelihood of observing the number of reads $r_{ik}$ for that lineage at time $t_k$ under the neutral model. The procedures for estimating $Z^S(\boldsymbol{r}_{ik}; \bar{s}_k, \epsilon_k)$ and $Z^N(\boldsymbol{r}_{ik}; \bar{s}_k, \epsilon_k)$ are described below. Since both $Z^S(\boldsymbol{r}_{ik}; \bar{s}_k, \epsilon_k)$ and $Z^N(\boldsymbol{r}_{ik}; \bar{s}_k, \epsilon_k)$ depend on the unknown parameters $\bar{s}_k$ and $\epsilon_k$, we can write down the log-likelihood function for $\bar{s}_k$ and $\epsilon_k$ as

$$L_k(\bar{s}, \epsilon) = \sum_{i \in \mathcal{A}_k} \log Z^S\left(\boldsymbol{r}_{ik}; \bar{s}, \epsilon\right) + \sum_{i \in \mathcal{N}_k} \log Z^N\left(\boldsymbol{r}_{ik}; \bar{s}, \epsilon\right), \tag{S58}$$

and estimate

$$(\bar{s}_k, \epsilon_k) = \arg\max_{\bar{s}, \epsilon} L_k(\bar{s}, \epsilon)$$

using standard optimization algorithms.

**Classification of lineages for estimating mean fitness.** We classify lineages into putatively neutral and putatively adapted to minimize estimation biases that may arise and be amplified over time due to the following positive feedback loop. Evolutionary dynamics in our model depend only on the difference between the fitness of a lineage and the mean fitness (see equations (S37),(S38)), but not on these two quantities individually. Thus, all selection coefficients and the mean fitness can be identified from data only up to a shared constant. We eliminate this ambiguity by assuming that the expected fitness of all lineages is zero at $t_0$ (see Section 5.2.1). In other words, we estimate selection coefficients with respect to population's initial mean fitness. While this condition should be sufficient to accurately estimate the mean fitness and lineage selection coefficients on average, the large uncertainty in our priors can lead to large uncertainties in the initial estimates of lineage fitness (Figure S4) and mean fitness. These initial deviations can over time be "baked in" into the belief distributions because, as mentioned above, additional data can only correct any inaccuracies in the difference between lineage fitness and mean fitness but not in their individual values.

To make this reasoning more concrete, suppose that we do not classify lineages into putatively neutral and adapted and instead use lineage belief distributions to estimate mean fitness, i.e., use only $Z^S$ terms for all lineages in equation (S58). Imagine that to estimate mean fitness $\bar{s}_1$ at the first time interval $(t_0, t_1)$, by chance we pick 3000 lineages whose frequency declines (slightly) more than expected under neutrality. Then, we will (slightly) over-estimate $\bar{s}_1$. As a result, the projected distributions would under-estimate lineage sizes at $t_1$, thus generating an overly strong surprise once the read counts are observed. This would push us to update the belief distribution towards overly high $s$ for many lineages, which would in turn cause an additional over-estimate of the mean fitness at the next time interval $(t_1, t_2)$, etc.

To dampen this positive feedback loop at the mean-fitness estimation step, we assume that all lineages are neutral by default (for the purposes of mean-fitness estimation) unless we have a high degree of confidence that a given lineage is adapted. Specifically, we classify lineage $i$ as putatively adapted for the purposes of estimating $\bar{s}_k$ if

$$\mu_{ik-1} > 3\,\sigma_{ik-1}, \tag{S59}$$

where

$$\mu_{ik-1} = \int_{-\infty}^{\infty} ds \int_0^{\infty} s\, P^{\mathrm{b}}\left(n, s \,|\, \boldsymbol{r}_{ik-1}\right)\, dn,$$
$$\sigma^2_{ik-1} = \int_{-\infty}^{\infty} ds \int_0^{\infty} \left(s - \mu_{ik-1}\right)^2\, P^{\mathrm{b}}\left(n, s \,|\, \boldsymbol{r}_{ik-1}\right)\, dn$$

are the mean and variance of the marginal belief distribution for $s$ for lineage $i$ at time $t_{k-1}$ estimated using equations (S43), (S44). The factor 3 in equation (S59) was chosen so that our confidence that the lineage is in fact adapted (i.e., has a positive $s$) exceeds 99%.

**Neutral model of lineage evolution.** To compute the marginal likelihood $Z^N(\boldsymbol{r}_{ik}; \bar{s}_k, \epsilon_k)$ that $r_{ik}$ reads are observed for lineage $i$ at time point $t_k$ if the lineage is neutral during the time interval $(t_{k-1}, t_k)$, we employ a neutral model that is analogous to the model with selection described above in Sections 5.2.2 and 5.2.3.

As in the model with selection, we start with the belief distribution $P^{\mathrm{b}}\left(n, s \,|\, \boldsymbol{r}'\right)$, where $\boldsymbol{r}' = (r_0, \ldots, r_{k-1})$ is the observation vector up to and including the previous time point $t_{k-1}$. Since the neutral model is formulated only in terms of the lineage size $n$, we first need to obtain the marginal belief distribution $P^{\mathrm{b}}(n \,|\, \boldsymbol{r}')$. This distribution has no analytical expression. Therefore, to facilitate further calculations, we approximate it with a Gamma distribution with the scale parameter $\tilde{\theta}$ and shape parameter $\tilde{\varkappa}$ chosen to match the marginal mean and variance given by equations (S47), (S48) i.e., we set

$$P^{\mathrm{b}}(n \,|\, \boldsymbol{r}') = \gamma(n; \tilde{\varkappa}, \tilde{\theta}),$$
$$\text{with } \tilde{\varkappa} = \frac{\varkappa}{(1+\varkappa)e^{b^2} - \varkappa},$$
$$\text{and } \tilde{\theta} = \frac{a e^{\frac{b^2}{2}}}{\tilde{\varkappa}}.$$

We then obtain the prior distribution for the lineage size at the current time point $t_k$ analogously to equation (S39),

$$\tilde{P}^{\mathrm{pr}}(n) = \begin{cases} 1 - \tilde{p}_{\mathrm{surv},L}\,, & \text{if } n = 0, \\ \tilde{p}_{\mathrm{surv},L}\, \gamma\left(n; \tilde{\varkappa}_L, \tilde{\theta}_L\right), & \text{if } n > 0, \end{cases} \tag{S60}$$

where the $L$-cycle survival probability $\tilde{p}_{\mathrm{surv},L}$ is given by equation (S40) with parameters $\tilde{\theta}_\ell = f_\theta\left(0, \tilde{\theta}_{\ell-1}, \tilde{\varkappa}_{\ell-1}\right)$, $\tilde{\varkappa}_\ell = f_\varkappa\left(\tilde{\theta}_{\ell-1}, \tilde{\varkappa}_{\ell-1}\right)$ for $\ell = 1, 2, \ldots L$ that are obtained recursively using equations (S37) and (S38) with $\varkappa_0 \equiv \tilde{\varkappa}$, $\theta_0 \equiv \tilde{\theta}$. The marginal likelihood under the neutral model is then given by

$$Z^N\left(\boldsymbol{r}; \bar{s}_k, \epsilon_k\right) = \int_0^\infty \tilde{P}^{\mathrm{pr}}\left(n \,|\, \boldsymbol{r}'\right) P^{\mathrm{m}}(r_k \,|\, n)\, dn. \tag{S61}$$

**Numerical estimation of the marginal likelihoods $Z^S$ and $Z^N$.** For every putatively adapted lineage, we estimate the marginal likelihood $Z^S$ given by equation (S42) by first obtaining a random sample $(s_j, n_j)$, $j = 1, 2, \ldots, M$ with $M = 5000$ from the projected distribution $P^{\mathrm{pr}}(n, s; \bar{s})$ for that lineage, which is given by equation (S39), using a standard random-number generator, keeping only those samples with $n_j > 0$, i.e., conditional on lineage survival. We then calculate

$$\hat{Z}^S(\boldsymbol{r}; \bar{s}, \epsilon) = \frac{1}{M}\sum_{j=1}^{M} P^{\mathrm{m}}(r_k \,|\, n_j; \epsilon). \tag{S62}$$

For every putatively neutral lineage, we estimate the marginal likelihood $Z^N$ given by equation (S61) analogously, by first obtaining a random sample $n_j$, $j = 1, 2, \ldots, M$ from the projected distribution $\tilde{P}^{\mathrm{pr}}(n; \bar{s})$ given by equation (S60), keeping only those samples with $n_j > 0$. We then estimate $\hat{Z}^N$ as per equation (S62).

### 5.4 Identification of adapted lineages and estimation of their selection coefficients

As a result of applying BASIL, we obtain a time-varying belief distribution $P^{\mathrm{b}}_{ik}(n, s) \equiv P^{\mathrm{b}}_{ik}(n, s|\boldsymbol{r}_{ik})$ for each lineage $i$ at each sampling time point $t_k$, $k = 1, 2, \ldots$. Our primary interest is in the marginal belief distribution for the selection coefficient $s$, $P^{\mathrm{b}}_{ik}(s) \equiv P^{\mathrm{b}}(s|\boldsymbol{r}_{ik})$ whose mean and variance are $\mu_{ik}$ and $\sigma^2_{ik}$, estimated using equations (S43), (S44). At early time points, we expect these belief distributions to have high variance because they are insufficiently constrained by the data. As data accumulates, the uncertainty in $s$ should decline, particularly for lineages that actually acquired a single adaptive mutation (Figure S4). However, if we wait long enough, secondary adaptive mutations might appear, which could again increase the uncertainty in our belief of $s$. Thus, for each lineage $i$, we find the time point $t_{k'(i)}$ with minimal variance, $k'(i) = \arg\min_k \hat{\sigma}^2_{ik}$. We call lineage $i$ adapted if $\hat{\mu}_{ik'(i)} > \beta\, \hat{\sigma}_{ik'(i)}$ where $\beta$ is the "confidence factor", a hyper-parameter that controls the precision and recall of our inference. As described in the main text, we empirically determine that the confidence factor $\beta = 3.3$ maximizes the F1-score (the harmonic mean of precision and recall) in our simulated data, and use this value for all our analyses. For each lineage $i$ called as adapted, we estimate its selection coefficient as $\hat{s}_i = \hat{\mu}_{ik'(i)}$, and we can calculate the credible interval for it using the standard deviation $\hat{\sigma}_i = \hat{\sigma}_{ik'(i)}$.

**Algorithm 1** BASIL

**procedure** CONSTRUCT SELECTION MODEL ▷ Sec. 5.2
**procedure** CONSTRUCT NEUTRAL MODEL ▷ Sec. 5.3
**procedure** ESTIMATE BELIEF DISTRIBUTIONS
  **for all** lineage $i$ **do**
    **procedure** INITIALIZE BELIEF DISTRIBUTION ▷ Sec. 5.2.1
      $P^{\mathrm{b}}(n, s \,|\, r_{i0}) = N(s; \mu, \sigma)\, \gamma\,(n; \varkappa(r_{i0}), \theta(r_{i0}))$
      with $\mu = 0$, $\sigma = 0.1$, $\varkappa = r_{i0} + 1$, $\theta = (R_0 + N_L)^{-1}$
  **for** time point $k = 1, 2, \ldots, k_{\text{final}}$ **do**
    **procedure** ESTIMATE MEAN FITNESS FOR $(t_{k-1}, t_k)$ ▷ Sec. 5.3
      $\bar{s}_k = \arg\max_{\bar{s}} L_k(\bar{s})$ ▷ Eq. (S58)
    **for all** lineage $i$ **do**
      **procedure** OBTAIN PRIOR DISTRIBUTION FOR $t_k$ ▷ Sec. 5.2.2
        $P^{\mathrm{b}}(n, s \,|\, \mathbf{r}_{ik-1}) \to P^{\mathrm{pr}}(n, s \,|\, \mathbf{r}_{ik-1})$ ▷ Eq. (S39)
      **procedure** UPDATE BELIEF DISTRIBUTION AT $t_k$ ▷ Sec. 5.2.3
        $P^{\mathrm{pr}}(n, s \,|\, \mathbf{r}_{ik-1}) \to P^{\mathrm{b}}(n, s \,|\, \mathbf{r}_{ik})$ ▷ Eq. (S41)
**procedure** IDENTIFY ADAPTED LINEAGES ▷ Sec. 5.4
**procedure** ESTIMATE SELECTION COEFFICIENTS ▷ Sec. 5.4

### 5.5 Implementation

Figure 3 in the main text & Algorithm 1 show the overall BASIL workflow. We implement BASIL in a Python-based software package. For MCMC sampling, this package uses the C library *stan* and the Python package *pystan 2* as an interface between Python and *stan*. MCMC sampling is implemented using the No-U-Turn Sampler (NUTS) sampler, which efficiently generates proposals based on the posterior distribution [7]. For each belief distribution, we obtain $M = 3500$ samples, with a burn-in of 1,000 steps. For the maximum likelihood estimation of mean fitness, we use the Python package *Noisyopt* for the optimization of noisy functions. Noise arise from the fact that we estimate $\hat{Z}^S$ and $\hat{Z}^N$ for every lineage using a Monte Carlo method as described in Section 5.3. The *Noisyopt* package repeatedly evaluates the stochastic target function, then averages over the stochasticity to ensure convergence.

The BASIL analysis of a single BLT experiment with $5 \times 10^5$ lineages sampled at 10 time points took us about 50 hours to complete on AMD Ryzen 5 7600X 6-Core Processor, and we found that the runtime increases linearly in the number of lineages and sampling time points. However, since MCMC samples for different lineages are independent at each time point, our algorithm is easily parallelizable, and a multiprocessing capability has been implemented in BASIL. The number of processors can be set manually depending on the user's environment. In our analyses, we found it convenient to use 12 to 32 processor cores.

## 6 Application of BASIL to simulated and real BLT data

### 6.1 Belief distribution and lineage identification

We first demonstrate how BASIL works on simulated and real data. Figure S4 shows how the belief distribution $P^b_{ik}(n, s)$ changes over time as information about the lineage frequency is accrued. Next, as discussed in the main text, we used simulated data to determine the optimal value of the confidence factor $\beta$, setting $\beta = 3.3$. The confidence factor has a simple interpretation. We use a linear classifier to classify lineages into adapted and neutral. Specifically, if we plot the inferred selection coefficient of each lineage $\hat{s}_i$ and the estimated standard deviation $\hat{\sigma}_i$ of the credible interval around it, then $1/\beta$ is the slope of the line that separates adapted from non-adapted lineages (see Figures S5). We find that lineages form two large clusters (with some finer clustering structure visible in Figures S5B) that contain overwhelmingly adapted or non-adapted lineages, respectively. The classification line $\hat{s}_i = \beta\, \hat{\sigma}_i$ with $\beta = 3.3$ separates these two clusters very well (see Table 1 in the main text).

### 6.2 Performance on simulated data

To analyze the performance of BASIL on lineage identification, we calculate the following standard statistics:

- The number of true positives (TP), i.e., adapted lineages identified as such;
- The number of false positives (FP), i.e., neutral lineages incorrectly identified as adapted;
- The number of true negatives (TN), i.e., neutral lineages identified as such;
- The number of false negatives (FN), i.e., adapted lineages incorrectly identified as neutral;

- Precision, i.e., the fraction of all positives that are true, TP/(TP+FP);
- Recall, or the true positive rate, i.e., the fraction of all adapted lineages that are identified as such, TP/(TP+FN);
- The F1-score, which is the harmonic mean of precision and recall.

All numbers and statistics are reported in Table 1 in the main text. In particular, in the weak selection region, we obtain 31 false positives (precision 98.8%) and 430 false negatives (recall 85.7%), and in the strong selection region, we obtain 7 false positives (precision 99.7%) and 519 false negatives (recall 82.7%). Figure S5 reveals that all false positives, i.e., neutral lineages that are incorrectly identified as adapted, are very close to the classification line, suggesting that in principle their rate could be further reduced by increasing $\beta$ (albeit at the expense of increasing the rate of false negatives). In contrast, false negatives, i.e., adapted lineages that are incorrectly classified as neutral, are distributed broadly within the non-adapted cluster, indicating that no linear classifier that is based solely on $\hat{s}_i$ and $\hat{\sigma}_i$ can achieve a substantially higher recall. This observation suggests that false negative lineages may be statistically indistinguishable from true neutral ones.

To investigate this conjecture, we first plotted the trajectories of all lineages stratified by their predicted and actual class labels. Figures S6 and S7 confirm that the trajectories of false negative lineages are visually indistinguishable from those of truly neutral lineages. One possible explanation for why some adapted lineages behave is if they are neutral is that these lineages have such small population sizes that their dynamics are governed largely by genetic drift, that is, they fail to "establish" [8]. An adapted lineage establishes approximately when its size $n_i$ exceeds the inverse of its selection coefficient $1/s_i$, or equivalently, $n_i s_i > 1$ [8]. Thus, lineages with smaller selection coefficients have a smaller chance of successfully establishing. Consistent with this prediction, we find that false negative lineages have significantly smaller selection coefficients than true positive lineages, with $P < 0.001$ in both cases (Welch's t-test; see Figures S8). Next, for each lineage $i$, we estimated the maximum size it has achieved during the course of the simulation as $\tilde{n}_i = \max_k r_{ik} N_b / R_k$ and plotted the lineage's true selection coefficient $s_i$ against $\tilde{n}_i$. Figure S8 show that for the majority of false negative lineages, we have $\tilde{n}_i s_i < 1$, whereas for the vast majority of true positive lineages, $\tilde{n}_i s_i > 1$, as expected. These observations support our hypothesis that some adapted lineages fail to establish. As a result, their dynamics are highly stochastic and essentially indistinguishable from neutral.

### 6.3 Performance on experimental data

**Mean fitness.** We applied BASIL to experimental BLT data as described in the main text. Since the true mean fitness in the BLT experiments is unknown, we follow Levy et al [2] and validate our estimates by comparing the inferred mean fitness $\bar{s}_k$ to the mean fitness

$$\bar{s}_k^{\mathrm{ADP}} = \sum_i \hat{s}_i \frac{r_{ik}}{R_k}$$

calculated from adaptive lineages, where $\hat{s}_i$ is the inferred selection coefficient of adapted lineage $i$ (see Section 5.4) and $r_{ik}/R_k$ is its estimated frequency at sampling time $t_k$. We find that the two estimates are largely consistent with each other (Figure S9).

**Comparison of replicate mDFEs.** To construct the measured distribution of fitness effects of new mutations (mDFE), we group lineages based on their inferred selection coefficient $\hat{s}_i$ into 1-percent bins as well as a bin of all lineages with $\hat{s}_i \geq 20\%$. The resulting distributions are shown in Figure 7 in the main text, their statistics are reported in Table 2 in the main text and the full data are available in Supplementary Data S1 (Tabs 2–10). We test the consistency of mDFEs across replicates of the same BLT experiment using quantile-quantile (Q-Q) plots (Figure S10). We find that, by this metric, most mDFEs inferred from replicate experiments are consistent with each other, although experiments HY0-C1d [6], HY2-C2d and HY3-C2d [9] show some discrepancies. In addition, we compare the mean, the median, the interquartile range (IQR), and the interdecile range (IDR) across replicate mDFEs using a permutation test. To this end, we create a list of selection coefficients of all lineages identified as adapted in any of the replicates and then randomly reassign each selection coefficient to each replicate $i$ while preserving the original number of selection coefficients in each replicate. We estimate each mDFE statistic of interest for each permuted replicate. Then, for each pair of replicates it in the permuted data, we calculate the absolute value of the difference between the statistic values; and we average them over all pairs to obtain our test statistic. We repeat this permutation procedure 5,000 times to obtain the null distribution for each mDFE statistic and calculate the empirical $P$-value based on this distribution. The results of this more sensitive test are reported in Table S5. It reveals that the mDFEs inferred from different replicates are statistically distinguishable in most cases.

**Down-sampling.** To test how sequencing coverage and sampling effort affect our mDFE inference, we down-sampled the data from Replicate 1 of the original BLT experiment with strain HY0 in the C-2d environment [2]. The original data has the average coverage of $\sim 160$ per lineage, and sampling occurred at 12 time points, at generations 0, 16, 32, 40, 48, 64, 72, 80, 88, 96, 104 and 112. To reduce coverage, for each barcode whose read count is $r_{ik}$ in the original data, we draw a random number $r'_{ik}$ from a Poisson distribution with mean $r_{ik}/16$, such that the average coverage per lineage is $\sim 10$. To reduce sampling effort, we remove sampling time points at generations 40, 72, 88 and 104, such that the total number of samples is reduced from 12 to 8. We then apply BASIL to these two reduced data sets. The results of this analysis are shown in Table S6 and Figure 8 in the main text, and the full data are provided in Supplementary Data S1 (Tabs 11, 12).

## Appendix A Expectation of the noise variance estimator and bias correction

Since the estimate (S4) we calculate its expectation to determine whether it is biased.

$$\mathbb{E}\left(\hat{\sigma}_{pi}\right) = \frac{1}{|X_p|}\sum_{b\in X_p}\left(\mathbb{E}\left(r_{bi}^2\right) - 2\mathbb{E}\left(r_{bi}^2\,\bar{r}_{bi}^2\right) + \mathbb{E}\left(\bar{r}_{bi}^2\right)\right). \tag{S63}$$

To calculate the expectations in the sum of equation (S63), note that

$$\mathbb{E}\left(r_{ib}\,r_{jb}\right) = p_b^2 R_i R_j + \delta_{ij}\,\sigma_{pi}^2.$$

Now, we have

$$\mathbb{E}\left(r_{bi}^2\right) = \left(p_b R_i\right)^2 + \sigma_{pi}^2, \tag{S64}$$

$$\mathbb{E}\left(r_{bi}^2\,\bar{r}_{bi}^2\right) = \frac{R_i}{n_{\text{rep}}}\sum_{j=1}^{n_{\text{rep}}}\frac{\mathbb{E}\left(r_{bi}\,r_{bj}\right)}{R_j} = \left(p_b R_i\right)^2 + \frac{\sigma_{pi}^2}{n_{\text{rep}}}, \tag{S65}$$

$$\mathbb{E}\left(\bar{r}_{bi}^2\right) = \left(\frac{R_i}{n_{\text{rep}}}\right)^2\sum_{j,k=1}^{n_{\text{rep}}}\frac{\mathbb{E}\left(r_{bj}\,r_{bk}\right)}{R_j R_k} = \left(p_b R_i\right)^2 + \left(\frac{R_i}{n_{\text{rep}}}\right)^2\sum_{j=1}^{n_{\text{rep}}}\frac{\sigma_{pj}^2}{R_j^2}. \tag{S66}$$

Substituting equations (S64)–(S66) into equation (S63), we obtain

$$\mathbb{E}\left(\hat{\sigma}_{pi}\right) = \sigma_{pi}^2\left(1 - \frac{1}{n_{\text{rep}}}\right)^2 + \left(\frac{R_i}{n_{\text{rep}}}\right)^2\sum_{j\neq i}\frac{\sigma_{pj}^2}{R_j^2}. \tag{S67}$$

Thus, the estimate (S4) is a biased estimate of $\sigma_{pi}^2$. Note that if all replicates have the same coverage, $R_i = R$, and $\sigma_{pi}^2 = \sigma_p^2$, equation (S67) simplifies to

$$\mathbb{E}\left(\hat{\sigma}_{pi}\right) = \sigma_p^2\left[\left(1 - \frac{1}{n_{\text{rep}}}\right)^2 + \frac{n_{\text{rep}} - 1}{n_{\text{rep}}^2}\right] = \sigma_p^2\left(1 - \frac{1}{n_{\text{rep}}}\right),$$

and correcting the bias is easy. To correct the bias in the general case, we rewrite equation (S67) in the matrix form as

$$\mathbb{E}\left(\boldsymbol{V}_p\right) = A\,\boldsymbol{\sigma}_p^2,$$

where $\boldsymbol{V}_p = \left(\hat{\sigma}_{p1}, \hat{\sigma}_{p2}, \ldots, \hat{\sigma}_{pn_{\text{rep}}}\right)^T$ and $\boldsymbol{\sigma}_p^2 = \left(\sigma_{p1}^2, \sigma_{p2}^2, \ldots, \sigma_{pn_{\text{rep}}}^2\right)^T$ and

$$A = \begin{pmatrix} \left(1 - \frac{1}{n_{\text{rep}}}\right)^2 & \frac{1}{n_{\text{rep}}^2}\frac{R_1^2}{R_2^2} & \cdots & \frac{1}{n_{\text{rep}}^2}\frac{R_1^2}{R_{n_{\text{rep}}}^2} \\ \frac{1}{n_{\text{rep}}^2}\frac{R_2^2}{R_1^2} & \left(1 - \frac{1}{n_{\text{rep}}}\right)^2 & \cdots & \frac{1}{n_{\text{rep}}^2}\frac{R_2^2}{R_{n_{\text{rep}}}^2} \\ \vdots & \vdots & \ddots & \vdots \\ \frac{1}{n_{\text{rep}}^2}\frac{R_{n_{\text{rep}}}^2}{R_1^2} & \frac{1}{n_{\text{rep}}^2}\frac{R_{n_{\text{rep}}}^2}{R_2^2} & \cdots & \left(1 - \frac{1}{n_{\text{rep}}}\right)^2 \end{pmatrix},$$

and obtain the bias-corrected estimate

$$\boldsymbol{V}'_p = A^{-1}\boldsymbol{V}_p. \tag{S68}$$

## Appendix B Expected frequency given the number of reads

Here, we derive the expressions (S17)–(S23) for the expected frequency of a lineage $\mathbb{E}\,(x|r)$, given the number of reads $r$.

**Example 1. Poisson measurement noise and a uniform frequency distribution.** We first consider the case when the measurement process is modeled by the Poisson distribution (S16) and the frequency distribution is uniform. Then, the posterior distribution for the frequency $x$ is given by

$$P_{\text{post}}(x|r)\,dx = \frac{P_{\text{meas}}(r|x)\,P_{\text{fr}}(x)}{P(r)}\,dx = C\,x^r\,e^{-xR}\,dx, \quad \text{with } x \in [0,1], \tag{S69}$$

where $C$ is the normalization constant. As long as $r/R \ll 1$, this distribution is well approximated by the gamma distribution with the shape parameter $k = r+1$ and scale parameter $\theta = 1/R$. Thus, $C = R^{r+1}/r!$ whose mean is $k\theta = (r+1)/R$ confirming (S17).

**Example 2. Poisson measurement noise and an exponential frequency distribution.** If the frequency distribution is exponential with mean $1/N_L$ (see equation (S18)), then we have

$$P_{\text{post}}(x|r)\,dx = C\,x^r\,e^{-x(R+N_L)}\,dx, \tag{S70}$$

which is again is very well approximated by the gamma distribution with the shape parameter $k = r+1$ and scale parameter $\theta = 1/(R+N_L)$, as long as $r \ll R$. Expression (S19) follows immediately.

**Example 3. Measurement noise with an increasing variance to mean ratio and a Gamma frequency distribution.** We next consider the case when the frequency distribution is Gamma with shape parameter $\alpha$ and scale parameter $1/\tilde{N}$ (see equation (S21)) and the read count is a negative binomial random variable with mean $xR$ and the variance to the mean ratio $1+\epsilon xR$ (see (S20)). When $xR \ll 1/\epsilon$, the negative binomial distribution (S20) converges to the Poisson distribution with mean $xR$, which implies that the posterior distribution for $x$ is a Gamma distribution with shape parameter $r+\alpha$ and scale parameter $(R+\tilde{N})^{-1}$. More generally, the posterior distribution for $x$ given $r$ is given by

$$P_{\text{post}}(x|r)dx = \frac{P_{\text{meas}}(r|x)\,P_{\text{fr}}(x)}{P(r)}dx = C\,x^{r+\alpha-1}(1+\epsilon Rx)^{-(1/\epsilon+r)}\,e^{-\tilde{N}x}\,dx. \tag{S71}$$

It is possible to use Laplace's method to obtain approximate expressions for the normalization constant $C$ and the moments of this distribution. Specifically, whenever $\epsilon \ll 1$, we obtain

$$C = \frac{\tilde{N}+R}{\sqrt{8\pi(r+\alpha-1)}}\,\frac{(1+\epsilon R\bar{x}_0)^{\frac{1}{\epsilon}+r}}{\bar{x}_0^{\alpha+r+1}}\,e^{\tilde{N}\bar{x}_0}$$
$$\mathbb{E}\left(x^i|r\right) = (\bar{x}_i)^i\left(\frac{r+\alpha+i-1}{r+\alpha-1}\right)^{r+\alpha-\frac{1}{2}}\left(\frac{1+\epsilon R\bar{x}_0}{1+\epsilon R\bar{x}_i}\right)^{\frac{1}{\epsilon}+r} e^{-\frac{\tilde{N}i}{\tilde{N}+R}},$$

for $i, r = 1, 2, \ldots$, where $\bar{x}_i = \frac{r+\alpha+i-1}{\tilde{N}+R}$.

**Example 4. Measurement noise with a constant variance to mean ratio and an exponential frequency distribution.** Finally, we consider the case when the measurement process is over-dispersed but with a constant variance to the mean ratio $2\kappa$ (see equation (S20)) and the frequency distribution is exponential (see equation (S18)). To facilitate analytical tractability, instead of tracking frequency $x$ of the lineage in the population, we will track its lineage size $n$. We assume that $n$ take values $1, 2, \ldots$ and that its prior distribution is geometric with parameter $q$. The expected lineage size is $N/N_L$, which implies that $q = N_L/N$. Thus, for the posterior distribution for $n$, given $r$ is

$$P_{\text{post}}(n|r) = \frac{P_{\text{meas}}(r|n)\,P_{\text{fr}}(n)}{P(r)} = \frac{1}{P(r)}\,\frac{\Gamma(r+k)}{\Gamma(r+1)\Gamma(k)}(1-p)^r p^k\,(1-q)^{n-1}q, \tag{S72}$$

where $p = 1/2\kappa$, $k = \alpha n$, $q = N_L/N$ and $\alpha = R/N(2\kappa-1)^{-1}$. Expression (S72) suggests that the quantity $\tilde{k} = \alpha n - 1$ is a negative binomial random variable with parameters

$$\tilde{r} = r+1, \tag{S73}$$
$$\tilde{p} = 1 - p\,(1-q)^{1/\alpha},$$

that is,

$$P_{\text{post}}(\tilde{k}|r) = C\frac{\Gamma\left(\tilde{k}+\tilde{r}\right)}{\Gamma\left(\tilde{k}+1\right)}\,(1-\tilde{p})^{\tilde{k}}\,, \tag{S74}$$

where $C = \tilde{p}^{\tilde{r}}/\Gamma(\tilde{r})$ is the normalization constant. Thus, we have

$$\mathbb{E}\left(\tilde{k}\middle|r\right) = \frac{\tilde{r}(1-\tilde{p})}{\tilde{p}} = \frac{p\,(r+1)\,(1-q)^{1/\alpha}}{1-p\,(1-q)^{1/\alpha}}.$$

Since $\tilde{k} = x\frac{R}{2\kappa-1} - 1$, we have

$$\mathbb{E}\,(x|r) = \frac{2\kappa-1}{R}\frac{2\kappa + r\,(1-q)^{1/\alpha}}{2\kappa-(1-q)^{1/\alpha}} \approx \frac{2\kappa+r}{R},$$

where the last approximation holds because $q = N_L/N \sim 0.01 \ll 1$.

It will also be useful to calculate the expected number of cells in a lineage whose measured number of reads is $r$,

$$\mathbb{E}\,(n|r) = \frac{\mathbb{E}\left(\tilde{k}|r\right)+1}{\alpha} \approx \frac{N}{R}\,(2\kappa+r)\,, \tag{S75}$$

and its variance

$$\begin{aligned}\mathrm{Var}\,(n|r) &= \frac{1}{\alpha^2}\,\mathrm{Var}\left(\tilde{k}|r\right) = \frac{1}{\alpha^2}\,\frac{\tilde{r}(1-\tilde{p})}{\tilde{p}^2}\\ &= \frac{1}{\alpha^2}\,\frac{(r+1)p\,(1-q)^{1/\alpha}}{(1-p\,(1-q)^{1/\alpha})^2} \approx \frac{N^2}{R^2}\,2\kappa\,(r+1)\,.\end{aligned} \tag{S76}$$

## Appendix C Expectation of the logarithm of a gamma-distributed random variable

Consider a random variable $X$ that is Gamma-distributed with scale parameter $\theta$ and shape parameter $\varkappa$, such that its probability density is

$$p(x) = \frac{1}{\Gamma(\varkappa)\,\theta^{\varkappa}}\,x^{\varkappa-1}\,e^{-\frac{x}{\theta}}.$$

Therefore, we have

$$\int_0^\infty x^{\varkappa-1}\,e^{-\frac{x}{\theta}}\,dx = \Gamma(\varkappa)\,\theta^{\varkappa}. \tag{S77}$$

Differentiating both sides of equation (S77) with respect to $\varkappa$, we obtain

$$\int_0^\infty \ln x\,x^{\varkappa-1}\,e^{-\frac{x}{\theta}}\,dx = \Gamma(\varkappa)\,\theta^{\varkappa}\left(\frac{\Gamma'(\varkappa)}{\Gamma(\varkappa)}+\ln\theta\right),$$

which can be rewritten as

$$\mathbb{E}\,(\ln X) = \psi(\varkappa) + \ln\theta,$$

where $\psi(\varkappa) = \frac{\Gamma'(\varkappa)}{\Gamma(\varkappa)}$ is the digamma function.

## 7 Supplementary Figures

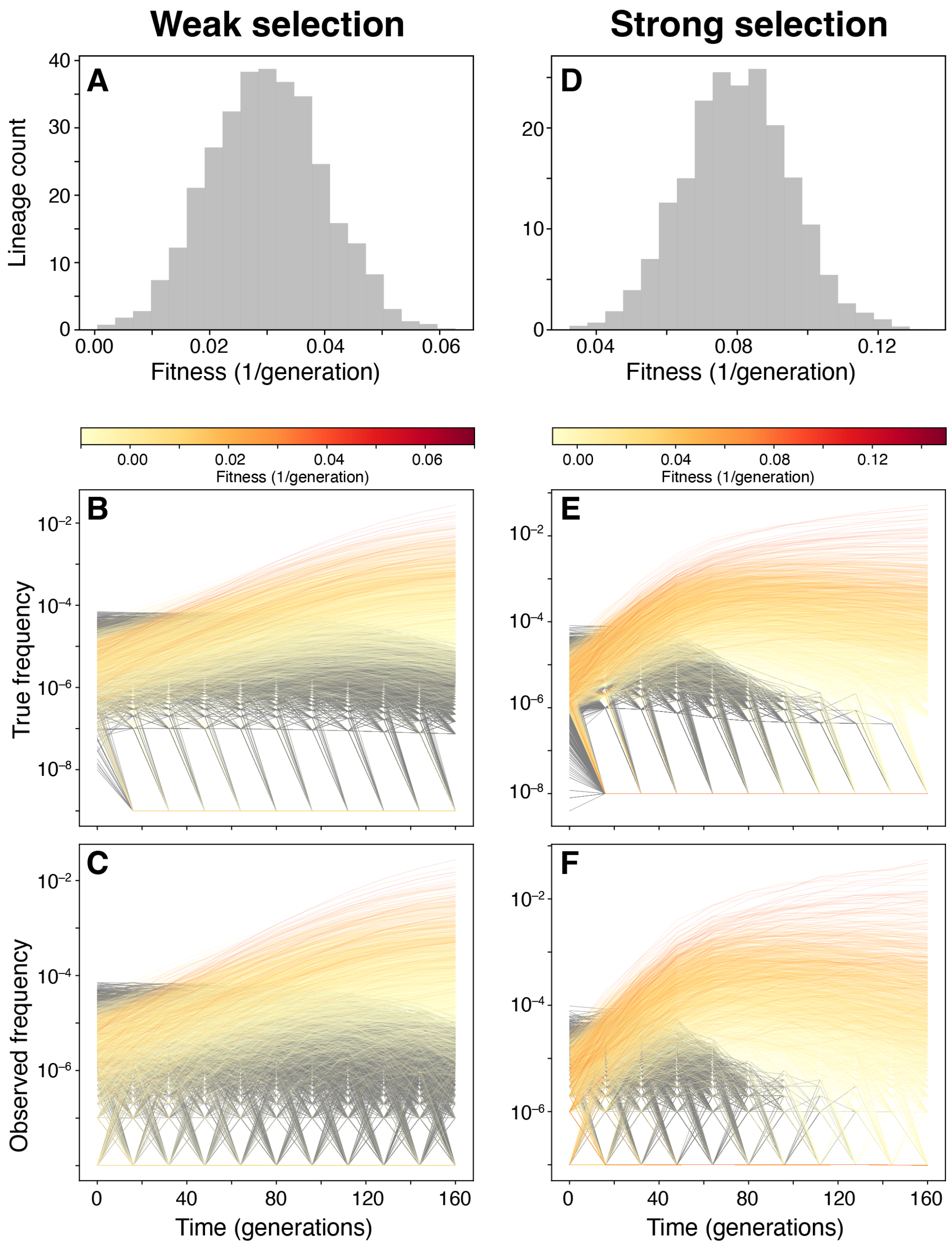

**Figure S1. Simulated data. A–C.** Weak selection regime. **D–F.** Strong selection regime. Distributions of lineage fitness (A,D). True lineage frequency trajectories (B,E). Observed barcode frequency trajectories (C,F). Adapted lineages are colored by their fitness, neutral lineages are gray.

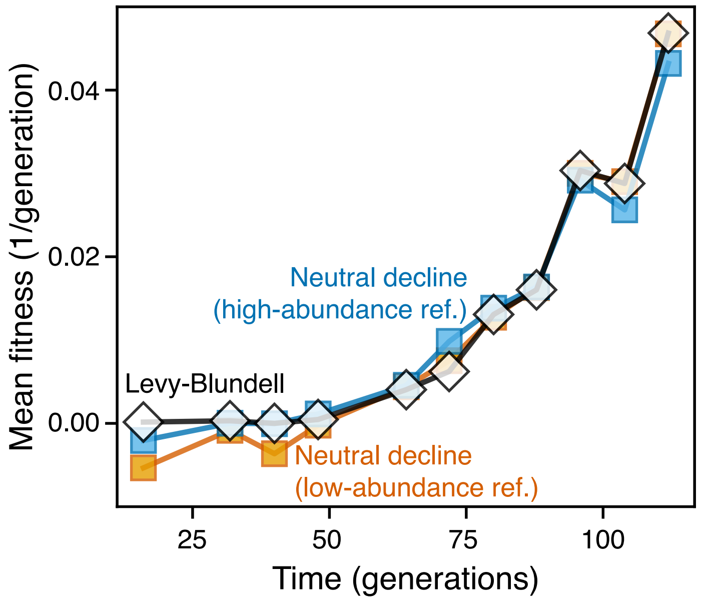

**Figure S2. Correspondence between the full Levy-Blundell inference method and the simplified neutral decline method.** Mean-fitness trajectories in Replicate 1 in the Levy 2015 dataset as reported in Ref. [2] (black line and white diamonds), and inferred by our the neutral decline method using either high-abundance lineages (blue line and squares) or low-abundance lineages (orange line and squares) as reference.

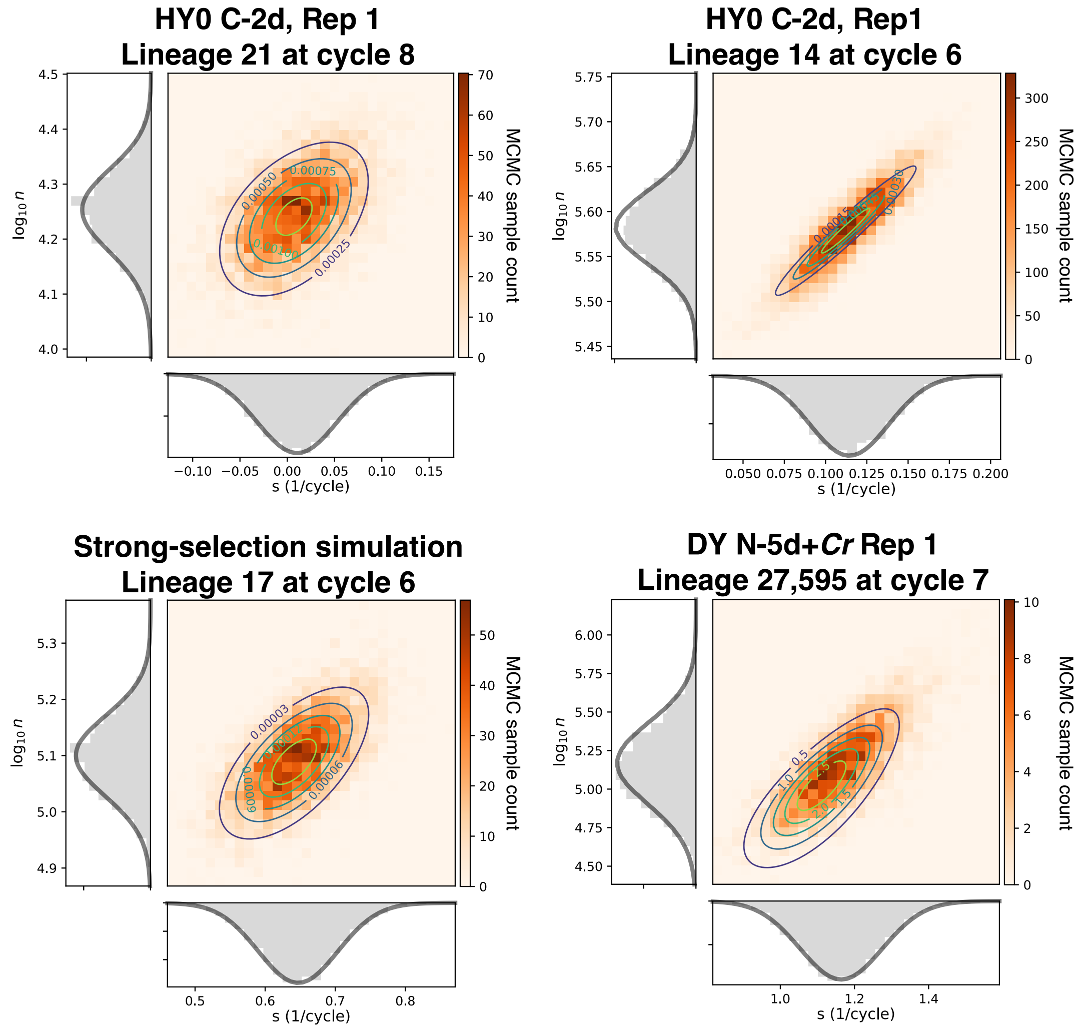

**Figure S3. Estimation of the belief distribution $P^{\mathrm{b}}(n, s)$.** Each panel corresponds to a single lineage in a particular dataset at a certain time point, as indicated in the panel title. The heatmap in each panel shows the number of MCMC samples in each $(s, \log_{10} n)$ bin. The contour lines correspond to the analytical function $P^{\mathrm{b}}(n, s)$ given by equations (S26)–(S28) with parameters fitted to the MCMC sample. Histograms show the marginal distributions for $s$ (bottom) and $\log_{10} n$ (left) and the corresponding parametric curves. See Section 5.2.3 for details.

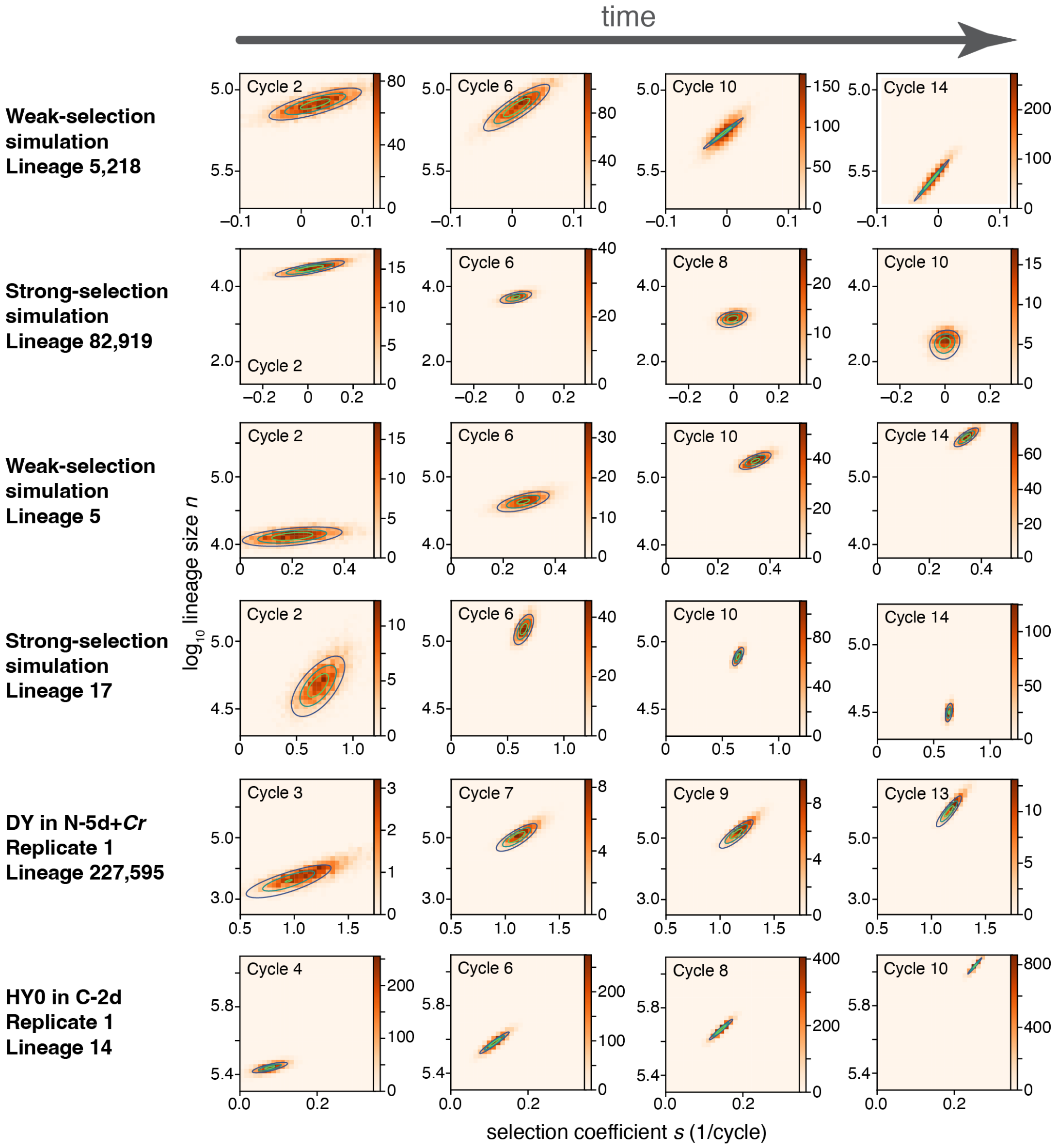

**Figure S4. Changes in the belief distribution as data is accumulated.** Each row corresponds to a single lineage in a particular dataset, as indicated on the left. Notations are as in Figure S3.

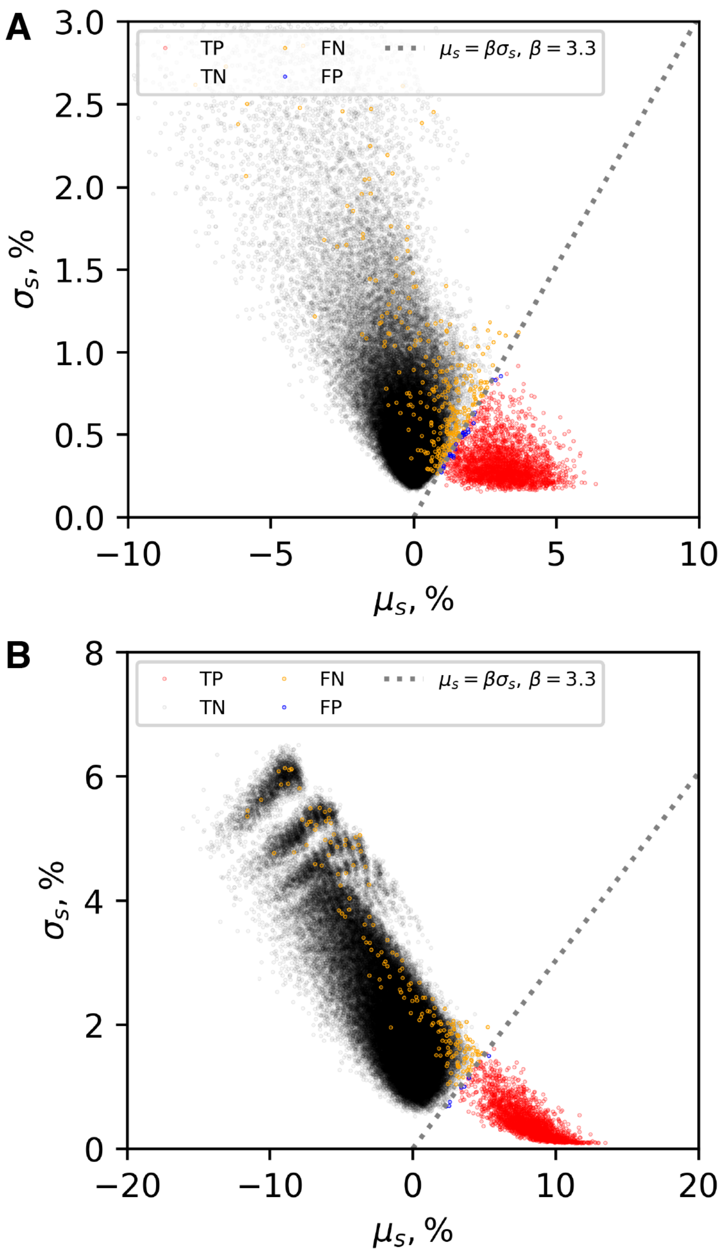

**Figure S5. Classification of lineages in simulated data. A.** Mean ($x$ axis) versus the standard deviation ($y$ axis) of the estimated marginal belief distribution for the lineage selection coefficient for simulated data in the weak selection regime. **B.** Same for the strong-selection regime. Each point represents a lineage. Colors represent different lineage classes: red = true positives, black = true negatives, orange = false negatives, blue = false positives. Dashed line is the classification line with $\beta = 3.3$.

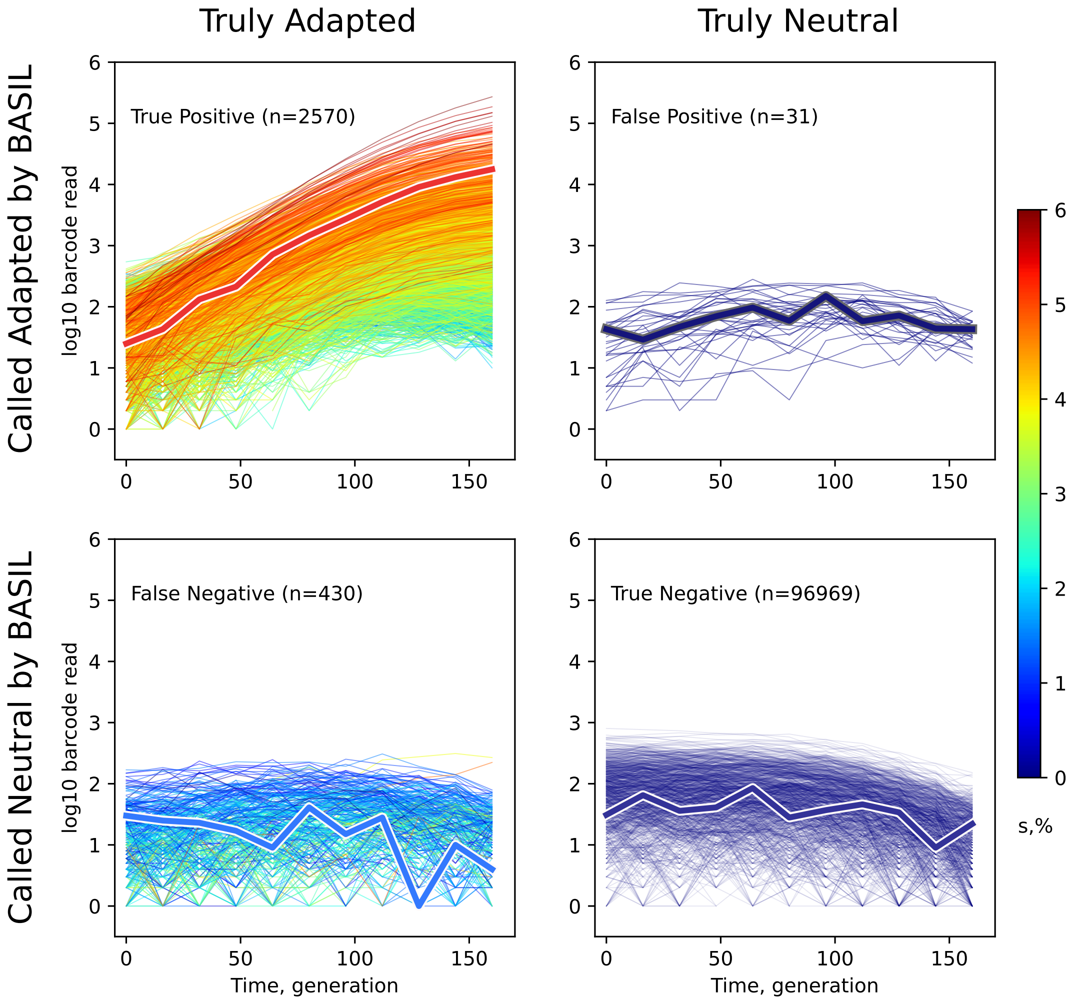

**Figure S6. Lineage trajectories in the weak-selection simulation stratified by class.** Data is the same as in Figure S1B. Only 970 random true negative lineages are shown to improve visualization. Trajectories are colored by lineage fitness.

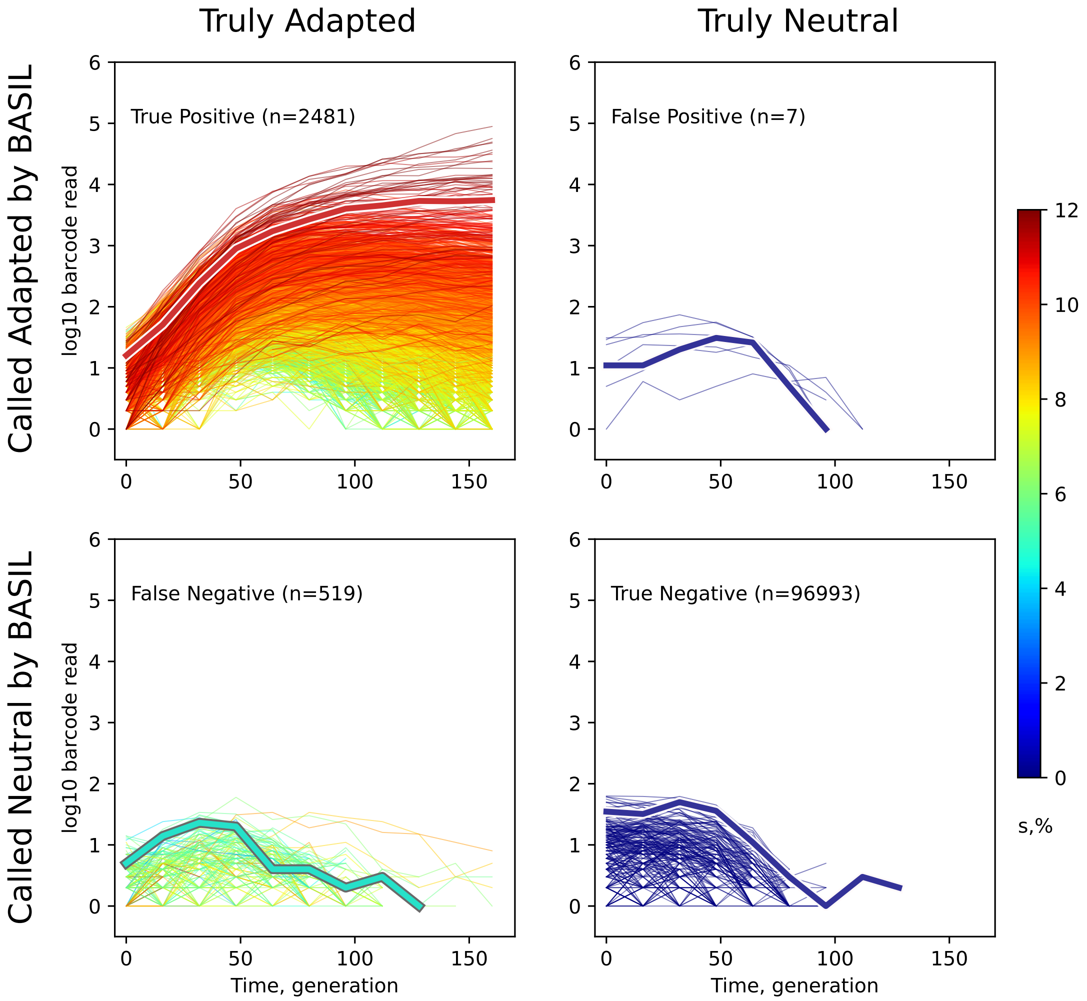

**Figure S7. Lineage trajectories in the strong-selection simulation stratified by class.** Data is the same as in Figure S1E. Only 243 random true negative lineages are shown to improve visualization. Trajectories are colored by lineage fitness.

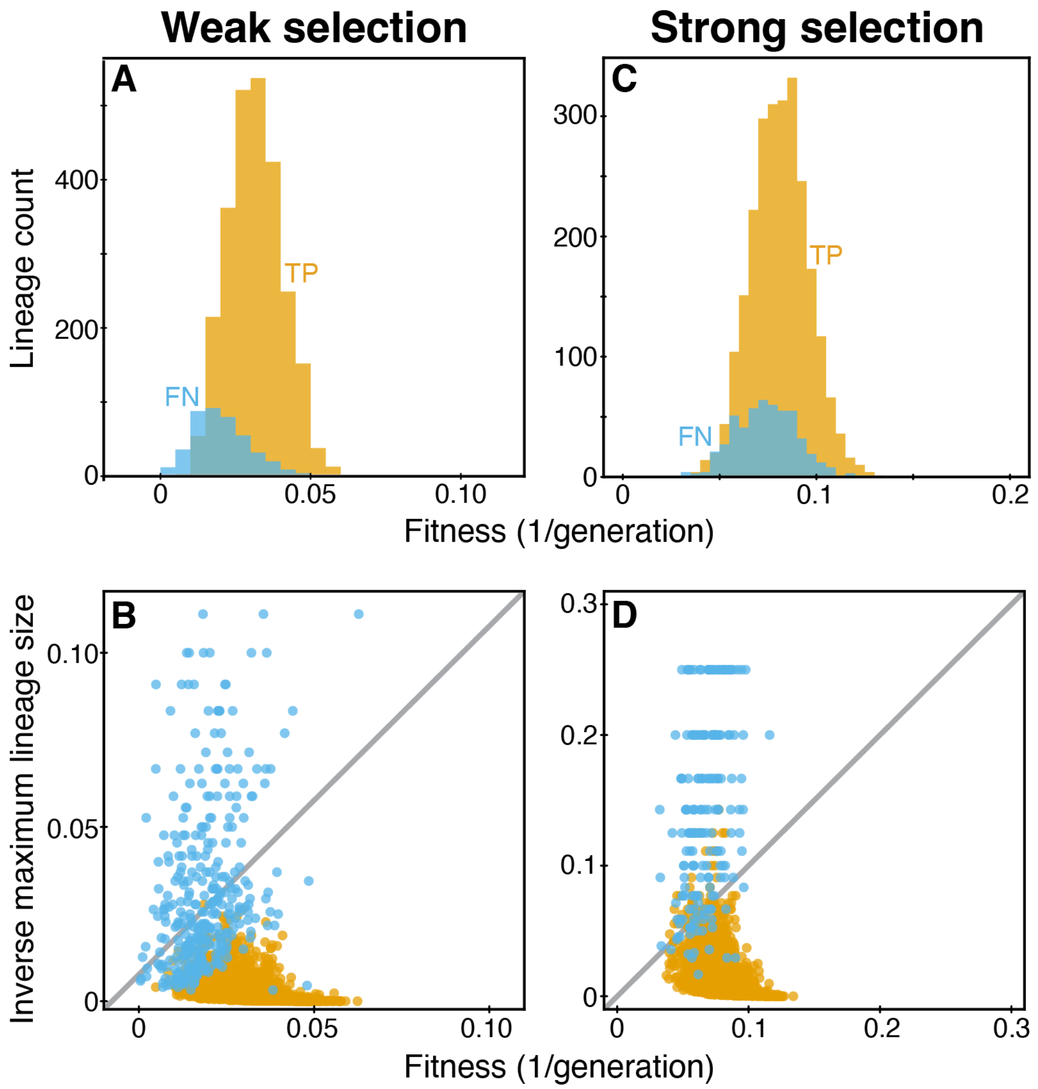

**Figure S8. True positive and false negative lineages have distinct features in simulated data. A.** Distribution of selection coefficients of true positives (yellow) and false negative (blue) lineages in the weak-selection simulation. **B.** The relationship between lineage fitness $s_i$ and its maximum size $\tilde{n}_i$ in the simulation (see Section 6.2 for details). Lineages above the diagonal (gray line) are those with $\tilde{n}_i s_i < 1$, and lineages below the diagonal are those with $\tilde{n}_i s_i > 1$. **C.** Same as panel A but for the strong-selection simulation. **D.** Same as panel B but for the strong-selection simulation.

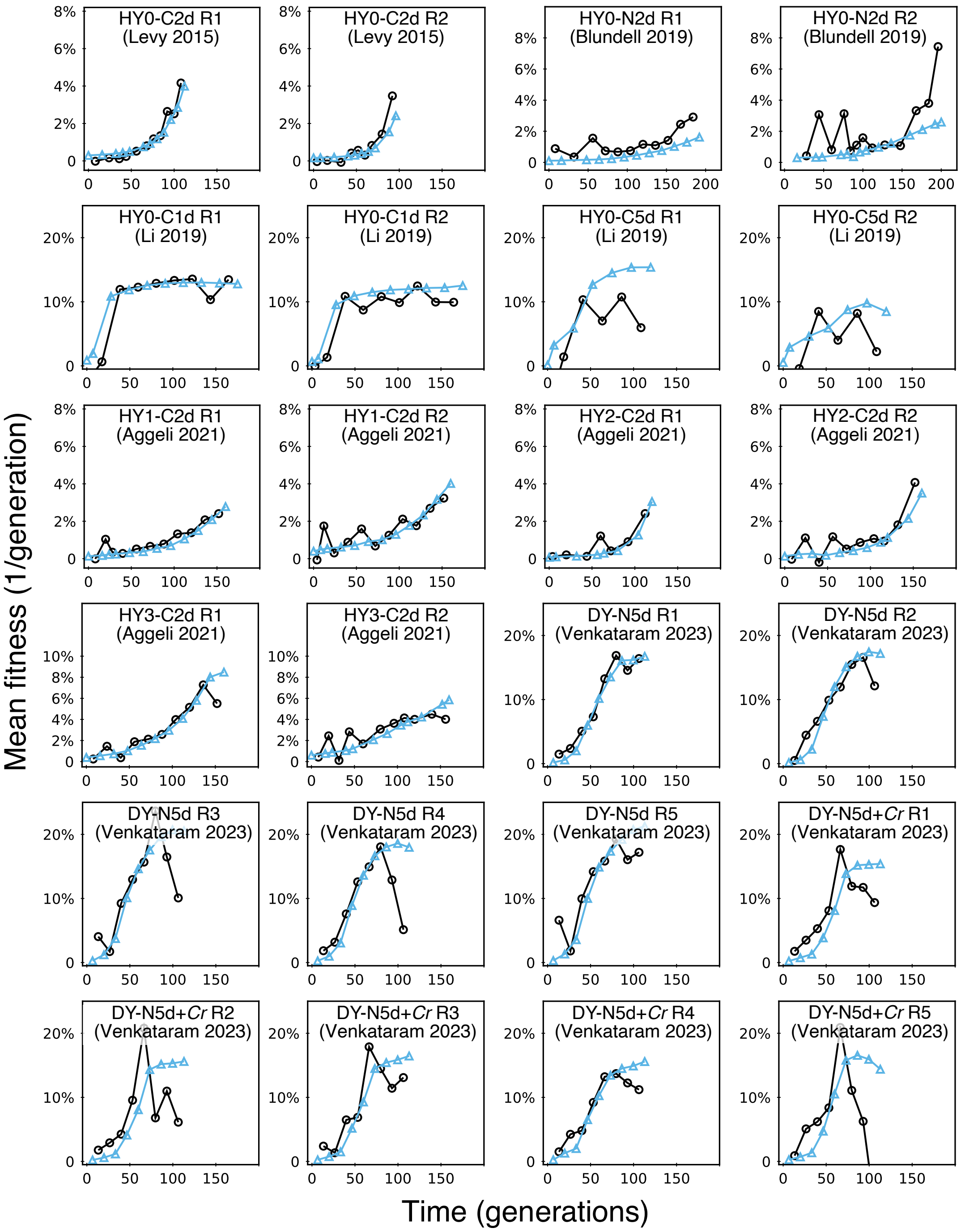

**Figure S9. Mean fitness trajectories in published BLT datasets.** Each panel represents a replicate of a BLT experiment, as indicated. Black lines show trajectories inferred by BASIL; blue lines show trajectories calculated from lineages identified as adapted.

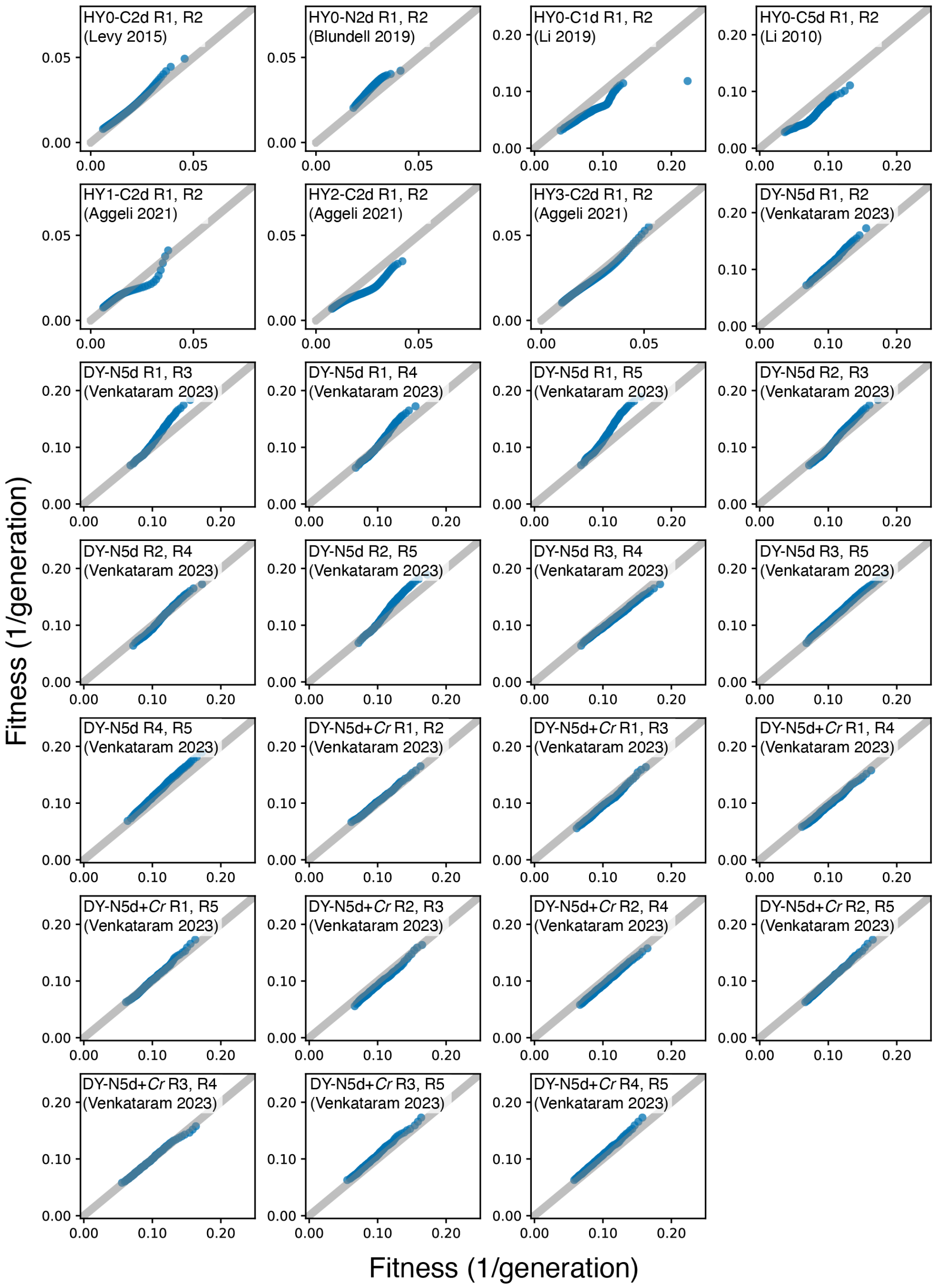

**Figure S10. Quantile-quantile plots for the measured distributions of fitness effects (mDFEs) inferred across replicates.** Each panel represents a pair of replicates from the same BLT study, as indicated. 100 quantiles are shown in each panel.

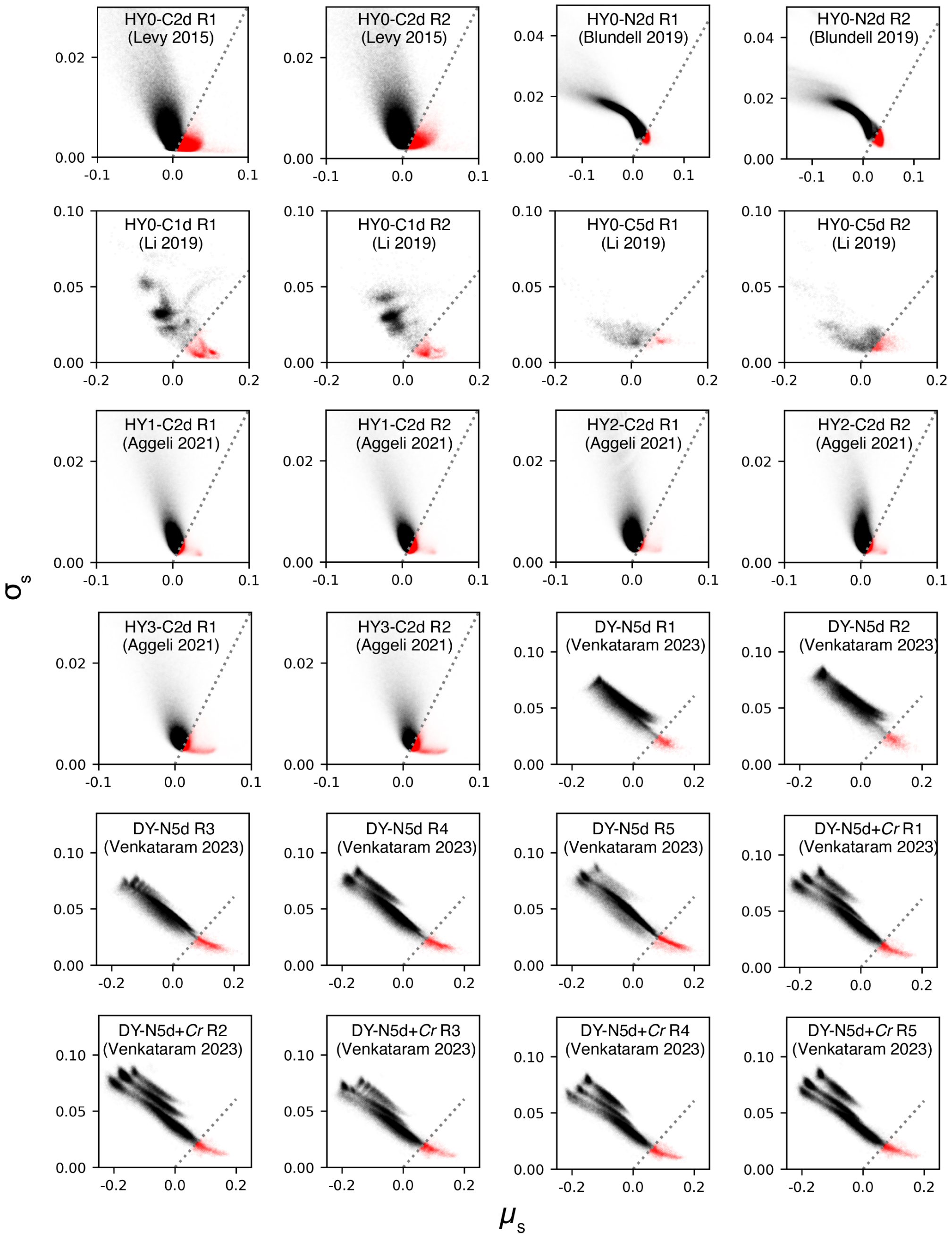

**Figure S11. Classification of lineages in real BLT data.** Each panel represents a replicate of a BLT experiment, as indicated. Notations are as in Figure S5.

## 8 Supplementary Tables

| | Linear ($\epsilon = 0$) | | Quadratic ($\epsilon \neq 0$) | |
|---|---|---|---|---|
| | Poisson ($a = 1$) | General – | 1-param ($a = 1$) | General – |
| Fitted $\hat{a}$ | – | 4.34 | – | 1.97 |
| Fitted $\hat{\epsilon}$ | – | – | 0.0024 | 0.0016 |
| RSS[1] | 173.6 | 85.2 | 55.1 | 48.3 |
| $R^2$ | 0.77 | 0.89 | 0.93 | 0.94 |

**Table S1. Model fitting for the relationship between read-count mean and variance.** [1]Residual sum of squares.

| Null model | | | Alternative model | | | | |
|---|---|---|---|---|---|---|---|
| Name | $p_{\text{null}}$ | $\text{RSS}_{\text{null}}$ | Name | $p_{\text{alt}}$ | $\text{RSS}_{\text{alt}}$ | $F$ | $P$-value |
| Poisson | 0 | 173.6 | Lin. Gen. | 1 | 85.2 | 41.5 | $1.13 \times 10^{-7}$ |
| Poisson | 0 | 173.6 | Quad. 1-param. | 1 | 55.1 | 50.7 | $1.63 \times 10^{-11}$ |
| Poisson | 0 | 173.6 | Quad. Gen. | 2 | 48.3 | 86.0 | $1.44 \times 10^{-11}$ |
| Lin. Gen. | 1 | 85.2 | Quad. Gen. | 2 | 48.3 | 29.9 | $2.87 \times 10^{-6}$ |
| Quad. 1-param. | 1 | 55.1 | Quad. Gen. | 2 | 48.3 | 5.56 | 0.0235 |

**Table S2. Goodness of fit comparison between models fitting the relationship between read-count mean and variance.** In all cases $n = 41$.

| Parameter | Selection | |
|---|---|---|
| | Weak | Strong |
| $\langle s \rangle$ | 0.03 | 0.08 |
| $\sigma_s$ | 0.01 | 0.015 |
| $\langle N \rangle$ | $2.56 \times 10^9$ | $2.56 \times 10^8$ |
| $\langle N_b \rangle$ | $10^7$ | $10^6$ |
| $R$ | $10^7$ | $10^6$ |
| $\sigma_r^2$ | $6 \langle r_i \rangle$ | $\langle r_i \rangle + 0.01 \langle r_i \rangle^2$ |

**Table S3.** Simulation parameters. For the weak selection regime, we chose the relationship between mean read count $\langle r_i \rangle$ and variance in read count $\sigma_r^2$ to be linear in order to make our simulations as similar to the work by Levy et al [2] as possible. For the strong selection regime, we chose the more realistic quadratic relationship, based on our results (see Section 2).

| Notation | Description | Eqns |
|---|---|---|
| $k$ | sampling time index, $k \in [0, k_\mathrm{f}]$ | |
| $t_k$ | time (in generation) of the $k$th sample | |
| $R_k$ | total read depth at $t_k$ | |
| $D$ | dilution ratio, $D > 1$ | |
| $N$ | population size before dilution | |
| $N_b$ | population size after dilution, $N_b = N/D$ | |
| $\epsilon$ | measurement error parameter | (2), (S5) |
| $s_i$ | selection coefficient (fitness) of the $i$th lineage | |
| $\bar{s}_k$ | mean fitness of the population in time interval $(t_{k-1}, t_k)$ for all $k \geq 1$ | |
| $n_{ik}$ | size of $i$th lineage at $t_k$ before dilution | |
| $r_{ik}$ | barcode read count of $i$th lineage at $t_k$ | |
| $\boldsymbol{r}_{ik}$ | observation vector for lineage $i$ prior to and including $t_k$, $\boldsymbol{r}_{ik} = (r_{i0}, \ldots, r_{ik})$ | |
| $P^\mathrm{b}(n, s\|\boldsymbol{r})$ | belief probability that a lineage with the observation vector $\boldsymbol{r}$ has size $n$ and fitness $s$ | (3), (S41) |
| $P^\mathrm{b}_{ik}(n, s)$ | shorthand for $P^\mathrm{b}(n, s\|\boldsymbol{r}_{ik})$ | |
| $P^\mathrm{m}(r\|n)$ | probability of observing $r$ reads for a lineage with size $n$. $P^\mathrm{m}$ depends on the total read depth $R$ and the measurement error parameter $\epsilon$ | (S25) |
| $P^\mathrm{pr}(n, s\|\boldsymbol{r}')$ | prior probability that a lineage with the observation vector $\boldsymbol{r}'$ has size $n$ and selection coefficient $s$ at the *next* sampling time point. $P^\mathrm{pr}$ depends on the current mean fitness $\bar{s}$ | (S39) |
| $Z^N(r_{ik})$ | marginal likelihood of $r_{ik}$ in the neutral model. $Z^N$ depends on the current mean fitness $\bar{s}$ and measurement error parameter $\epsilon$ | (S61) |
| $Z^S(r_{ik})$ | marginal likelihood of $r_{ik}$ in the selection model. $Z^S$ depends on the current mean fitness $\bar{s}$ and measurement error parameter $\epsilon$ | (S42) |
| $L_k(\bar{s}, \epsilon)$ | log-likelihood function for the mean fitness $\bar{s}$ and measurement error parameter $\epsilon$ | (S58) |

**Table S4.** Notations used in BASIL.

| Strain | Cond. | Ref. | Mean | Median | IQR | IDR |
|---|---|---|---|---|---|---|
| HY0 | C-2d | [2] | 0.12* | 0.05* | 0.02 (0.060) | 0.11* |
| HY0 | N-2d | [2] | 0.48* | 0.51* | 0.15* | 0.25* |
| HY0 | C-1d | [6] | 1.85* | 1.74* | 1.31* | 0.72* |
| HY0 | C-5d | [6] | 2.01* | 2.35* | 0.31 (0.081) | 0.15 (0.48) |
| HY1 | C-2d | [9] | 0.01* | 0.11* | 0.13* | 0.79* |
| HY2 | C-2d | [9] | 0.41* | 0.30* | 0.60* | 0.67* |
| HY3 | C-2d | [9] | 0.05* | 0.05* | 0.22* | 0.15* |
| DY | N-5d | [1] | 0.74* | 0.66* | 0.98* | 1.49* |
| DY | N-5d+*Cr* | [1] | 0.43* | 0.43* | 0.16 (0.070) | 0.34* |

**Table S5.** Differences of mDFE statistics across replicates. For each mDFE statistic, we report the absolute value of the pairwise difference in the statistic value, averaged across all pairs of replicates (see text for details). * indicates $P$-value $< 0.01$ (permutation test); $P$-values $\geq 0.01$ are shown in parentheses.

| | Full | Reduced coverage | Reduced sampling |
|---|---|---|---|
| # of samples | 12 | 12 | 8 |
| Coverage | 158 | 10 | 158 |
| # of adapt. lin., $\times 10^3$ | 61 | 49 | 48 |
| Min $s_i$, % | 0.43 | 0.56 | 0.63 |
| Max $s_i$, % | 11.49 | 11.67 | 10.85 |
| Mean $s_i$, % | 1.87 | 2.12* | 1.96* |
| $s_i$ IQR, % | 1.16 | 1.13* | 1.10* |
| $s_i$ IDR, % | 2.12 | 2.05* | 2.05* |

**Table S6.** Effects of down-sampling on the characteristics of the measured distribution of fitness effects of new mutations (mDFE). The "Full" dataset is Replicate 1 of the HY0 strain in C-1d environment [2]. Notations are as in Table 2 in the main text. See Section 6.3 for details. *indicates $P < 0.01$ for the differences between the full and reduced data (permutation test). Statistical significance was tested only for the mean, IQR and IDR.
